# Systematic comparison of CRISPR-based transcriptional activators uncovers gene-regulatory features of enhancer–promoter interactions

**DOI:** 10.1093/nar/gkac582

**Published:** 2022-07-18

**Authors:** Kaiyuan Wang, Mario Escobar, Jing Li, Barun Mahata, Jacob Goell, Spencer Shah, Madeleine Cluck, Isaac B Hilton

**Affiliations:** Department of Bioengineering, Rice University, Houston, TX 77005, USA; Department of BioSciences, Rice University, Houston, TX 77005, USA; Department of Bioengineering, Rice University, Houston, TX 77005, USA; Department of Bioengineering, Rice University, Houston, TX 77005, USA; Department of Bioengineering, Rice University, Houston, TX 77005, USA; Department of Bioengineering, Rice University, Houston, TX 77005, USA; Department of BioSciences, Rice University, Houston, TX 77005, USA; Department of Bioengineering, Rice University, Houston, TX 77005, USA; Department of BioSciences, Rice University, Houston, TX 77005, USA

## Abstract

Nuclease-inactivated CRISPR/Cas-based (dCas-based) systems have emerged as powerful technologies to synthetically reshape the human epigenome and gene expression. Despite the increasing adoption of these platforms, their relative potencies and mechanistic differences are incompletely characterized, particularly at human enhancer–promoter pairs. Here, we systematically compared the most widely adopted dCas9-based transcriptional activators, as well as an activator consisting of dCas9 fused to the catalytic core of the human CBP protein, at human enhancer–promoter pairs. We find that these platforms display variable relative expression levels in different human cell types and that their transactivation efficacies vary based upon the effector domain, effector recruitment architecture, targeted locus and cell type. We also show that each dCas9-based activator can induce the production of enhancer RNAs (eRNAs) and that this eRNA induction is positively correlated with downstream mRNA expression from a cognate promoter. Additionally, we use dCas9-based activators to demonstrate that an intrinsic transcriptional and epigenetic reciprocity can exist between human enhancers and promoters and that enhancer-mediated tracking and engagement of a downstream promoter can be synthetically driven by targeting dCas9-based transcriptional activators to an enhancer. Collectively, our study provides new insights into the enhancer-mediated control of human gene expression and the use of dCas9-based activators.

## INTRODUCTION

The programmability of CRISPR/Cas-based nucleases has revolutionized genome engineering and democratized the ability to edit genomic sequences within living cells (1–4). In parallel, nuclease-null deactivated, ‘dCas’ proteins have been developed that retain the ability to precisely target genomic DNA without genetic disruption after targeting, and these dCas-based platforms can be used as versatile, RNA-guided DNA binding domains (5–7). This capability has catalyzed a rapid emergence of new tools to recruit different transcriptional or epigenetic effector domains to specific genomic loci and thereby engineer endogenous gene expression (8–15) and/or epigenomic modifications (16–23), largely based upon the deactivated Cas9 protein from *Streptococcus pyogenes* (dCas9) and its associated guide RNA (gRNA). Several different dCas9- or gRNA-based recruitment methods have been developed to site-specifically control the precise genomic localization of transcriptional activators and/or epigenetic effectors. These different recruitment strategies include direct fusions between the effector protein and the N or C termini of dCas9 (5,8,10,14,16,19), antibody-based recruitment via binding to the cognate polypeptide(s) directly fused to dCas9 (12), and through the use of RNA aptamers that recognize engineered gRNA structures (13,24).

While most studies using dCas9-based transcriptional activators have focused on a single type of effector domain or effector recruitment architecture and/or a single cell type or locus for analysis, a handful of comparative analyses have been performed. Although these important studies have demonstrated that there are functional differences in the relative transactivation potencies among widely adopted dCas-based activators (25–28), there remains a paucity of comprehensive comparisons between dCas9-based transcriptional activators and this deficit can make the selection of the optimal activator needed to achieve a specific experimental outcome low-throughput and technically difficult (29,30). In addition, an increased understanding of the mechanisms by which dCas9-based transcriptional activators induce cellular transcription and greater clarity surrounding the parameters that drive differences in their relative efficacies are needed to fully leverage these technologies for the careful dissection and engineering of human gene regulation.

Multiple different epigenomic and transcriptional mechanisms converge to dynamically orchestrate human gene regulation (31–36). Enhancer-mediated activation of promoters is one mechanism critical for the expression of human genes. Although still incompletely understood, enhancer-mediated activation of promoters has been associated with the production of non-coding enhancer RNAs (eRNAs) (37–42), changes in the acetylation status of histone lysine residues (43–45), and increased physical contact frequency between enhancers and cognate promoters (46–49). Further, recent reports have demonstrated a positive correlation between eRNA induction and mRNA output using dCas9-based transcriptional activators (42). Here, to more thoroughly understand the enhancer-mediated activation of promoters in a greater spectrum of native chromatin contexts and conditions, we comprehensively compared the transactivation potencies of a set of the most widely used dCas9-based transcriptional activators across three enhancer–promoter pairs in three different human cell lines. We also assayed histone subunit 3 lysine 27 acetylation (H3K27ac) levels, eRNA and intergenic noncoding RNA (ncRNA) expression levels, enhancer–promoter contact frequency, and mRNA expression at, and between, the HS2-*HBG1* enhancer–promoter pair within the human β-globin locus (50–56) in different human cell lines. Using several dCas9-based transcriptional activators, we demonstrate that the targeted activation of human eRNAs is positively correlated with mRNA synthesis from downstream promoters. We also show that an intrinsic transcriptional and epigenetic reciprocity can exist between human enhancers and promoters, and that a transcriptional tracking mechanism appears to coordinate activity between the HS2-*HBG1* enhancer–promoter pair. Altogether our studies refine the rules of use for commonly used dCas9-based transcriptional activators and highlight the utility of these technologies for the *in situ* dissection of human gene regulatory mechanisms.

## MATERIALS AND METHODS

### Cell culture and plasmid construction

HEK293T cells (ATCC, CRL-11268) and HeLa cells (ATCC, CCL-2) were cultured in Dulbecco's modified Eagle's medium (DMEM; Gibco, 31-053-028) supplemented with 10% FBS (Sigma, F2442) and 1% penicillin/streptomycin (Gibco, 15140). K562 cells (ATCC, CRL-243) were cultured in Roswell Park Memorial Institute 1640 medium (RPMI 1640; Gibco, 11-875-119) supplemented with 10% FBS (Sigma, F2442) and 1% penicillin/streptomycin (Gibco, 15140). U2OS cells (ATCC, HTB-96) were cultured in McCoy's 5A (modified) medium (Gibco, 16-600-082) supplemented with 10% FBS (Sigma, F2442) and 1% penicillin/streptomycin (Gibco, 15140). All cell lines were maintained at 37 °C and 5% CO_2_.

Plasmids encoding dCas9-p300 (Addgene, 83889), the dCas9-SAM system (Addgene, 61423 and 61425) and the dCas9-SunTag system (Addgene, 60903 and 60904) were obtained from Addgene. A FLAG tag sequence (see Supplemental Table S1 for all cloning primers) was added to the dCas9-SAM system by digesting Addgene plasmids 61423 and 61425 with NheI (NEB, R3131S) and BsrGI (NEB, R3575S), respectively using NEBuilder HiFi DNA Assembly (NEB, E2621). A FLAG tag sequence was added to the dCas9-SunTag system by digesting Addgene plasmids 60903 and 60904 with NotI (NEB, R3189L) and SpeI (NEB, R3133S), respectively, using NEBuilder HiFi DNA Assembly (NEB, E2621).

Amino acids 1084–1701 (3249–5103 nt) of the human CBP protein (the histone acetyltransferase core region) were synthesized as a gBlock gene fragment (Integrated DNA Technologies) and then subcloned into the BamHI-digested pLV-dCas9-p300-P2A-Puro backbone (Addgene, 83889) via NEBuilder HiFi DNA Assembly (NEB, E2621) to create the pLV-dCas9-CBP-P2A-Puro construct (dCas9-CBP). The pLV-EFS-dCas9-VPR-P2A-Puro (dCas9-VPR) was generated by amplifying the VPR effector sequence from Addgene plasmid 63798 and then appending a FLAG tag sequence using PCR. The resulting PCR product was subcloned into a BamHI-digested pLV-dCas9-p300-P2A-Puro backbone (Addgene, 83889) via NEBuilder HiFi DNA Assembly (NEB, E2621). Protein sequences of all dCas9 constructs are shown in Supplemental Note 1. All gRNAs used for dCas9, dCas9-VPR, dCas9-SunTag, dCas9-p300, dCas9-CBP experiments were cloned into the pSPgRNA backbone (Addgene, 47108). All gRNAs used for dCas9-SAM experiments were cloned into the sgRNA (MS2) cloning backbone (Addgene, 61424). All gRNA protospacers sequences are listed in Supplemental Table S2. pmaxGFP (Lonza; V4XC-2012) was used to determine transfection efficiencies across transfection methods and cell lines.

### Plasmid delivery

Transient transfections were performed in 24-well plates for HEK293T (1.2e5 cells per well), HeLa (0.5e5 cells per well) and U2OS cells (0.5e5 cells per well) using Lipofectamine 3000 (Invitrogen, L3000015) as per manufacturer's instruction. The total mass of plasmids delivered in all lipofectamine-based transient transfections was ∼500 ng/well. 166ng of respective dCas9 expression plasmid (dCas9, dCas9-VPR, dCas9-p300, dCas9-CBP, dCas9-SAM or dCas9-SunTag) was used. For dCas9, dCas9-VPR, dCas9-p300 and dCas9-CBP experiments, 166 ng of pooled gRNA expression vectors and 166 ng of pUC19 filler plasmid (Addgene, 50005) were included in transient transfection mixes. For dCas9-SAM and dCas9-SunTag experiments, 166ng of secondary activator fusion components (i.e. MS2-P65-HSF1 and scFv-GCN4-sfGFP-VP64, respectively) were used instead of pUC19 filler plasmid. Nucleofection experiments were performed in 6-well plates for K562 cells (1.0e6 cells) using the Lonza SF Cell Line 4D-Nucleofector Kit (Lonza V4XC-2012) and a Lonza 4D Nucleofector (Lonza, AAF1002X). 2000 ng of total plasmid DNA was nucleofected in each experiment using 1.0e6 K562 cells with the same setup as transient transfection, except that the amount of each type of nucleofected plasmid was upscaled accordingly to ∼667ng. For 3C-qPCR, all transfections and nucleofections were scaled up accordingly for a final cell number of 1.0e7. For titration experiments in K562 cells, 400 ng of total plasmid DNA (100 ng of pooled gRNA encoding plasmids, indicated amounts of dCas9-VPR encoding plasmid, and pUC19 filler plasmid added to 400 ng total) was nucleofected into 2.0e5 K562 cells using the Lonza SF Cell Line 4D-Nucleofector Kit (V4XC-2032).

### Western blot analysis

Cells were lysed 72 h post-transfection in RIPA buffer (Thermofisher, 89900) with protease and phosphatase inhibitor cocktail (Thermofisher, 78442) added as per manufacturer's instruction. 50 μg of protein lysate was loaded and run using SDS PAGE and then transferred onto a PVDF membrane (Bio-Rad, 1704274) using the Bio-Rad Trans-Blot turbo transfer system (Bio-Rad 1704150). Primary antibodies (anti-Cas9: Diagenode C15200216, anti-tubulin: Bio-Rad, 12004166) were used at a 1:1000 dilution in 1× TBS with 1% Casein. Secondary anti-mouse StarBright blue (Bio-Rad, 12004159) was used at a 1:2000 dilution in 1× TBS with 1% Casein. Membranes were imaged for StarBright Blue and Rhodamine chemiluminescence signal on the Bio-Rad ChemiDoc MP imaging system (Bio-Rad 12003154).

### Reverse-transcription quantitative PCR (RT-qPCR)

RNA was extracted from cells 72 h post-transfection or post-nucleofection with Qiagen RNeasy Plus mini kit (Qiagen, 74136). 500ng-1μg of RNA was used for cDNA synthesis using the iScript advanced cDNA synthesis kit (Bio-Rad, 1725038). Real-time quantitative PCR was performed using SYBR Green (Bio-Rad, 1725275). Luna Universal qPCR Master Mix (NEB, M3003E) was used for selected titration experiments on a CFX96 Real-Time PCR Detection System with a C1000 Thermal Cycler (Bio-Rad, 1855195). Baselines were subtracted using the baseline subtraction curve fit analysis mode and thresholds were automatically calculated using the Bio-Rad CFX Manager software (version 2.1). Results are expressed as fold change above cells transfected with the dCas9 control plasmid after normalization to GAPDH expression using the ΔΔCt method, as previously described (23). All qPCR primers and conditions are listed in Supplemental Table S3.

### CUT&RUN assays

CUT&RUN assays were carried out using the Epicypher CUTANA ChIC/CUT&RUN Kit (Epicypher 14-1048). Briefly, 5.0e5 transfected cells were harvested, immobilized on concanavalin A beads, and permeabilized in 0.01% digitonin cell permeabilization buffer. The cell-bead conjugate mixture was then divided equally into 2 aliquots and incubated in 50 μl antibody buffer with either 0.5 μg of anti-H3K27ac antibody (Abcam, 4729) or 0.5 μg of control rabbit IgG antibody (Abcam, 37415) overnight at 4°C. After washing the beads, pAG-MNase was added to the immobilized cells and the solution was incubated for 2 h at 4°C to digest and release chromatin DNA. For CUT&RUN-qPCR assays, 1μl of purified DNA from both H3K27ac antibody treatment and rabbit IgG control treatment was then assayed using qPCR. Relative enrichment of H3K27ac is expressed as fold change above cells transfected with dCas9 control plasmid after normalization to purified DNA in rabbit IgG control samples using the ΔΔCt method. All qPCR primers and conditions are listed in Supplemental Table S3.

For CUT&RUN followed by next-generation sequencing (CUT&RUN-Seq) assays, all steps were performed as above, but the IgG antibody used was from Epicypher (14–1048) and after the release of chromatin DNA, samples were shipped to Genewiz (NJ, USA) in EB buffer on dry ice. Sample QC, sequencing and initial bioinformatics were performed at GENEWIZ. Libraries were prepared using the NEBNext Ultra II DNA Library Prep Kit for Illumina kit (NEB, E7645) and then sequenced using an Illumina HiSeq 4000 where base calls were performed using bcl2fastq (v2.17). The sequencing libraries were validated by Agilent TapeStation and quantified using Qubit 2.0 Fluorometer (Invitrogen). Reads were trimmed using Trim Galore (v0.6.6) and aligned to the hg38 reference genome using Bowtie2 (v7.3.0) (57). Aligned reads were deduplicated using Picard MarkDuplicates (v2.23.8) and converted into a bedGraph file using bedtools (v2.30.0) (58). Genome-wide H3K27ac analysis was performed similar to previously reports (59) except that peaks were called using SEACR (v1.3) (60) with the deduplicated alignments normalized to IgG pooled control. Peaks from biological replicates were then merged using bedops (v2.4.39) (61) and count tables with reads in peaks were calculated using bedtools multicov (v2.30.0) (58). The difference in H3K27ac was finally assessed and plotted using R (v4.1.3) and DESeq2 (v1.34.0) (62) with an FDR cutoff of ≤0.05.

### 3C-qPCR

1.0e7 cells were harvested 72 h post-transfection, crosslinked in 9.5 ml of 2% formaldehyde in PBS with 10% FBS (Sigma-Aldrich, F8775) and then incubated at room temperature for 10 min. 1.425 ml of ice-cold 1M glycine was added to a final concentration of 130 mM glycine to quench crosslinking similar to previous methods (63). Briefly, crosslinked cells were then pelleted and then lysed in 5ml of lysis buffer (10 mM Tris–HCl, pH 7.5; 10mM NaCl; 5 mM MgCl_2_; 0.1 mM EGTA; 1× complete protease inhibitor; Fisher, A32965) on ice for 10 min, and then centrifuged for 5 min at 400g at 4°C for nuclei extraction. The extracted nuclei were transferred to 1× CutSmart digestion buffer (NEB, B7204) with 0.3% SDS (Invitrogen 24730020) and then shaken at 900 RPM for 1 h at 37°C, after which, 2% Triton X-100 (Sigma-Aldrich, T9284) was added, and another 1 h incubation with shaking at 900RPM at 37°C was performed. 400 U of HindIII (NEB R0104) was then added, and the nuclear DNA was digested at 37°C while shaking at 900RPM overnight. The solution was then brought to 7 ml total volume in T4 ligation buffer (NEB B0202) with a final concentration of 1.6% SDS and 1% Triton X-100. The mixture was then gently shaken at 37°C for 1 h. 100 Weiss units of T4 DNA ligase (NEB M0202) was then added to the nuclear DNA, and the solution was ligated for 4 h at 16°C and then for 30 min at room temperature. The reaction was then incubated with 300 ug of proteinase K (Qiagen 19131) at 65°C overnight. The next day 300 ug of RNase (Qiagen 9101) was added and the solution was incubated at 37°C for 45 min. The nuclear DNA was then purified using phenol–chloroform (Fisher BP1752I400) extraction. 1000 ng of extracted DNA was used for qPCR. Results are expressed as fold change above cells transfected with the dCas9 control plasmid after normalization to GAPDH expression using the ΔΔCt method. The basal relative crosslinking frequency between the HS2 enhancer and selected locations spanning the 30 kb between HS2 and the *HBG1* promoter was determined similar to previous reports (55,63) by comparing ligated genomic DNA with control target DNA. Interaction between fragments within the GAPDH gene was used as the internal normalization control for 3C signals, and the highest frequency crosslinked to the HS2 site was set as 100%. All qPCR primers and conditions are listed in Supplemental Table S3.

### ChIP-qPCR

HEK293T cells were co-transfected with indicated dCas9 fusion expression vectors and gRNA constructs in 10cm plates in biological triplicates for each condition tested. Cells were cross-linked for 10 min at RT using 1% formaldehyde (Sigma F8775-25ML) and then the reaction was stopped by the addition of glycine to a final concentration of 125 mM. Cells were harvested and washed with ice cold 1× PBS and suspended in Farnham lysis buffer (5 mM PIPES pH 8.0, 85 mM KCl, 0.5% NP-40) supplemented with protease inhibitor (Thermo Scientific, A32965). Cells were then pelleted and resuspended in RIPA buffer (1X PBS, 1% NP-40, 0.5% sodium deoxycholate, 0.1% SDS) supplemented with protease inhibitor. Approximately 2.5e7 cells were used for each ChIP experiment. Chromatin in RIPA buffer was sheared to a median fragment size of ∼250 bp using a Bioruptor XL (Diagenode). 2 μg of each antibody (α-FLAG (Sigma-Aldrich, F1804), Mouse IgG (Abcam, ab18413) was incubated with 50 μl Mouse IgG magnetic beads (Life Technologies, 11202D) for 16 h at 4°C. Antibody-linked magnetic beads were washed 3 times with PBS/BSA buffer (1× PBS and 5 mg/ml BSA) and sheared chromatin was incubated with corresponding antibody-linked magnetic beads at 4°C overnight and then washed 5 times with LiCl IP wash buffer (100 mM Tris pH 7.5, 500 mM LiCl, 1% NP-40, 1% sodium deoxycholate). Cross-links were then reversed via overnight incubation at 65°C and DNA was purified using QIAquick PCR purification kit (Qiagen, 28106) for ChIP-qPCR. Input DNA was prepared from ∼1.0e6 cells. 10 ng of DNA was used for subsequent qPCR reactions using a CFX96 Real-Time PCR Detection System with a C1000 Thermal Cycler (Bio-Rad, 1855195). Baselines were subtracted using the baseline subtraction curve fit analysis mode and thresholds were automatically calculated using the Bio-Rad CFX Manager software version 2.1. ChIP-qPCR data is quantified relative to percent input and normalized by the non-targeting sample for each dCas9 fusion. All ChIP-qPCR primers and conditions are listed in Supplementary Table S3.

## RESULTS

### Target locus and cell type impact the functionality of dCas9-based transcriptional activators

**Figure 1.**
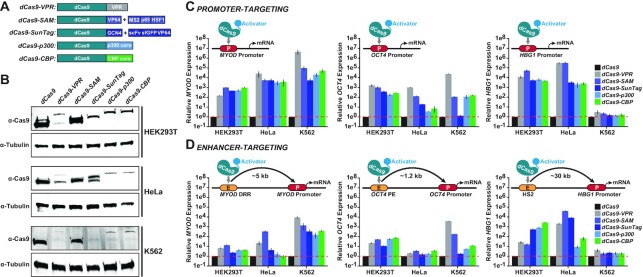
dCas9-based transcriptional activators display variable expression and transactivation potencies based upon fused effector system, cell type and targeted locus. (**A**) The dCas9-VPR, dCas9-SAM, dCas9-SunTag, dCas9-p300 and dCas9-CBP transcriptional activation systems are schematically depicted. (**B**) Expression levels of indicated dCas9-based transcriptional activators 72 h after transient transfection into HEK293T (top), HeLa (middle), or K562 (bottom) cells. (**C**) Relative *MYOD* (left), *OCT4* (middle) or *HBG1* (right) mRNA levels 72 h after transient transfection with indicated dCas9-based transcriptional activators, respective promoter-targeted gRNAs, and in specified cell lines (*n* = 3 independent replicates, error bars indicate ± SEM). (**D**) Relative *MYOD* (left), *OCT4* (middle) or *HBG1* (right) mRNA levels 72 h after transient transfection with indicated dCas9-based transcriptional activators, respective enhancer-targeted gRNAs and in specified cell lines (*n* = 3 independent replicates, error bars indicate ± SEM).

To systematically compare the relative transactivation potential of different dCas9-based transcriptional activators, we tested five different platforms (Figure 1A). dCas9-VPR (14), dCas9-SAM (13), dCas9-SunTag (12) and dCas9-p300 (16) are widely used dCas9-based transcriptional activators. We also constructed a novel fusion protein consisting of the catalytic core of the human CBP protein (dCas9-CBP) fused to the C-terminus of dCas9 for testing, as dCas9 fused to the catalytic core of the human p300 protein (which is highly homologous to human CBP) previously showed potent activation of both human enhancers and promoters (16). Although dCas9-CBP fusion proteins have been developed previously using the murine (26) and *Drosophila* (27) versions of the CBP protein, we designed a version of dCas9-CBP to include CBP sequences solely of human origin (Supplemental Figure S1). We also investigated the transactivation potential of other human histone acetyltransferase (HAT) proteins when fused to dCas9, including GCN5 (64–66), minimized human HAT domains, and bipartite combinations of human HAT proteins (Supplemental Figures S2–S5). However, none of these variants displayed improved transactivation potency relative to dCas9-p300 or dCas9-CBP.

We targeted each selected dCas9-based transcriptional activator to three enhancer–promoter pairs in three different human cell lines: HEK293T, HeLa and K562. Notably, the fusion of VPR, the GCN4 component of the SunTag system, p300 or CBP to dCas9 resulted in decreased dCas9-fusion protein levels relative to dCas9 in each of these tested human cell lines, most dramatically in K562 cells (Figure 1B, Supplemental Figure S6). Further, as has been observed previously (67,68), multiple bands were detected via Western blot for dCas9-VPR and dCas9-SunTag systems. These bands were not the result of impure plasmid preparations (Supplemental Figure S7) and interestingly, were not observed when detecting a C-terminal fused FLAG epitope (Supplemental Figure S6). Regardless, each fusion protein maintained the ability to activate transcription from human promoters and enhancers (Figure 1C and D) as shown previously (12–14,16,25,28,69). In fact, even with decreased input plasmid amounts, high levels of transactivation were observed with dCas9-VPR despite low Western blot signals (Supplemental Figure S8). Notably, the HS2 enhancer and *HBG1* promoter in K562 cells displayed relatively low responsiveness to dCas9-based transcriptional activation (Figure 1C and D), which we attribute to the high basal expression of *HBG1* in K562 cells (70) (Supplemental Figure S9).

When each dCas9-based transcriptional activator was targeted to enhancers known to govern the expression of *MYOD*, (the *MYOD* Distal Regulatory Region; DRR) (71), *OCT4* (the *OCT4* proximal enhancer; PE) (72) or *HBG1* (the HS2 enhancer) (53,54), in most cases (32 out of 45 enhancer-targeting experiments—across three cell types using three different targets and five different activators; 71.1%, Figure 1D, Supplemental Table S4), significant activation relative to dCas9 control-treated cells was observed across HEK293T, HeLa and K562 cells. In addition, in most of these cases (38 out of 40 loci, excluding the relatively unresponsive *HBG1* locus in K562 cells; 95%) targeting a gene's promoter region resulted in higher relative transactivation than targeting a corresponding enhancer (Figure 1C and D, Supplemental Table S4). Except for the relative transactivation potency displayed by dCas9-VPR in K562 cells, there were no clear generalities regarding which dCas9-based transcriptional activator was the most effective at transactivation based upon cell type or locus (Figure 1C and D). For example, although the dCas9-SAM system induced potent activation from the *HBG1* promoter in HEK293T cells, dCas9-VPR was measurably more effective at the *OCT4* promoter in HEK293T cells (Figure 1C, Supplemental Table S4).

Moreover, even for enhancer–promoter pairs, the most effective dCas9-based transcriptional activator was generally variable. For instance, in HEK293T cells, although dCas9-SAM resulted in the highest measured activation of *HBG1* at the promoter region, dCas9-CBP was the most potent in activating *HBG1* expression when targeted to the HS2 enhancer region (Figure 1C and D; Supplemental Table S4). Together, these data demonstrate that the relative protein expression levels and abilities of dCas9-based transcriptional activators to induce gene expression are influenced by cell type- and locus-specific factors. These results also suggest that, for applications where maximally increasing mRNA expression is the goal, promoters are generally more responsive when targeted by dCas9-based transcriptional activators than respective cognate enhancers (see Supplemental Table S4). Importantly, the differences observed between cell types tested here were not due to major differences in relative transfection efficiencies (Supplemental Figure S10).

### dCas9-based transcriptional activators induce enhancer RNAs when targeted to the HS2 enhancer coincident with downstream *HBG1* mRNA production

**Figure 2.**
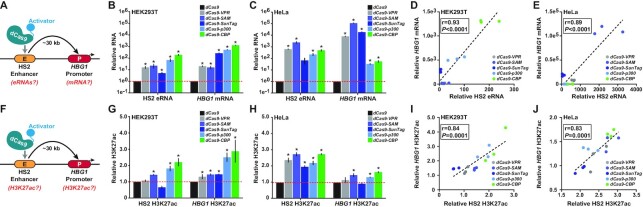
dCas9-based transcriptional activators induce RNA synthesis and influence histone acetylation locally and at the *HBG1* promoter when targeted to the HS2 enhancer. (**A**) The targeting and RNA assay strategy for dCas9-based transcriptional activators after localization to the HS2 enhancer is schematically depicted. (**B** and **C**) Relative RNA expression from the HS2 enhancer and downstream *HBG1* promoter 72 h post-transfection with 4 HS2-targeting gRNAs and the indicated dCas9-based transcriptional activators is shown for HEK293T and HeLa cells, respectively (*n* = 3 independent replicates, error bars indicate +/- SEM, * indicates adjusted *P*-value <0.05 compared to dCas9 control-treated cells using two-tailed Student *t*-test and correcting for multiple comparisons and false discovery rate (FDR) using two-stage linear step-up procedure of Benjamini, Krieger and Yekutieli). (**D** and **E**) The data points from Figure 2B and C, respectively; are presented as scatter plots with Pearson correlation coefficients (*r*) and *P-*values (calculated using two-tailed Student *t*-test) indicated. (**F**) The targeting and H3K27ac assay strategy for dCas9-based transcriptional activators after localization to the HS2 enhancer is schematically depicted. (**G** and **H**) Relative enrichment of H3K27ac at the HS2 enhancer and downstream *HBG1* promoter 72 h post-transfection with 4 HS2-targeting gRNAs and the indicated dCas9-based transcriptional activators is shown for HEK293T and HeLa cells, respectively (*n* = 3 independent replicates, error bars indicate ± SEM, * indicates adjusted *P*-value <0.05 compared to dCas9 control-treated cells using two-tailed Student *t*-test and correcting for multiple comparisons and FDR using two-stage linear step-up procedure of Benjamini, Krieger and Yekutieli). (**I** and **J**) The data points from (G) and (H), respectively; are presented as scatter plots with Pearson correlation coefficients (*r*) and *P-*values (calculated using two-tailed Student *t*-test) indicated.

Enhancer-mediated control of human gene expression is complex (44,73). For instance, the transcription of noncoding RNAs from enhancers (eRNAs) has been observed to correlate with downstream mRNA synthesis from corresponding promoters (40,42). Since our data demonstrated that dCas9-based transcriptional activators can have different relative potencies at enhancers and cognate promoters (Figure 1C and D; Supplemental Table S4), we further explored the relationship between synthetic enhancer activation and downstream mRNA expression. To do so, we focused on the human β-globin control region as a testbed because this locus has well-established links to human disease, displays cell-type-specific activity, and is a longstanding model of enhancer-mediated transcriptional control (49–56,74). We targeted dCas9, dCas9-VPR, dCas9-SAM, dCas9-SunTag, dCas9-p300 or dCas9-CBP to the HS2 enhancer in HEK293T and HeLa cells (Figure 2). K562 cells were omitted from analysis as the β-globin locus is highly active and *HBG1* is highly expressed in K562 cells (70) (Supplemental Figure S9), rendering the locus generally unresponsive to dCas9-based transcriptional activators in K562 cells (Figure 1C and D).

In most cases (8 out of 10 experiments; 80%), dCas9-based transcriptional activators induced significant eRNA synthesis when targeted to the HS2 enhancer in either HEK293T cells or HeLa cells (Figure 2B and C). Interestingly, relative eRNA production from the HS2 enhancer in response to different dCas9-based transcriptional activators showed high positive correlations with relative mRNA production from the *HBG1* promoter (Figure 2D and E; Pearson correlation coefficient *r* = 0.93 and *P*-value = 6.2e−7 for HEK293T cells, and Pearson correlation coefficient *r* = 0.89 and *P*-value = 1.1e−5 for HeLa cells, respectively; see also Supplemental Figure S11). These data demonstrate that dCas9-based transcriptional activators can induce local RNA synthesis when targeted to different classes of human regulatory elements (i.e. either enhancers or promoters), and furthermore show that the local production of eRNAs from an enhancer can positively correlate with the activation of mRNA from a corresponding downstream promoter in native human chromatin. Similar results were obtained when targeting the HS2 enhancer in U2OS cells (Supplementary Figure S12) and when targeting the KLK3 enhancer in HEK293T and HeLa cells (Supplementary Figure S13).

### dCas9-based deposition of H3K27ac at the HS2 enhancer is correlated with increased H3K27ac at the *HBG1* promoter

Similar to eRNA levels, high levels of H3K27ac have also been linked to active human enhancers (43,75–77). In addition, dCas9-based tools have been developed that enable the deposition of H3K27ac at endogenous genomic loci (16,27,69). These technologies have been used to study the function of acetylation at enhancers, and moreover, H3K27ac has been used as a proxy for their functional efficacy. To more systematically dissect the potential role of H3K27ac in governing the enhancer-mediated control of human gene expression, we targeted dCas9, dCas9-VPR, dCas9-SAM, dCas9-SunTag, dCas9-p300 or dCas9-CBP to the HS2 enhancer and then measured resulting changes in H3K27ac at HS2 enhancer and downstream *HBG1* promoter (Figure 2F). In HEK293T cells, targeting the dCas9-SAM system, dCas9-p300, or dCas9-CBP to the HS2 enhancer resulted in significant enrichment of H3K27ac at the targeted site (Supplemental Figure S14) relative to dCas9 control (Figure 2G). Interestingly, dCas9-VPR and dCas9-SunTag systems did not induce significant increases in local H3K27ac levels when targeted to the HS2 enhancer in HEK293T cells. In contrast, in HeLa cells, all dCas9-based transcriptional activators tested resulted in significant increases in local H3K27ac levels at the HS2 enhancer target site (Figure 2H). These data demonstrate that dCas9-based transcriptional activators can deposit H3K27ac at an endogenous human enhancer, but the efficacy of this deposition varies based upon the effector domain fused to dCas9 and upon cell type.

Since enhancers can physically engage cognate promoters to regulate gene expression (49,52,54,55), we also measured the H3K27ac levels at the *HBG1* promoter after targeting the HS2 enhancer with dCas9, dCas9-VPR, dCas9-SAM, dCas9-SunTag, dCas9-p300 or dCas9-CBP (Figure 2F–H). Targeting the HS2 enhancer with either dCas9-SAM, dCas9-SunTag, dCas9-p300 or dCas9-CBP resulted in significant enrichment of H3K27ac at the *HBG1* promoter in HEK293T cells (Figure 2G). In HeLa cells, targeting the HS2 enhancer with either dCas9-SAM, dCas9-p300 or dCas9-CBP also resulted in significant enrichment of H3K27ac at the *HBG1* promoter (Figure 2H). Correlation analysis among all conditions tested revealed that the relative enrichment of H3K27ac at the HS2 enhancer was positively correlated to the relative enrichment at the downstream *HBG1* promoter (Pearson correlation coefficients = 0.84 and 0.83, and *P*-values = 1.1e–4 and 1.2e–4 for HEK293T and HeLa cells, respectively; Figure 2I and J). In addition, targeting the HS2 enhancer with dCas9-based transcriptional activators in HEK293T cells resulted in a positive correlation between the relative enrichment of H3K27ac at the HS2 enhancer and HS2 eRNA levels (Pearson correlation coefficient = 0.82, *P*-value = 1.8e–4), and to a lesser extent, with downstream *HBG1* mRNA expression levels (Pearson correlation coefficient = 0.72, *P*-value = 2.5e−3; Supplemental Figure S15A). However, similar analyses in HeLa cells showed a much weaker correlative relationship between H3K27ac deposited at the HS2 enhancer and levels of HS2 eRNAs or *HBG1* mRNA (Pearson correlation coefficients = 0.55 and 0.40, and *P*-values = 0.035 and 0.14 respectively; Supplemental Figure S15B). Nevertheless, these data demonstrate that induced H3K27ac levels at the HS2 enhancer are positively correlated to increased H3K27ac enrichment at the downstream *HBG1* promoter in both HEK293T and HeLa cells. However, these results suggest that the correlations between H3K27ac and the production of RNA from HS2 and *HBG1* are more dynamic and are not necessarily predictive of transcriptional increases.

### Targeted transcriptional activation of the *HBG1* promoter can induce eRNAs from the HS2 enhancer

**Figure 3.**
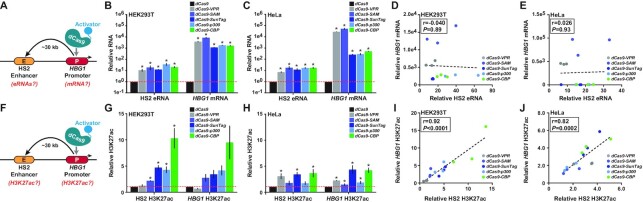
Targeted perturbation of the *HBG1* promoter using dCas9-based transcriptional activators influences RNA synthesis and histone acetylation at the upstream HS2 enhancer. (**A**) The targeting and RNA assay strategy for dCas9-based transcriptional activators after localization to the *HBG1* promoter is schematically depicted. (**B** and **C**) Relative RNA expression from the HS2 enhancer and downstream *HBG1* promoter 72 h post-transfection with 4 *HBG1*-targeting gRNAs and the indicated dCas9-based transcriptional activators is shown for HEK293T and HeLa cells, respectively (*n* = 3 independent replicates, error bars indicate ± SEM, * indicates adjusted *P*-value < 0.05 compared to dCas9 control-treated cells using two-tailed Student *t*-test and correcting for multiple comparisons and false discovery rate (FDR) using two-stage linear step-up procedure of Benjamini, Krieger and Yekutieli). (**D** and **E**) The data points from (B) and (C), respectively; are presented as scatter plots with Pearson correlation coefficients (*r*) and *P-*values (calculated using two-tailed Student *t*-test) indicated. (**F**) The targeting and H3K27ac assay strategy for dCas9-based transcriptional activators after localization to the *HBG1* promoter is schematically depicted. (**G** and **H**) Relative enrichment of H3K27ac at the HS2 enhancer and downstream *HBG1* promoter 72 h post-transfection with 4 *HBG1*-targeting gRNAs and the indicated dCas9-based transcriptional activators is shown for HEK293T and HeLa cells, respectively (*n* = 3 independent replicates, error bars indicate ± SEM, * indicates adjusted *P*-value < 0.05 compared to dCas9 control-treated cells using two-tailed Student *t*-test and correcting for multiple comparisons and FDR using two-stage linear step-up procedure of Benjamini, Krieger and Yekutieli). (**I** and **J**) The data points from Figure 3G and H, respectively; are presented as scatter plots with Pearson correlation coefficients (*r*) and *P-*values (calculated using two-tailed Student *t*-test) indicated.

Given our observations that the transcriptional activity at the HS2 enhancer (i.e. eRNA expression) was correlated with *HBG1* mRNA output, we next investigated whether there was a reciprocal transcriptional relationship between HS2 and *HBG1*. We targeted dCas9, dCas9-VPR, dCas9-SAM, dCas9-SunTag, dCas9-p300 or dCas9-CBP to the *HBG1* promoter and then measured *HBG1* mRNA and resulting HS2 eRNAs using RT-qPCR (Figure 3A). As expected, in both HEK293T cells and HeLa cells, all dCas9-based transcriptional activators potently and significantly activated *HBG1* mRNA relative to dCas9 control treated samples (Figure 3B and C). Surprisingly, in most of these cases (9 out of 10 experiments; 90%) this targeted activation of *HBG1* mRNA by dCas9-based transcriptional activators also resulted in significant eRNA synthesis from the HS2 enhancer (Figure 3B and C). However, no positive correlation between activated *HBG1* mRNA levels and HS2 eRNA levels was observed in neither HEK293T nor HeLa cells (Pearson correlation coefficients = −0.040 and 0.026 and *P*-values = 0.89 and 0.93, respectively; Figure 3D and E, Supplemental Figures S15 and S16). Together, this data indicates that the synthetic transcriptional activation of the *HBG1* promoter can result in eRNA production from the upstream HS2 enhancer, but that targeted *HBG1* mRNA activation is not strongly correlated with increased HS2 eRNA output levels. We observed similar results when targeting the *HBG1* promoter in U2OS cells (Supplementary Figure S12) and when targeting the *KLK3* promoter in HEK293T and HeLa cells (Supplementary Figure S13).

### Relative increases in H3K27ac enrichment at the *HBG1* promoter are correlated with relative increases in H3K27ac at the HS2 enhancer

To further examine the regulatory reciprocity between the *HBG1* promoter and HS2 enhancer in human cells, we measured H3K27ac enrichment using CUT&RUN at each locus after targeting dCas9, dCas9-VPR, dCas9-SAM, dCas9-SunTag, dCas9-p300 or dCas9-CBP to the *HBG1* promoter in both HEK293T and HeLa cells (Figure 3F). Targeting dCas9-SAM, dCas9-SunTag, dCas9-p300, or dCas9-CBP to the *HBG1* promoter resulted in measurable enrichment of H3K27ac relative to dCas9 control-treated cells in HEK293T cells, however this enrichment was not statistically significant after correcting for the false discovery rate (Figure 3G). We attribute the lack of statistical significance to the high variance in these experiments. In HeLa cells, all dCas9-based transcriptional induced significant H3K27ac deposition when targeted to the *HBG1* promoter (Figure 3H). Notably, we also observed that even in the absence of increased levels of measured H3K27ac at the *HBG1* promoter, high levels of *HBG1* mRNA could nonetheless be activated (e.g. dCas9-VPR in HEK293T cells; compare Figure 3B and G). These data indicate that dCas9-based transcriptional activators (i.e. dCas9-VPR) can stimulate gene expression when targeted to an endogenous promoter without measurable increases in H3K27ac at targeted sites.

Interestingly, we also found significant enrichment of H3K27ac at the HS2 enhancer when targeting most (7 out of 10 experiments; 70%) dCas9-based transcriptional activators to the *HBG1* promoter (Figure 3G and H). In some cases, even though a particular dCas9-based transcriptional activator did not induce significant increases in H3K27ac at the *HBG1* promoter, significant H3K27ac enrichment was nevertheless observed at the HS2 enhancer (Figure 3G). Regardless of whether this was driven by technical or biological factors, the relative enrichment of H3K27ac measured at the *HBG1* promoter across all conditions was positively correlated with increases in measured H3K27ac at the HS2 enhancer in both HEK293T and HeLa cells (Pearson correlation coefficients = 0.92 and 0.82; *P*-values = 8.5e−7 and 2.0e−4, respectively; Figure 3I and J). These data reinforce the finding at the HS2 enhancer (Figure 2G and H) that dCas9-based transcriptional activators can deposit H3K27ac at endogenous loci, but that this deposition is governed by effector- and cell type-specific nuances. Finally, these results demonstrate that targeted increases in H3K27ac at the *HBG1* promoter can be transmitted in a positively correlated manner upstream to the HS2 enhancer.

### Increases in H3K27ac and transcription can spread between the HS2 enhancer and *HBG1* promoter when either regulatory element is targeted by dCas9-based transcriptional activators

**Figure 4.**
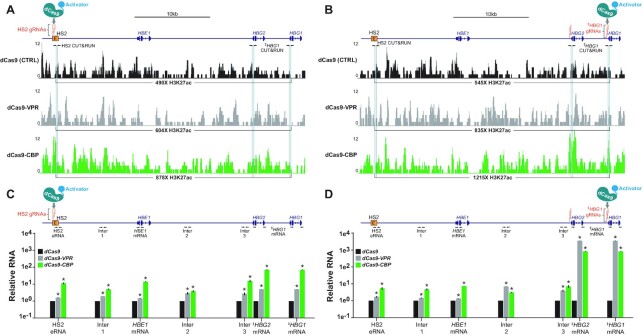
Increases in H3K27ac and transcription can spread between the HS2 enhancer and *HBG1* promoter when either regulatory element is targeted by dCas9-based transcriptional activators. (**A** and **B**) Genomic coordinates on human chromosome 11 spanning ∼5 282 828 bp to ∼5 247 660 bp (GRCh38/hg38) are shown along with H3K27ac CUT&RUN-seq enrichment data 72 h post-transfection with the indicated dCas9-based transcriptional activators and 4 HS2-targeting gRNAs (panel **A**) or 4 *HBG1*-targeting gRNAs (panel **B**) in HEK293T cells. Light blue bars indicate target sites for H3K27ac CUT&RUN qPCR in Figures 2 and 3. Numbers below respective tracks indicate total read coverage over indicated ranges. (**C** and **D**) Genomic coordinates on human chromosome 11 spanning ∼5 282 828 bp to ∼5 247 660 bp (GRCh38/hg38) are shown along with the relative expression of enhancer RNAs (eRNAs), intergenic ncRNAs, and mRNAs between the HS2 enhancer and *HBG1* promoter 72 h post-transfection with the indicated dCas9-based transcriptional activators and 4 HS2-targeting gRNAs (panel **C**) or 4 *HBG1*-targeting gRNAs (panel **D**) in HEK293T cells. Target locations of RT-qPCR primers are indicated in corresponding locations on chromosome 11. *n* = 3 independent replicates, error bars indicate − SEM, * indicates adjusted *P*-value <0.05 compared to dCas9 control-treated cells using two-tailed Student *t*-test and correcting for multiple comparisons and false discovery rate (FDR) using two-stage linear step-up procedure of Benjamini, Krieger and Yekutieli. ‡ serves to note that given the highly homologous genomic regions shared by *HBG1* and *HBG2*, gRNAs and RT-qPCR primers targeting *HBG1* also target *HBG2*.

To better understand how H3K27ac and transcription were communicated between the HS2 enhancer and *HBG1* promoter, we first performed CUT&RUN followed by next-generation sequencing (CUT&RUN-Seq) after targeting either regulatory element with either dCas9, dCas9-VPR or dCas9-CBP (Figure 4A and B). Targeting dCas9-VPR or dCas9-CBP to the HS2 enhancer resulted in measurable increases in H3K27ac across the ∼30 kb region between and including HS2 and *HBG1* relative to cells in which dCas9 was targeted to HS2 (604, 878 and 490 total reads for dCas9-VPR, dCas9-CBP and dCas9, respectively; Figure 4A, Supplemental Table S5). Similarly, targeting dCas9-VPR or dCas9-CBP to the *HBG1* promoter resulted in increased H3K27ac across this ∼30 kb region relative to cells in which dCas9 was targeted to *HBG1* (835, 1215 and 545 total reads for dCas9-VPR, dCas9-CBP and dCas9, respectively; Figure 4B, Supplemental Table S5). These data suggest that H3K27ac can measurably spread between the ∼30 kb region separating the HS2 enhancer and *HBG1* promoter after either locus has been synthetically transactivated by dCas9-CBP, and to a lesser extent, by dCas9-VPR. However, it should be noted that dCas9-CBP resulted in significant changes in H3K27ac enrichment across the HEK293T genome (Supplemental Figure S17).

Increases in noncoding transcription between the HS2 enhancer and *HBG1* promoter have also been observed in cell types where the β-globin locus is highly active (52,78). Therefore, we also used RT-qPCR to measure transcriptional output at selected locations within the ∼30kb region separating the HS2 enhancer and *HBG1* promoter after either locus was targeted by dCas9, dCas9-VPR, or dCas9-CBP (Figure 4C and D). Targeting dCas9-VPR or dCas9-CBP to either the HS2 enhancer or the *HBG1* promoter resulted in significant RNA expression across the ∼30 kb region spanning HS2 and *HBG1* relative to cells in which the HS2 or *HBG1* were targeted by dCas9 (Figure 4C and D). Increased RNA across this locus not only included noncoding transcripts, but interestingly increased *HBE1* mRNA expression was also observed, even when the *HBG1* promoter was targeted (Figure 4D). We detected similar increases in RNA across this locus when targeting dCas9-CBP to HS2 or *HBG1* in HeLa cells (Supplemental Figure S18). Together these data strongly suggest that targeted transactivation of either the HS2 enhancer or the *HBG1* promoter causes the spreading of transcriptional and epigenetic information across the ∼30 kb region separating these regulatory elements.

### dCas9-based transcriptional activators can increase the relative physical contact between the HS2 enhancer and *HBG1* promoter when targeted to HS2

**Figure 5.**
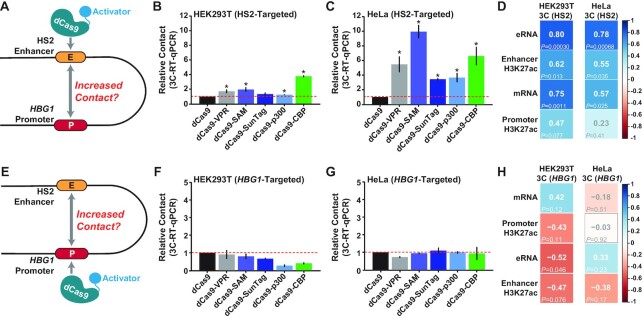
dCas9-based transcriptional activators can increase the physical contact frequency between the HS2 enhancer and *HBG1* promoter when targeted to HS2. (**A**) The targeting and 3C assay strategy for dCas9-based transcriptional activators after localization to the HS2 enhancer is schematically depicted. (**B** and **C**) Relative contact (using 3C RT-qPCR) between the HS2 enhancer and downstream *HBG1* promoter 72 h post-transfection with 4 HS2-targeting gRNAs and the indicated dCas9-based transcriptional activators is shown for HEK293T and HeLa cells, respectively (*n* = 3 independent replicates, error bars indicate ± SEM, * indicates adjusted *P*-value <0.05 compared to dCas9 control-treated cells using two-tailed Student *t*-test and correcting for multiple comparisons and false discovery rate (FDR) using two-stage linear step-up procedure of Benjamini, Krieger and Yekutieli). (**D**) The Pearson correlation coefficients (*r*) between 3C data points from Figure 5B and C, respectively; and other indicated data are shown along with corresponding *P-*values (calculated using two-tailed Student *t*-test). (**E**) The targeting and 3C assay strategy for dCas9-based transcriptional activators after localization to the *HBG1* promoter is schematically depicted. (**F** and **G**) Relative contact (using 3C RT-qPCR) between the HS2 enhancer and downstream *HBG1* promoter 72 h post-transfection with 4 *HBG1*-targeting gRNAs and the indicated dCas9-based transcriptional activators is shown for HEK293T and HeLa cells, respectively (*n* = 3 independent replicates, error bars indicate ± SEM, * indicates adjusted *P*-value <0.05 compared to dCas9 control-treated cells using two-tailed Student *t*-test and correcting for multiple comparisons and FDR using two-stage linear step-up procedure of Benjamini, Krieger and Yekutieli). (**H**) The Pearson correlation coefficients (*r*) between 3C data points from (F) and (G), respectively; and other indicated data are shown along with corresponding *P-*values (calculated using two-tailed Student *t*-test).

Physical interactions between enhancers and promoters have been shown to regulate gene expression outputs from promoters (49,79,80). For instance, forced chromatin looping at the β-globin locus using synthetic systems has been observed to induce the expression of downstream hemoglobin genes (56,81). Further, our results above and prior work (16,28,69) shows that targeting dCas9-based transcriptional activators to the HS2 enhancer can result in increased mRNA expression from the *HBG1* promoter. To more completely understand how changes in transcriptional regulation at either HS2 or *HBG1* are connected to physical contacts between the HS2 enhancer and *HBG1* promoter, we targeted dCas9-based transcriptional activators to the β-globin locus in HEK293T and HeLa cells (Figure 5A and E) and then used chromosome conformation capture (3C) to evaluate subsequent changes in contact frequency.

Although we observed low basal contact between the HS2 enhancer and *HBG1* promoter in HEK293T, and to a lesser extent in HeLa cells (Supplemental Figure S19), targeting the HS2 enhancer with either dCas9-VPR, dCas9-SAM, dCas9-p300 or dCas9-CBP resulted in significant increases in the relative contact frequency between the HS2 enhancer and *HBG1* promoter in HEK293T cells relative to dCas9 control (Figure 5B). Concordant results were obtained using ChIP-qPCR to detect dCas9-based transcriptional activators at *HBG1* when dCas9-VPR or dCas9-CBP were targeted to the HS2 enhancer in HEK293T cells (Supplemental Figure S20a). Significant increases in the relative contact frequency between these regulatory elements were also observed in HeLa cells when any dCas9-based transcriptional activator was targeted to HS2 relative to dCas9 control-treated cells (Figure 5C). Comparing our collective data (Figure 5D, Supplemental Figure S21) revealed a significant positive correlation between increases in relative contact frequency between HS2 and *HBG1* and increased eRNA synthesis in both HEK293T and HeLa cells (Pearson correlation coefficients = 0.80 and 0.78, *P*-values = 3.0e−4 and 6.8e−4, respectively) when HS2 was targeted by dCas9-based transcriptional activators. Increased contact frequencies between HS2 and *HBG1* were also positively correlated with H3K27ac enrichment at the HS2 enhancer and the *HBG1* promoter, as well as with *HBG1* mRNA expression (Figure 5D) which is expected given the positive correlations observed between HS2 eRNA synthesis, H3K27ac, and *HBG1* mRNA, when HS2 was targeted by dCas9-based transcriptional activators (Figure 2; Supplemental Figure S21). Together, these data demonstrate that dCas9-based transcriptional activators can increase the relative contact frequency between a targeted enhancer (HS2) and a corresponding distal, non-targeted promoter (*HBG1*).

Since we observed transcriptional and epigenetic (i.e. H3K27ac) changes at the HS2 enhancer when dCas9-based transcription activators were targeted to the *HBG1* promoter (Figures 3 and 4), we also used 3C to evaluate whether contact frequencies between HS2 and *HBG1* were increased when dCas9-based transcription activators were targeted to *HBG1* (Figure 5E). Interestingly, no significant increases in contact frequency between HS2 and *HBG1* were observed in any condition tested in either HEK293T or HeLa cells when dCas9-based transcription activators were targeted to *HBG1* (Figure 5F and G) and hence, no significant correlations were observed between *HBG1*:HS2 contact frequency and transcriptional outputs (i.e. eRNAs or mRNAs) nor H3K27ac levels when dCas9-based transcriptional activators were targeted to *HBG1* (Figure 5H). We also observed no enrichment of dCas9-based transcriptional activators using ChIP-qPCR at the HS2 enhancer when the *HBG1* promoter was targeted (Supplemental Figure S20b). These data suggest that increased contact frequency between HS2 and *HBG1* is likely driven by transcriptional activity that originates at HS2.

## DISCUSSION

Here, we systematically compared the relative potencies and expression levels of the most widely used CRISPR/Cas9-based transcriptional activators (and one dCas9-based transcriptional activator consisting of dCas9 fused to the HAT core of human CBP; dCas9-CBP) at different enhancer–promoter pairs in different human cell lines. We also used these CRISPR/Cas9-based transcriptional activators *in situ* within native chromatin to dissect how transcription, histone acetylation and physical contacts unite to coordinate the complex activity of the HS2-*HBG1* enhancer–promoter pair within the human β-globin locus. Collectively our studies provide new insights into the use of dCas9-based transcriptional activators and clarify the regulatory mechanisms that govern enhancer-mediated control of human gene expression.

For instance, our results show that the efficacy of dCas9-based transcriptional activators is influenced by both targeted locus and cell type (Figure 1), in agreement with previous observations (12–14,16,25,28). We also demonstrate that different dCas9-based transcriptional activators can display variable expression in different human cells (Figure 1, Supplemental Figures S6 and S8). These inconsistencies indicate that selecting the most effective dCas9-based transcriptional activator for a particular genomic target/cell type still requires some upfront validation. However, our results show that if the experimental goal is to maximize mRNA expression, targeting human promoters with dCas9-based transcriptional activators is generally superior to targeting cognate enhancers, at least in pooled gRNA contexts as tested here (Supplemental Table S4). Our data also demonstrate that in some cases, increased H3K27ac is not necessary for synthetic gene activation using dCas9-based transcriptional activators. For example, dCas9-VPR can significantly activate HS2 eRNAs, and consequently distal *HBG1* mRNA (Figure 2B), without significant increases in local H3K27ac (Figure 2G).

We also find that nearly all dCas9-based transcriptional activators tested here can induce the synthesis of eRNAs when targeted to the testbed HS2 enhancer (Figure 2B and C), as expected based on previous work (15,42). One notable exception here is the dCas9-SunTag system, which activated eRNAs to measurable but not statistically significant levels when targeted to HS2. This is possibly due to potential nuances associated with the recruitment transcriptional activators with the GCN4 scaffold, experimental variance, and/or cell-type specific subtleties—the latter seeming quite plausible as dCas9-SunTag did significantly activate eRNAs when targeted to HS2 in U2OS cells (Supplemental Figure S12). Regardless, all dCas9-based transcriptional activators significantly induced *HBG1* mRNA when targeted to HS2. In fact, our results quantitatively demonstrate that the activation of eRNAs from HS2 using dCas9-based transcriptional activators is strongly correlated to increases in downstream *HBG1* gene expression (Figure 2D and E, Supplemental Figures S11 and S12) consistent with previous observations and indicating that eRNA induction is intrinsically connected to the activation of downstream cognate promoters (40,42). Surprisingly, we also found that using dCas9-based transcriptional activators to induce *HBG1* mRNA resulted in robust eRNA expression from HS2 (Figure 3B and C, Supplemental Figure S12). Taken together these data suggest that the recruitment of transcriptional machinery to either HS2 or *HBG1* can reciprocally transactivate both loci. However, the transcriptional coordination from enhancer to promoter is positively correlated, while the coordination from promoter to enhancer appears to be more generalized (Figures 2D, E, 3D, and E). While this reciprocal coordination is almost certainly not universal among all human enhancer–promoter pairs, we also observed similar mechanistic patterns at the human *KLK3* locus (Supplementary Figure S13).

The data here also show that in addition to transcriptional reciprocity, epigenetic reciprocity exists between the HS2 enhancer and *HBG1* promoter. Specifically, H3K27ac deposited at the HS2 enhancer by dCas9-based transcriptional activators can be transmitted downstream to the *HBG1* promoter (Figures 2G, H and 4A), and conversely, H3K27ac synthetically deposited at the *HBG1* promoter can be transmitted upstream to the HS2 enhancer (Figures 3G, H and 4B). We attribute this transmission to the spreading of H3K27ac between the HS2 and *HBG1* loci because when dCas9-based transcriptional activators were targeted to either regulatory element, increased levels of H3K27ac were observed throughout the intervening genomic sequence (Figure 4A and B). One limitation of this analysis is that dCas9-CBP resulted in significant genome-wide differences in H3K27ac when targeted to either HS2 or *HBG1* (Supplemental Figure S17). However, we also observed significant spreading of transcriptional activation across the genomic region spanning HS2 and *HBG1* when either locus was targeted by dCas9-VPR or dCas9-CBP (Figure 4C and D), and together these data support a mechanism in which transcriptional and epigenetic information can spread between this enhancer–promoter pair. Interestingly, when dCas9-based transcriptional activators were targeted to *HBG1*, upstream *HBE*1 expression was also increased, highlighting the intimate and complex transcriptional relationships that exist between the genes within the human β-globin locus (52).

Our findings here using emerging synthetic dCas9-based transcriptional activators also indicate that the transcriptional and epigenetic activity at the HS2 enhancer serves to tailor the output of the *HBG1* promoter. Although basal physical contacts can likely occur between these two cognate regulatory loci in the absence of high levels of transcription (Supplemental Figures S9 and S19), contact frequencies can only be appreciably and significantly increased in HEK293T and HeLa cells when the HS2 enhancer is synthetically activated, but not when the *HBG1* locus is synthetically activated (Figure 5). Indeed, increased RNA synthesis, H3K27ac, and contact frequency at both loci can all be catalyzed by targeting HS2 but not *vice versa*. Furthermore, the high correlation between eRNA transcription and relative contact frequency between these loci suggests that the production of eRNAs, and hence transcriptional activity at an enhancer, can impact enhancer–promoter looping (82–84). Our collective correlative results (Supplemental Figure S15) are also consistent with recent observations that enhancer–promoter pairs display higher correlations between H3K27ac and genic transcription when looped versus when non-looped (85).

**Figure 6.**
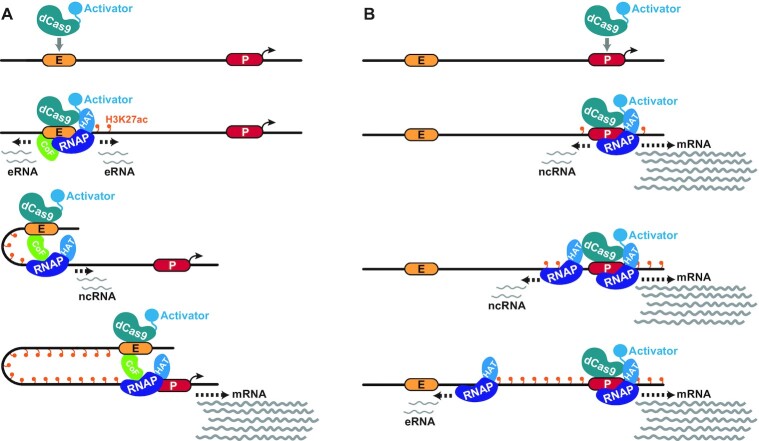
A model for transcriptional tracking that coordinates activity between HS2 and *HBG1* in response to dCas9-based transactivation. (**A**) Based on previous observations and our work here, when targeted to the HS2 enhancer dCas9-based transcriptional activators drive a cascade wherein RNA polymerase II (RNAP) in complex with associated cofactors (CoF, green) and possibly histone acetyltransferase (HAT) proteins drive the synthesis of HS2 eRNAs (likely bidirectionally). This complex then tracks toward *HBG1* while retaining HS2 and transcribing ncRNA at low levels (thin gray lines) that are coincident with increases in H3K27ac (orange lollipops) throughout the intervening genomic sequence. Once this complex and the HS2 enhancer physically engage the *HBG1* promoter, increased levels of *HBG1* mRNA (thick gray lines) are produced. (**B**) dCas9-based transcriptional activators can also elicit a tracking that originates from the *HBG1* promoter and results in the production of upstream RNAs—including HS2 eRNAs. Although the tracking from *HBG1* toward HS2 also occurs with coincident increases in H3K27ac (orange lollipops) throughout the intervening genomic sequence, it does not result in increased physical contacts between *HBG1* and HS2.

Altogether the data here most closely agree with a facilitated tracking and transcription mechanism that coordinates activity between HS2 and *HBG1* at the human β-globin locus (78,86). Based on previous observations and our work here, we suggest that when targeted to the HS2 enhancer, dCas9-based transcriptional activators trigger a cascade of activity wherein RNA polymerase II (RNAP) in complex with associated cofactors (which may be constitutively present at HS2) drive the synthesis of HS2 eRNAs, likely bidirectionally (Figure 6A). The RNAP/cofactor complex then tracks toward *HBG1* transcribing ncRNA at low levels throughout the intervening genomic sequence, while also maintaining physical contact with HS2. Once the *HBG1* promoter is engaged by this tracking complex, which ultimately results in increased contact frequency between HS2 and *HBG1* (Figure 5B and C), increased levels of *HBG1* mRNA are produced. The mechanisms surrounding how the RNAP/cofactor complex might adopt directionality, how *HBG1* mRNA expression is prioritized over other genes within the human β-globin locus, and the duration of functional physical contacts between HS2-*HBG1*, remain incompletely understood.

Surprisingly, our data also indicate that dCas9-based transcriptional activators can trigger a form of tracking and transcription that originates from the *HBG1* promoter and results in the production of upstream RNAs—including HS2 eRNAs and *HBE1* mRNAs. The increase in these upstream RNAs could be a byproduct of bidirectional transcription (73) that initiates from the *HBG1* promoter when it is targeted by dCas9-based transcriptional activators. In contrast to the tracking and transcription originating from HS2, the transcriptional tracking to HS2 that originates from *HBG1* occurs without coincident increases in physical contact between *HBG1* and HS2 (Figure 6B). Whether this lack of contact is due to different cofactor localization at *HBG1* versus HS2 or is genetically encoded, remains to be determined. Finally, the transmission of H3K27ac between these regulatory elements is perhaps the least clear from a mechanistic perspective. Emerging models hint at the interdependence between RNAP and H3K27ac and the more supportive role of H3K27ac in eukaryotic transcription (87,88). Therefore, it is plausible that the transcriptional tracking between HS2 and *HBG1* could itself influence the hyperacetylation observed at and between these loci when either regulatory element is naturally activated, or when synthetically activated by CRISPR-based technologies.

In sum, our studies here combine cutting-edge CRISPR-based epigenome editing technologies with a biomedically important and mechanistically valuable testbed locus to refine our understanding of how human enhancers can control the activity of corresponding genes. Although a greater diversity of epigenome editing tools and an expanded understanding of how chromatin state(s) and cell type-specific trans factors influence these phenomena are needed, our work here serves as a useful roadmap that highlights the utility of emerging dCas9-based epigenome editing technologies for reshaping the transcriptional and epigenomic activity of the endogenous human genome and dissecting human gene regulatory mechanisms.

## DATA AVAILABILITY

RT-qPCR data and full Western blots are available in the source data. All dCas9-based fusion variants are available through Addgene and all reagents are available from the authors upon request. All next generation sequencing data have been uploaded to the NCBI Gene Expression Omnibus repository under GEO accession number GSE205858.

## Supplementary Material

gkac582_Supplemental_File
