# Supplementary material for: Systematic comparison of CRISPR-based transcriptional activators uncovers gene-regulatory features of enhancer–promoter interactions
Source: Nucleic Acids Res. 2022 Jul 18;50(14):7842–55. doi: 10.1093/nar/gkac582 (PMC9371918; doi:10.1093/nar/gkac582)
Supplement: gkac582_Supplemental_File [file gkac582_supplemental_file.pdf]

Supplementary Table 1. Cloning primers.

Name	Primer sequence (5’ to 3’)
FLAG insert for Addgene 61423 (MS2-P65-HSF1)	ACCCCACTGTCTCCGCTAGCGATTACAAAGACGATGACGATAAGGCTAGCGGCAGTGGAGAGGG
FLAG insert for Addgene 61425 (dCas9-VP64)	ACCTGGACATGCTGATTAACTGTACAGATTACAAAGACGATGACGATAAGTGTACAGGCAGTGGAGAGGGCAGAGG
FLAG insert for Addgene 60903 (dCas9-10xGCN4)	CCGGTCCGATGGATCTACAGCGGCCGGATTACAAAGACGATGACGATAAGCGGCCGCAAGGTGGAGGTGGACCCAA
FLAG insert for Addgene 60904 (scFv-GCN4-sfGFP-VP64-GB1)	GTAGCGGTGGTGGTACTAGTGATTACAAAGACGATGACGATAAGACTAGTCCAAAAACAAGGAG
FLAG insert forward for Addgene 63798 (dCas9-VPR)	GCTAAGAAGAAGAAAGGATCCTCGCCAGGGATCCGT
FLAG insert reverse for Addgene 63798 (dCas9-VPR)	AGTTTGTTGCGCCGGATCCCTTATCGTCATCGTCTTTGTAATCGGATCCAAACAGAGATGTGTCGAAGAT

Supplementary Table 2. gRNA information.

Target Location	Protospacer Sequence (5'-3')	Genomic Location (GRCh38/hg38 Assembly)	Reference
MYOD promoter_A	CCTGGGCTCCGGGGCGTTT	chr11:17719509-17719527	Perez-Pinera et al., Nat Methods. 2013
MYOD promoter_B	GGCCCCTGCGGCCACCCCG	chr11:17719422-17719439	Perez-Pinera et al., Nat Methods. 2013
MYOD promoter_C	CTCCCTCCCTGCCCGGTAG	chr11:17719350-17719368	Perez-Pinera et al., Nat Methods. 2013
MYOD promoter_D	AGGTTTGGAAAGGGCGTGC	chr11:17719290-17719308	Perez-Pinera et al., Nat Methods. 2013
OCT4 promoter_A	ACTCCACTGCACTCCAGTCT	chr6:31,170934-31170953	Hu et al., Nucleic Acids Research. 2014
OCT4 promoter_B	TCTGTGGGGGACCTGCACTG	chr6:31170866-31170885	Hu et al., Nucleic Acids Research. 2014
OCT4 promoter_C	GGGGCGCCAGTTGTGTCTCC	chr6:31,170,836-31,170,855	Hu et al., Nucleic Acids Research. 2014
OCT4 promoter_D	ACACCATTGCCACCACCATT	chr6:31,170,797-31,170,816	Hu et al., Nucleic Acids Research. 2014
HBG1 promoter_A	GCTAGGGATGAAGAATAAA	chr11:5,249,883-5,249,901	Perez-Pinera et al., Nat Methods. 2013
HBG1 promoter_B	TTGACCAATAGCCTTGACA	chr11:5,249,958-5,249,976	Perez-Pinera et al., Nat Methods. 2013
HBG1 promoter_C	TGCAAATATCTGTCTGAAA	chr11:5,250,020-5,250,038	Perez-Pinera et al., Nat Methods. 2013
HBG1 promoter_D	AAATTAGCAGTATCCTCTT	chr11:5,250,066-5,250,084	Perez-Pinera et al., Nat Methods. 2013
KLK3 promoter_A	CTGCAATTACTAGATCACCC	chr19:50854727-50854746	This Study
KLK3 promoter_B	GCTCATGGAGACTTCATCTA	chr19:50854688-50854706	This Study
KLK3 promoter_C	AAGGCAGAGGAGACACGCCC	chr19:50854662-50854680	This Study
MYOD DRR_A	TGTTTTCAGCTTCCAAACT	chr11:17736528-17736546	Hilton et al., Nat. Biotech. 2015
MYOD DRR_B	CATGAAGACAGCAGAAGCC	chr11:17736311-17736329	Hilton et al., Nat. Biotech. 2015
MYOD DRR_C	GGCCCACATTCCTTTCCAG	chr11:17736158-17736176	Hilton et al., Nat. Biotech. 2015
MYOD DRR_D	GGCTGGATTGGGTTTCCAG	chr11:17736065-17736083	Hilton et al., Nat. Biotech. 2015
OCT4 PE_A	AGTGATAAGACACCCGCTTT	chr6: 31171747 31171766	Hilton et al., Nat. Biotech. 2015
OCT4 PE_B	CAGACATCTAATACCACGGT	chr6: 31171827 31171846	Hilton et al., Nat. Biotech. 2015
OCT4 PE_C	AGGGAGAACGGGGCCTACCG	chr6: 31171843 31171862	Hilton et al., Nat. Biotech. 2015
OCT4 PE_D	ACTTCAGGTTCAAAGAAGCC	chr6: 31171948-31171967	Hilton et al., Nat. Biotech. 2015
HS2 Enhancer_A	AATATGTCACATTCTGTCTC	chr11:5280570-5280588	Hilton et al., Nat. Biotech. 2015
HS2 Enhancer_B	GGACTATGGGAGGTCACTAA	chr11:5280878-5280897	Hilton et al., Nat. Biotech. 2015
HS2 Enhnacer_C	GAAGGTTACACAGAACCAGA	chr11:5280803-5280822	Hilton et al., Nat. Biotech. 2015
HS2 Enhancer_D	GCCCTGTAAGCATCCTGCTG	chr11:5280668-5280687	Hilton et al., Nat. Biotech. 2015
KLK3 Enhancer_A	CTGGTCACCCTACAAGATTT	chr19:50850653-50850672	Mahata et al., bioRxiv 2022
KLK3 Enhancer_B	CTGGTGAGAAACCTGAGATT	chr19:50850713-50850732	Mahata et al., bioRxiv 2022
KLK3 Enhancer_C	GTTGTCCCAGTATAAGATTG	chr19:50850742-50850761	Mahata et al., bioRxiv 2022
KLK3 Enhancer_D	GATTGAAAACAGACCTACTC	chr19:50850816-50850835	Mahata et al., bioRxiv 2022

Supplementary Table 3. Quantitative reverse transcription PCR and ChIP-qPCR primers and conditions.

Target	Forward Primer (5'-3')	Reverse Primer (5'-3')	Cycling Parameters	
GAPDH	AATGACCCCTTCATTGACC	TTGATTTTGGAGGGATCTCG	95˚C 30 sec<br>95˚C 10 sec<br>60˚C 30 sec	49 x
MYOD	TCCCTCTTTCACGGTCTCAC	AACACCCGACTGCTGTATCC	95˚C 30 sec<br>95˚C 10 sec<br>60˚C 30 sec	49 x
OCT4	CGAAAGAGAAAGCGAACCAGTATCGAGAAC	CGTTGTGCATAGTCGCTGCTTGATCGC	95˚C 30 sec<br>95˚C 10 sec<br>60˚C 30 sec	49 x
HBG1	GCTGAGTGAACTGCACTGTGA	GAATTCTTTGCCGAAATGGA	95˚C 30 sec<br>95˚C 10 sec<br>60˚C 30 sec	49 x
HBE1	TCACTAGCAAGCTCTCAGGC	AACAACGAGGAGTCTGCCC	95˚C 30 sec<br>95˚C 10 sec<br>60˚C 30 sec	49 x
HS2 eRNA	TGCAGGTGGCCTCTATACCT	CCTTCCTCTTCCATATCCTTG	95˚C 30 sec<br>95˚C 10 sec<br>60˚C 30 sec	49 x
KLK3	AAGTGGATCAAGGACACCATC	AGTCTTGGCCTGGTCATTTC	95˚C 30 sec<br>95˚C 10 sec<br>60˚C 30 sec	49 x
KLK3 eRNA	AGGGTATCACCAGCCCTTCT	GAGGATGTCGGCAGCTCTAC	95˚C 30 sec<br>95˚C 10 sec<br>60˚C 30 sec	49 x
HBG1 CUT&RUN/ChIP	CTTCAGCAGTTCCACACACT	GTCCTTCCTTCCCTCCCT	95˚C 30 sec<br>95˚C 10 sec<br>60˚C 30 sec	49 x
HS2 CUT&RUN/ChIP	GCATGAGCAGTTCTGGCCAGGC	GGGGACCCAGATAGGAGTCATCAC	95˚C 30 sec<br>95˚C 10 sec<br>60˚C 30 sec	49 x
HS2-*HBG1* 3C_1	GGGAGGTCACTAATGGAGACA	TCTAGCCCCACAGGAGTTTG	95˚C 30 sec<br>95˚C 10 sec<br>60˚C 30 sec	49 x
HS2-*HBG1* 3C_2	GGGAGGTCACTAATGGAGACA	CCAGATAGTACAGGGCCTGG	95˚C 30 sec<br>95˚C 10 sec<br>60˚C 30 sec	49 x
HS2-*HBG1* 3C_3	GGGAGGTCACTAATGGAGACA	GAATATGGCCCTATCTTCAGCC	95˚C 30 sec<br>95˚C 10 sec<br>60˚C 30 sec	49 x
HS2-*HBG1* 3C_4	GGGAGGTCACTAATGGAGACA	CCAGTGACTAGTGCTGCAAG	95˚C 30 sec<br>95˚C 10 sec<br>60˚C 30 sec	49 x
HS2-*HBG1* 3C_5 (Fig. 5)	GGGAGGTCACTAATGGAGACA	CTGGCCTTTTAGCCATCTGT	95˚C 30 sec<br>95˚C 10 sec<br>60˚C 30 sec	49 x
HS2-*HBG1* 3C_6	GGGAGGTCACTAATGGAGACA	GGGCTGTGAAAAGCTAAGTGC	95˚C 30 sec<br>95˚C 10 sec<br>60˚C 30 sec	49 x
HS2-*HBG1* 3C_7	GGGAGGTCACTAATGGAGACA	ATGGTCAGAGCCTCAGTTTCAA	95˚C 30 sec<br>95˚C 10 sec<br>60˚C 30 sec	49 x
*GAPDH*_3C	ACAGTCCATGCCATCACTGCC	GCCTGCTTCACCACCTTCTTG	95˚C 30 sec<br>95˚C 10 sec<br>60˚C 30 sec	49 x
HS2-*HBG1*_inter1	GGCTGATTCTGGCTTTCTACTC	TAGTGTACCTCTGCCTCATCTC	95˚C 30 sec<br>95˚C 10 sec<br>60˚C 30 sec	49 x

HS2-<br>*HBG1*_inter2	GTCTGTCCTCTTTCAGACTCTTT	ACCACCATGGCACATCTATAC	95˚C 30 sec<br>95˚C 10 sec<br>60˚C 30 sec	49 x
HS2-<br>*HBG1*_inter3	GCCATGTTAAGTGGCCAATATG	GCTCGGTAAAGATGATGGTAGAA	95˚C 30 sec<br>95˚C 10 sec<br>60˚C 30 sec	49 x

Supplemental Table 4: mRNA activation from promoters vs. from enhancers.

Activators	Cell-line	Target gene	**average activation form promoter ± SEM**	**average activation form enhancer ± SEM**	*P*-value
dCas9-VPR	HEK 293T	*MYOD*	**1.48E02± 18.74**	**6.61 ± 0.84**	0.002
		OCT4	**1.65E03± 4.08E02**	**22.08 ± 4.40**	0.016
		HBG	**1.18E04 ± 3.79E3**	**26.73 ± 7.07**	0.036
	HeLa	*MYOD*	**2.69E04 ± 1.60E04**	**22.25 ± 8.73**	0.168
		OCT4	**1.03E03 ± 54.5**	**1.98 ± 0.32**	0.00005
		HBG	**3.15E05 ± 7.10E04**	**2.09E03 ± 4.22E02**	0.012
	K562	*MYOD*	**4.45E06 ± 2.12E06**	**9.17E03 ± 2.34E03**	0.104
		OCT4	**2.42E04 ± 1.41E03**	**3.96E03 ± 95.5**	0.0001
		HBG	**3.06 ± 1.18**	**4.14 ± 1.41**	0.590
dCas9-SAM	HEK 293T	*MYOD*	**9.93E02 ± 1.16E02**	**13.34 ± 1.84**	0.001
		OCT4	**9.91E02 ± 291.51**	**52.80 ± 4.86**	0.032
		HBG	**5.30E04 ± 1.52E04**	**15.78 ± 3.90**	0.025
	HeLa	*MYOD*	**5.50E03 ± 2.09E03**	**3.35E02 ± 60.51**	0.069
		OCT4	**1.28E02 ± 15.33**	**3.47 ± 0.81**	0.001
		HBG	**3.15E05 ± 6.29E04**	**4.11E04 ± 4.00E03**	0.012
	K562	*MYOD*	**9.85E04 ± 4.38E04**	**1.39E03 ± 8.96E02**	0.091
		OCT4	**1.12E02 ± 11.75**	**1.85E02 ± 3.38**	0.004
		HBG	**2.22 ± 0.63**	**1.87 ± 0.70**	0.730
dCas9-SunTag	HEK 293T	*MYOD*	**4.73E03 ± 18.84**	**2.31 ± 0.43**	0.00002
		OCT4	**3.75E02 ± 67.09**	**11.26 ± 1.21**	0.006
		HBG	**5.35E03 ± 1.33E03**	**5.46E02 ± 1.03E02**	0.023
	HeLa	*MYOD*	**5.33E03 ± 2.39E03**	**4.20 ± 2.22**	0.089
		OCT4	**19.52 ± 2.16**	**1.53 ± 0.47**	0.001
		HBG	**3.22E03 ± 1.01E03**	**8.37E03 ± 1.08E03**	0.025
	K562	*MYOD*	**5.34E03 ± 3.22E03**	**3.36E02 ± 1.90E02**	0.196
		OCT4	**1.29 ± 0.19**	**1.80 ± 0.22**	0.152
		HBG	**1.49 ± 0.42**	**2.27 ± 0.58**	0.335
dCas9-p300	HEK 293T	*MYOD*	**4.94E02 ± 1.10E02**	**4.08 ± 0.71**	0.011
		OCT4	**1.83E02 ± 39.93**	**58.79 ± 9.40**	0.039
		HBG	**6.24E03 ± 1.74E03**	**8.16E02 ± 1.4E02**	0.036
	HeLa	*MYOD*	**3.19E03 ± 1.45E03**	**1.66 ± 0.89**	0.093
		OCT4	**3.51 ± 0.14**	**1.77 ± 0.18**	0.001
		HBG	**2.06E03 ± 1.05E03**	**8.75 ± 2.28**	0.124
	K562	*MYOD*	**2.07E04 ± 8.73E03**	**1.51E02 ± 85.82**	0.078
		OCT4	**1.17E02 ± 48.93**	**5.73 ± 0.29**	0.086
		HBG	**1.43 ± 0.07**	**2.09 ± 0.63**	0.358
dCas9-CBP	HEK 293T	*MYOD*	**9.76E02 ± 1.18E02**	**4.31 ± 0.56**	0.001
		OCT4	**2.73E02 ± 1.82**	**77.00 ± 10.70**	0.0001
		HBG	**4.68E03 ± 8.37E02**	**2.69E03 ± 1.71E02**	0.081
	HeLa	*MYOD*	**3.87E03 ± 2.49E03**	**0.92 ± 0.31**	0.195
		OCT4	**7.08 ± 4.28**	**3.67 ± 0.32**	0.471
		HBG	**2.56E03 ± 6.34E02**	**67.49 ± 23.10**	0.017
	K562	*MYOD*	**5.16E04 ± 2.11E04**	**3.74E02 ± 48.67**	0.072
		OCT4	**160.02 ± 17.19**	**12.37 ± 0.47**	0.001
		HBG	**1.65 ± 0.32**	**1.92 ± 0.46**	0.655

Supplemental Table 5: H3K27ac CUT&RUN-Seq cumulative reads over the HS2-*HBG1* locus

Target Locus	Activator	**Cumulative H3K27ac CUT&RUN-Seq reads at HBG1/2 locus***	**Cumulative H3K27ac CUT&RUN-Seq reads at HS2 locus***	**Cumulative H3K27ac CUT&RUN-Seq reads between HS2 and HBG1/2**
HS2	dCas9	**1**	**9**	**490**
	dCas9-VPR	**1**	**0**	**604**
	dCas9-CBP	**17**	**31**	**878**
HBG1	dCas9	**7**	**1**	**545**
	dCas9-VPR	**11**	**0**	**835**
	dCas9-CBP	**91**	**3**	**1215**

*****Read counts for the *HBG1/2* and HS2 loci were calculated by counting all reads within the respective amplicons used for CUT&RUN-qPCR.

Supplemental Figure 1:

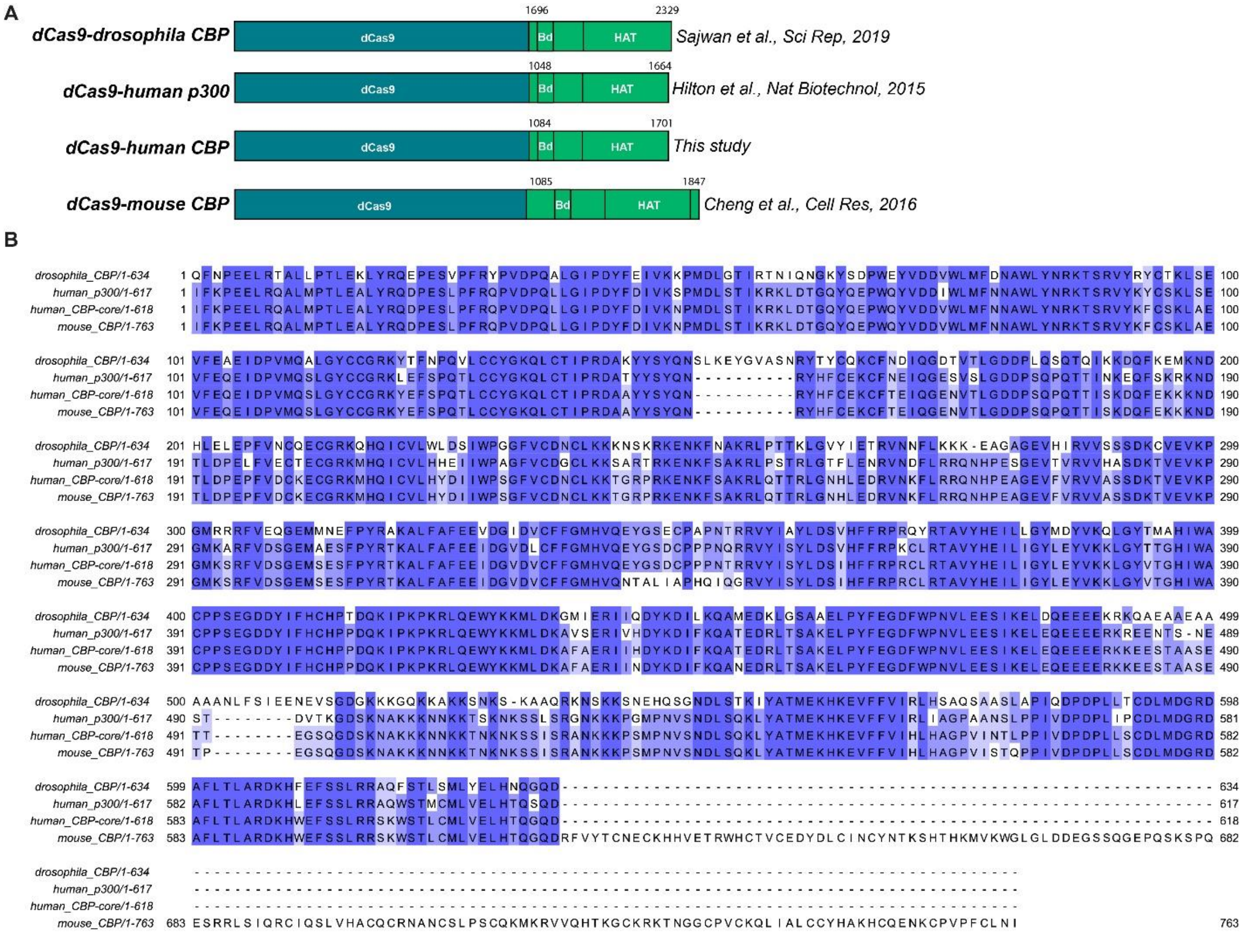

Supplemental Figure 1. Comparison of dCas9-CBP fusion proteins. A. Schematics of different dCas9-CBP fusion proteins and associated references are shown. **B.** Relative conservation among indicated proteins using Clustal Omega alignment is indicated. Darker blue indicates higher conservation.

Supplemental Figure 2:

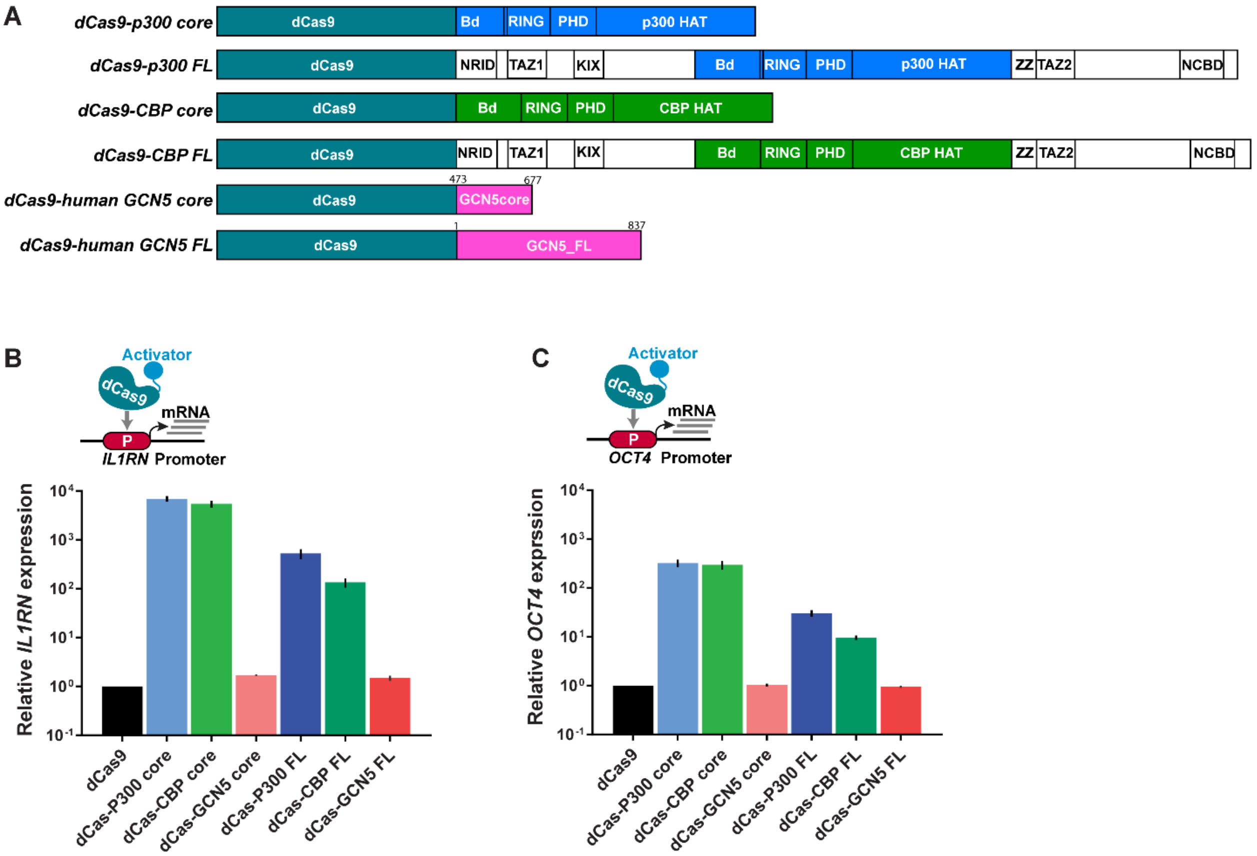

Supplemental Figure 2. Construction and comparative evaluation of full-length (FL) HAT protein and HAT "core" domain fusions to dCas9. A. Schematics of different dCas9-p300, dCas9-CBP, and dCas9-GCN5 fusion proteins. **B and C**. Relative activation of *IL1RN* or *OCT4*, respectively; when indicated fusion proteins are targeted to respective promoters using 4 gRNAs (n=3 independent replicates, error bars indicate +/- SEM).

Supplemental Figure 3:

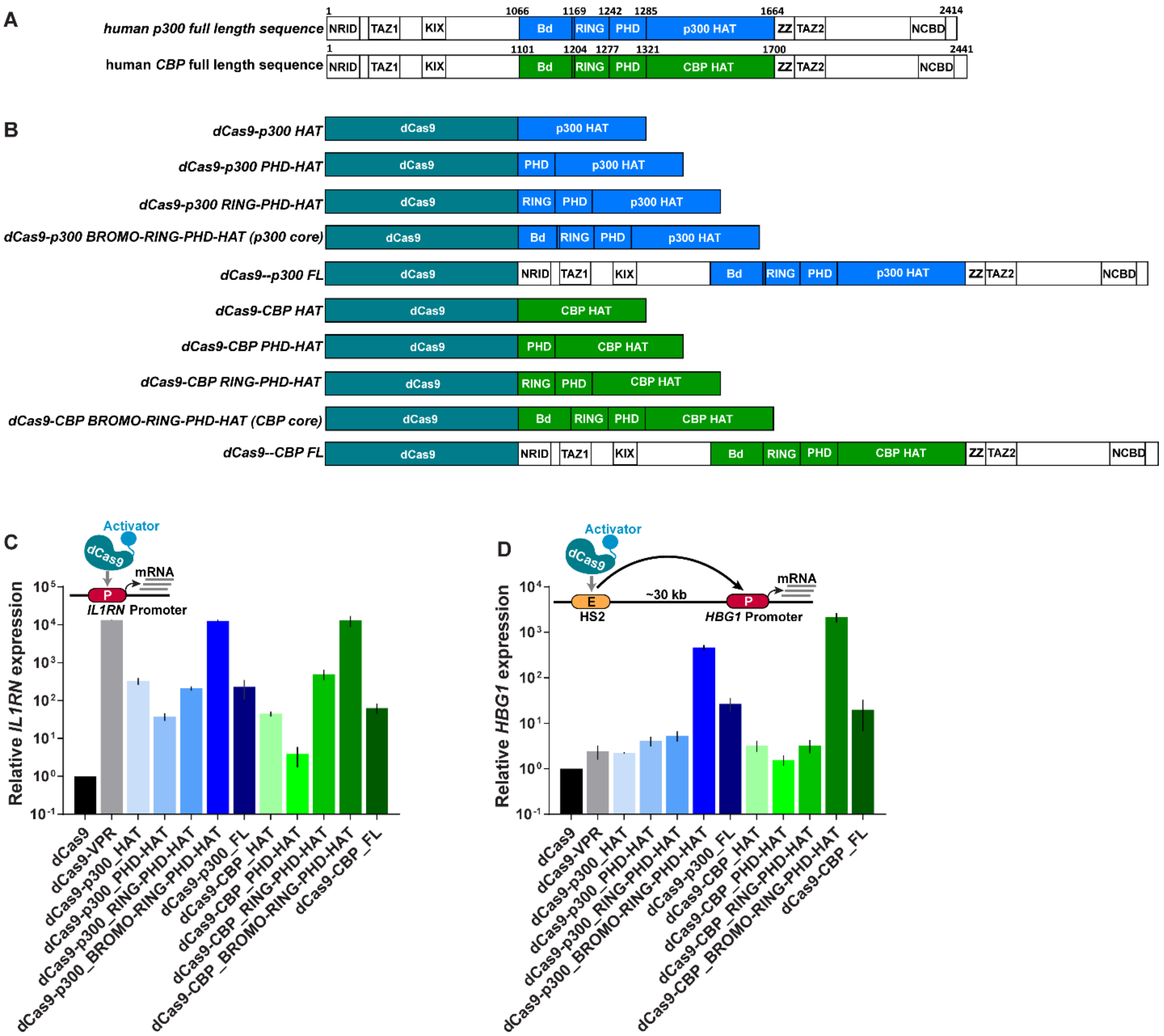

Supplemental Figure 3. Comparison of dCas9-HAT fusion proteins with different domain configurations. A. Schematics of the full-length (FL) human p300 and CBP proteins and selected domains are shown. **B**. Schematics of different dCas9-p300 and dCas9-CBP fusion protein variants. **C and D**. Relative activation of *IL1RN* or *OCT4*, respectively; when indicated fusion proteins are targeted to specified loci using 4 corresponding gRNAs (n=3 independent replicates for C, n=2 independent replicates for D, error bars indicate +/- SEM).

Supplemental Figure 4:

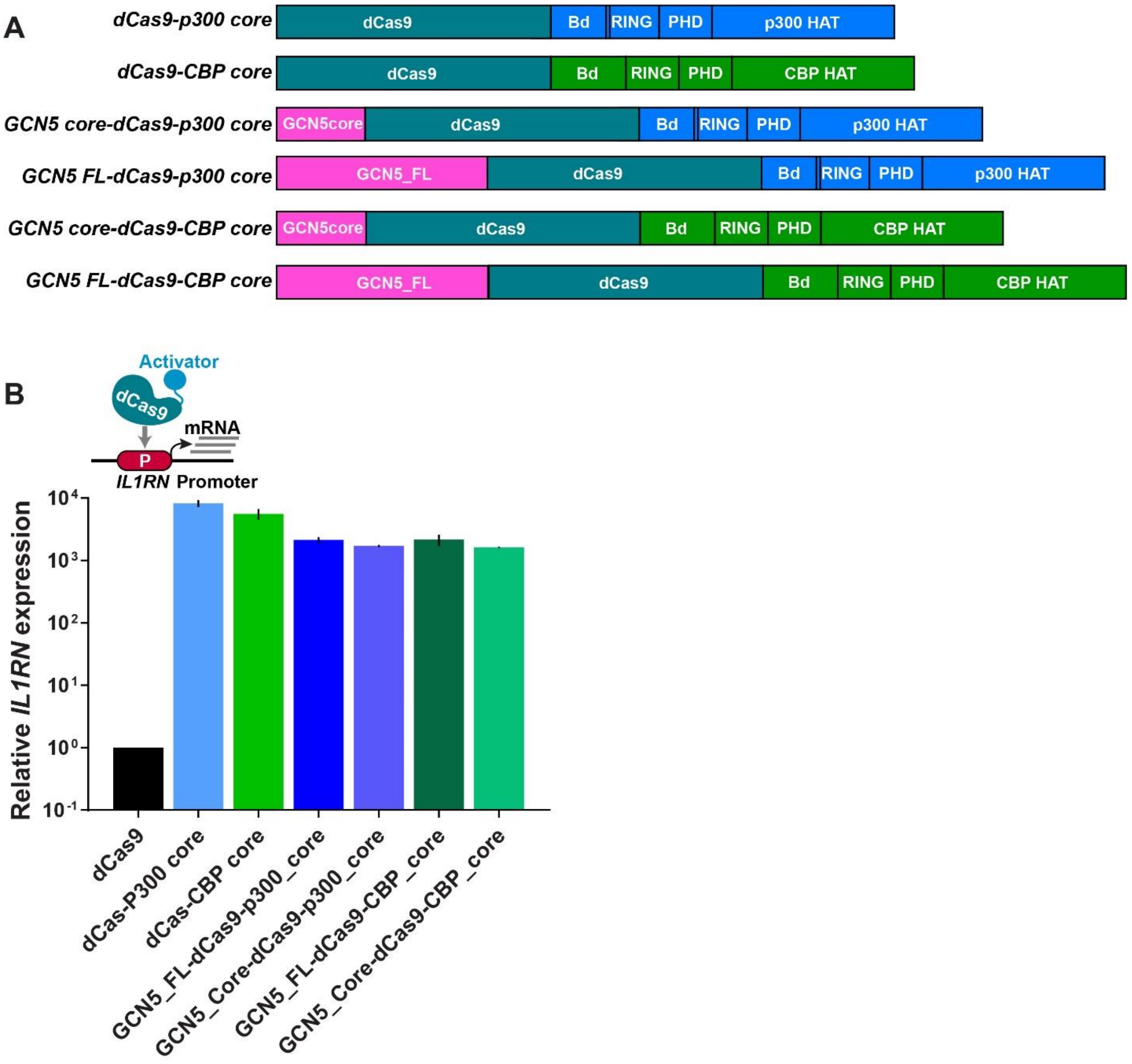

Supplemental Figure 4. Comparison of dCas9-p300 and dCas9-CBP fusion proteins harboring bipartite full length (FL) GCN5 or the GCN5 HAT core domain fusion. A. Schematics of the different dCas9-p300 and dCas9-CBP fusion protein variants. **B**. Relative activation of *IL1RN* when indicated fusion proteins are targeted to using 4 corresponding gRNAs (n=3 independent replicates, error bars indicate +/- SEM).

Supplemental Figure 5:

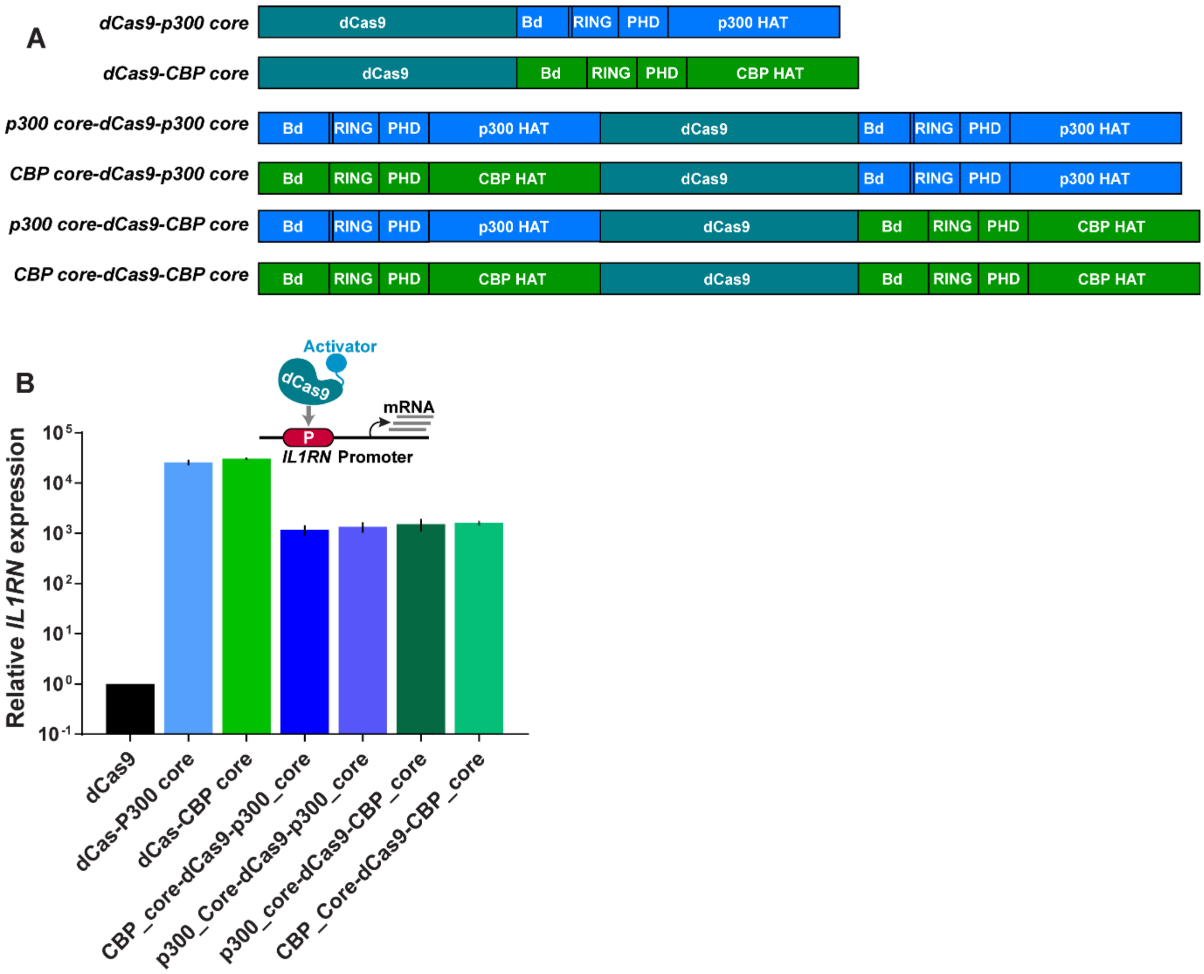

Supplemental Figure 5. Comparison of dCas9-p300 and dCas9-CBP fusion proteins harboring bipartite full length p300 or CBP HAT core domain fusion. A. Schematics of the different dCas9-p300 and dCas9-CBP fusion protein variants. **B**. Relative activation of *IL1RN* when indicated fusion proteins are targeted to using 4 corresponding gRNAs (n=3 independent replicates, error bars indicate +/- SEM).

Supplemental Figure 6:

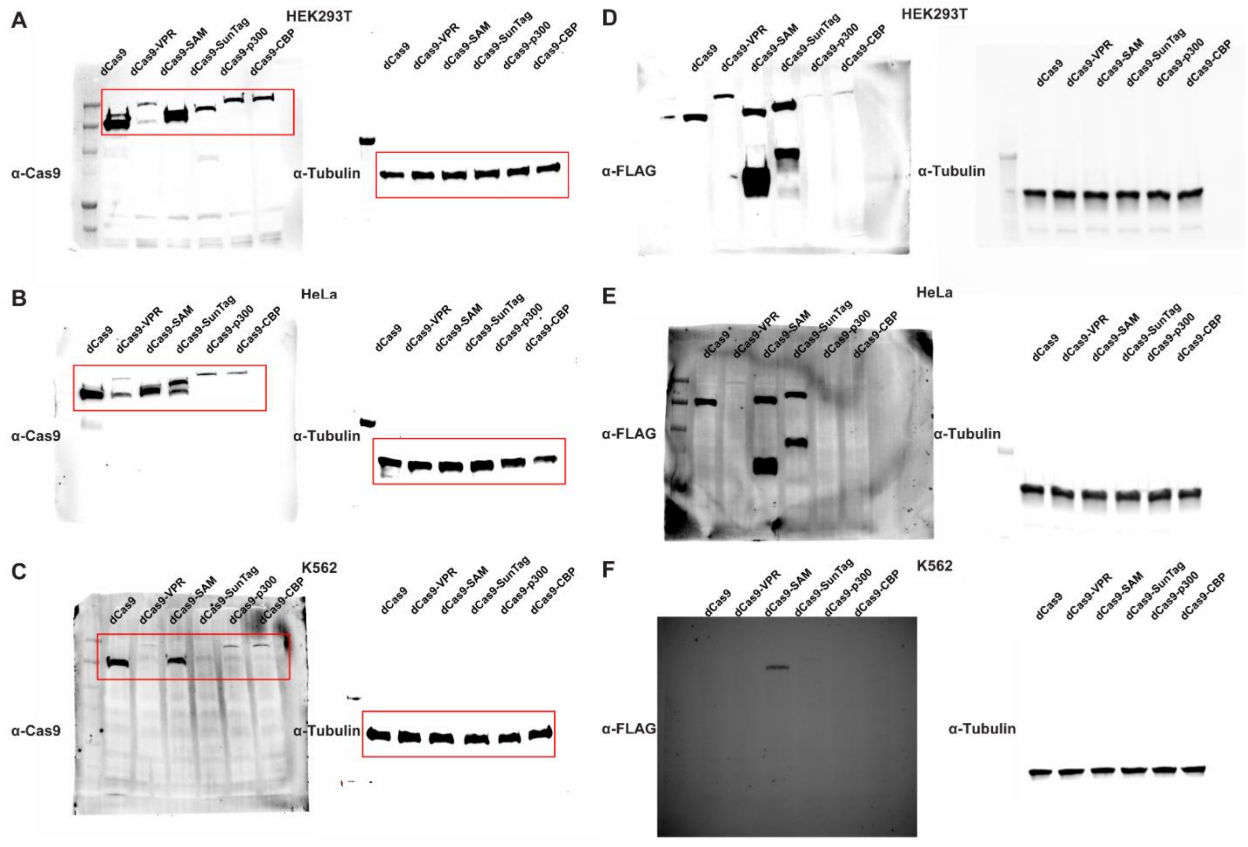

Supplemental Figure 6. Expression levels of dCas9-based transcriptional activation components in different cells. A-C. Expression of indicated dCas9-based transcriptional activation components in HEK293T, HeLa, and K562 cells, respectively; using an anti-Cas9 antibody and anti-tubulin loading control. **D-F**. Expression of indicated dCas9-based transcriptional activation components in HEK293T, HeLa, and K562 cells, respectively; using an anti-FLAG antibody and anti-tubulin loading control.

Supplemental Figure 7:

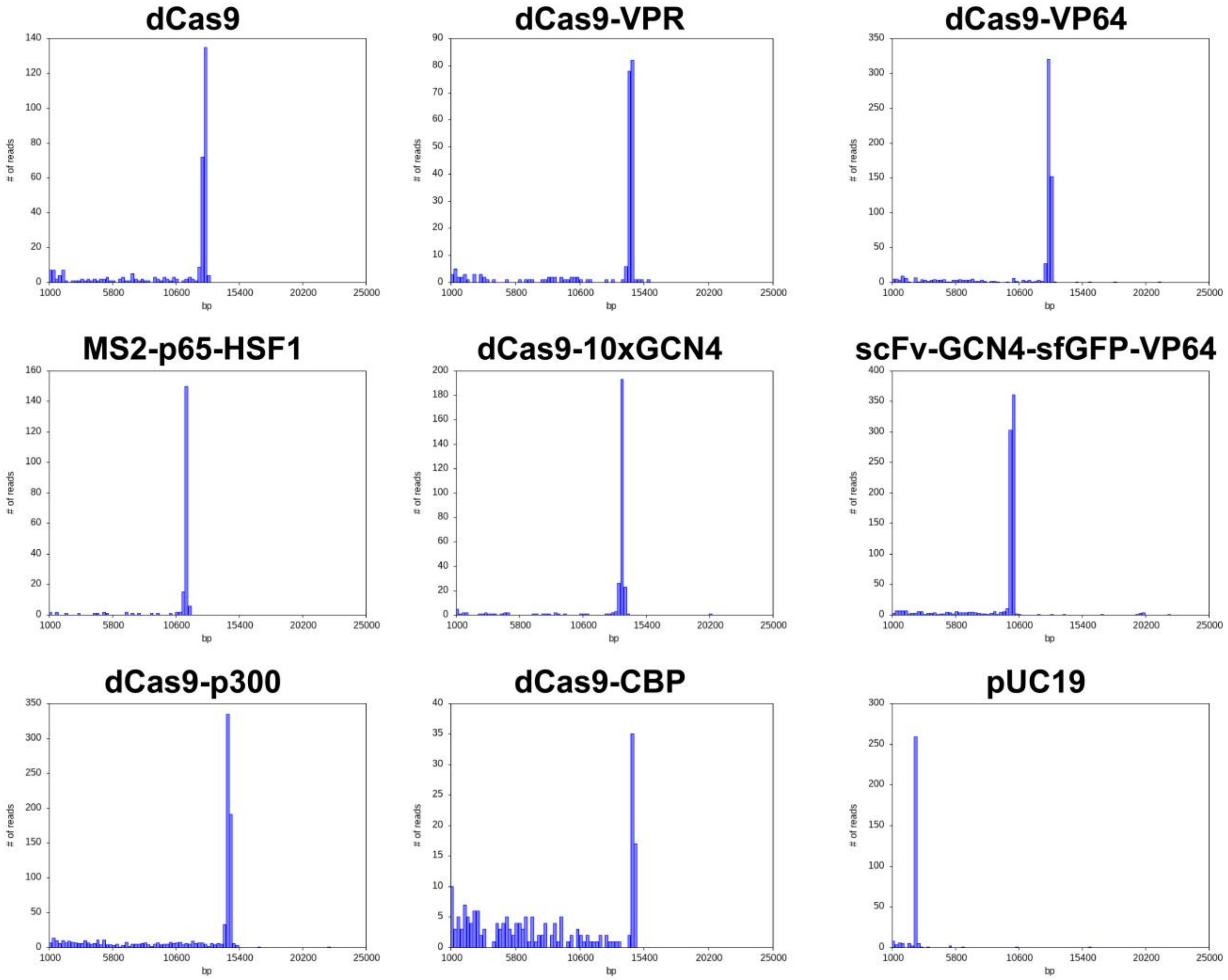

Supplemental Figure 7. Histograms of full-length plasmid sequences for all constructs used in this study. All plasmids above were sequenced by plasmidsaurus and confirmed to be clean preparations of single plasmids.

Supplemental Figure 8:

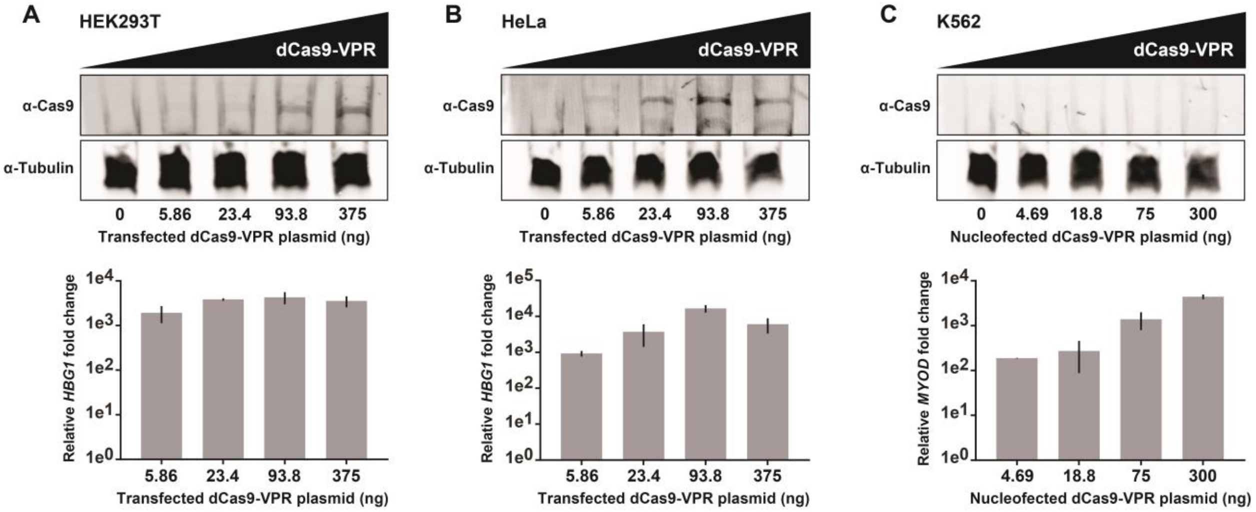

Supplemental Figure 8: Relationship between input dCas9-VPR plasmid, expression level, and transactivation potency in bulk HEK293T, HeLa, and K562 cells. A-C. Increasing amounts of dCas9-VPR plasmid along with corresponding gRNAs and filler plasmids were transfected into HEK293T cells (panel **A**), HeLa cells (panel **B**), or nucleofected into K562 cells (panel **C**). 72 hours post-transfection or nucleofection dCas9-VPR expression was probed using Western blot (top), and relative transactivation of gRNA-targeted promoters (*HBG1* for HEK293T and HeLa cells and *MYOD* for K562 cells) was measured using RT-qPCR (bottom).

Supplemental Figure 9:

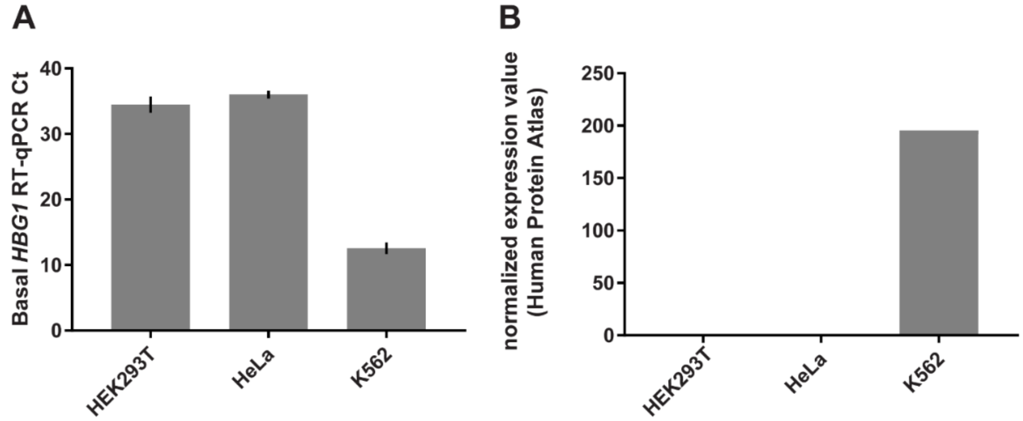

Supplemental Figure 9. Basal expression levels of *HBG1*. A. Expression of *HBG1*, in terms of raw cycle threshold (Ct) values in HEK293T, HeLa, and K562 cells, respectively (n= at least 3 independent replicates, error bars indicate +/- SEM). **B.** Normalized *HBG1* expression in HEK293T, HeLa, and K562 cells using data from the human protein atlas (https://www.proteinatlas.org).

Supplemental Figure 10:

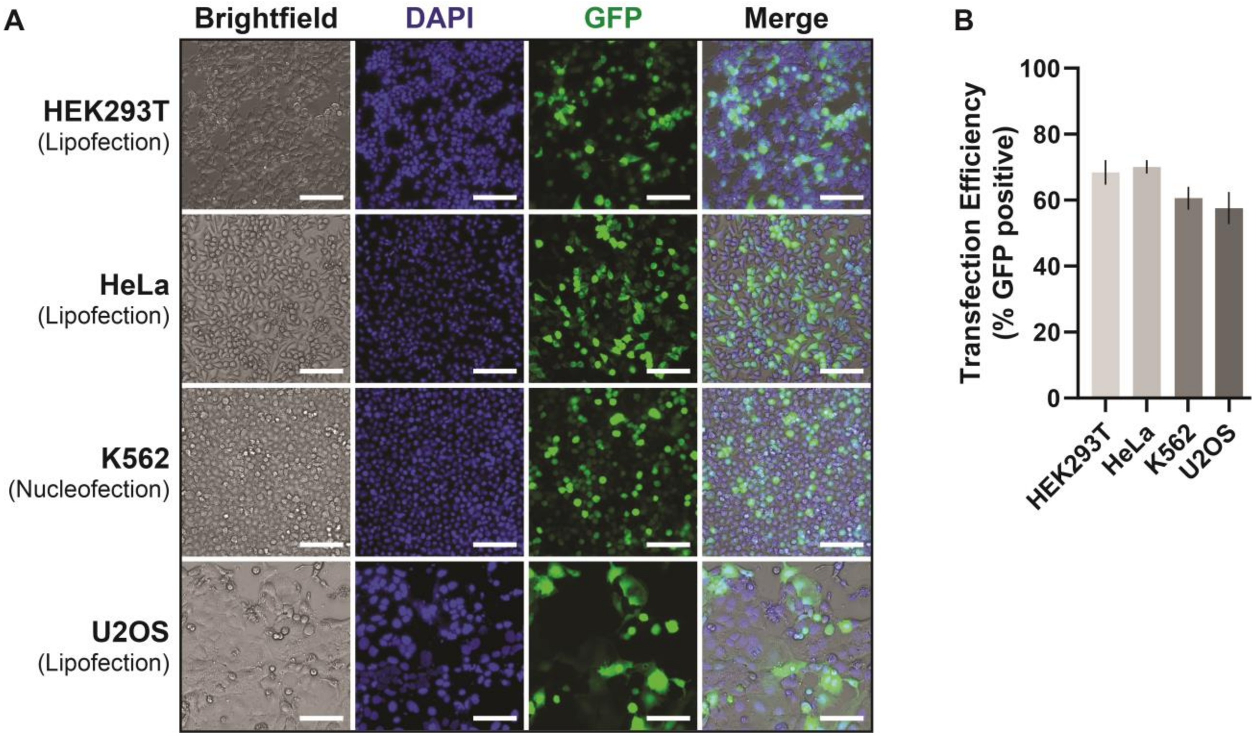

Supplemental Figure 10. Comparative transfection efficiencies among methods and cell lines used in this study. A. Brightfield, DAPI, GFP, and merged microscopy images after HEK293T, HeLa, K562, or U20S cells were transfected with pmaxGFP (Lonza, V4XC-2012) using either lipofection or nucleofection as indicated. 72 hours post-transfection cells were imaged at 20X magnification. Scale bars = 100μm. **B**. Transfection efficiency was measured as the ratio between the number of GFP positive cell nuclei and the total number of cell nuclei by the Nikon NIS Elements software.

Supplemental Figure 11:

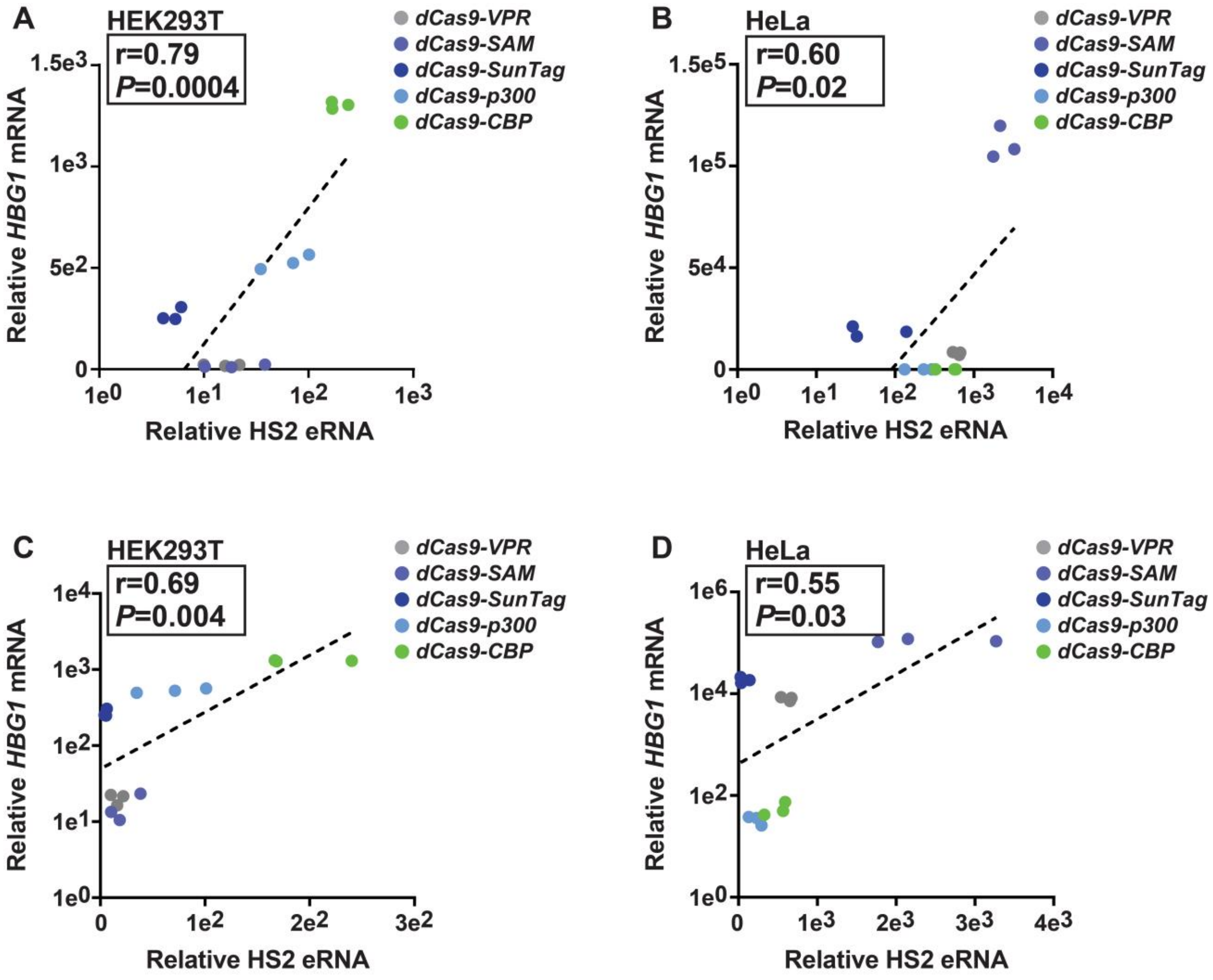

Supplemental Figure 11. *HBG1* mRNA and HS2 eRNA are significantly positively correlated when the HS2 enhancer is targeted by dCas9-based transcriptional activators. The data from main text Figures 2D (HEK293T cells) and 2E (HeLa cells), respectively is shown after being plotted and regressed in linear-log space (Panels **A** and **B**) and after being plotted and regressed in log-linear space (Panels **C** and **D**) to enhance clarity and evaluate variance.

Supplemental Figure 12:

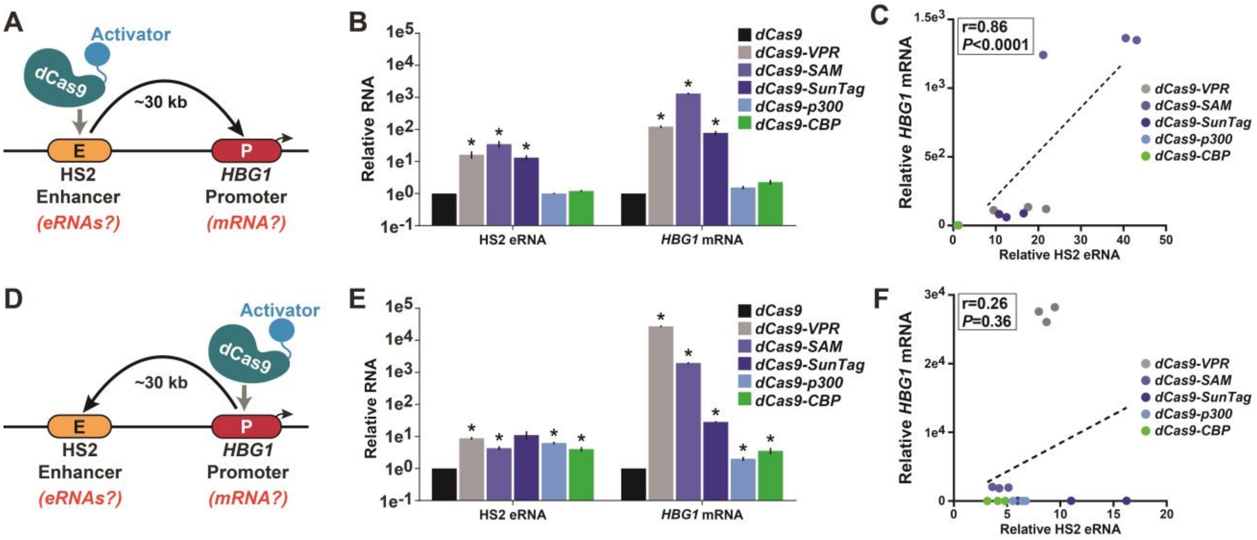

Supplemental Figure 12. dCas9-based transcriptional activators can induce RNA synthesis from both the *HBG1* promoter and the HS2 enhancer when targeted to either regulatory element in U2OS cells. A. The targeting and RNA assay strategy for dCas9-based transcriptional activators after localization to the HS2 enhancer is schematically depicted. **B.** Relative RNA expression from the HS2 enhancer and downstream *HBG1* promoter 72 hours post-transfection with 4 HS2-targeting gRNAs and the indicated dCas9-based transcriptional activators in U2OS cells (n=3 independent replicates, error bars indicate +/- SEM, * indicates adjusted *P*-value < 0.05 compared to dCas9 control-treated cells using two-tailed student t-test and correcting for multiple comparisons and false discovery rate (FDR) using two-stage linear step-up procedure of Benjamini, Krieger, and Yekutieli). **C.** The data points from Panel B are presented as scatter plots with Pearson correlation coefficients (r) and *P*-values (calculated using two-tailed student t-test) indicated. **D.** The targeting and RNA assay strategy for dCas9-based transcriptional activators after localization to the *HBG1* promoter is schematically depicted. **E.** Relative RNA expression from the HS2 enhancer and downstream *HBG1* promoter 72 hours post-transfection with 4 *HBG1*-targeting gRNAs and the indicated dCas9-based transcriptional activators in U2OS cells (n=3 independent replicates, error bars indicate +/- SEM, * indicates adjusted *P*-value < 0.05 compared to dCas9 control-treated cells using two-tailed student t-test and correcting for multiple comparisons and FDR using two-stage linear step-up procedure of Benjamini, Krieger, and Yekutieli). **F.** The data points from Panel E are presented as scatter plots with Pearson correlation coefficients (r) and *P*-values (calculated using two-tailed student t-test) indicated.

Supplemental Figure 13:

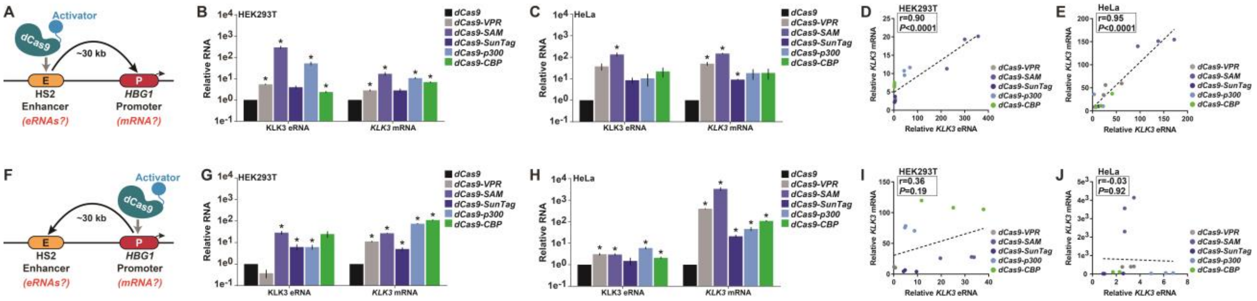

Supplemental Figure 13. dCas9-based transcriptional activators can induce RNA synthesis from both the *KLK3* promoter and the KLK3 enhancer when targeted to either regulatory element in HEK293T and HeLa cells. A. The targeting and RNA assay strategy for dCas9-based transcriptional activators after localization to the KLK3 enhancer is schematically depicted. **B and C.** Relative RNA expression from the KLK3 enhancer and downstream *KLK3* promoter 72 hours post-transfection with 4 KLK3 enhancer-targeting gRNAs and the indicated dCas9-based transcriptional activators in HEK293T and HeLa cells, respectively (n=3 independent replicates, error bars indicate +/- SEM, * indicates adjusted *P*-value < 0.05 compared to dCas9 control-treated cells using two-tailed student t-test and correcting for multiple comparisons and false discovery rate (FDR) using two-stage linear step-up procedure of Benjamini, Krieger, and Yekutieli). **D and E.** The data points from Panels B and C are presented as scatter plots with Pearson correlation coefficients (r) and *P*-values (calculated using two-tailed student t-test) indicated. **F**. The targeting and RNA assay strategy for dCas9-based transcriptional activators after localization to the *KLK3* promoter is schematically depicted. **G and H.** Relative RNA expression from the KLK3 enhancer and downstream *KLK3* promoter 72 hours post-transfection with 3 *KLK3* promoter-targeting gRNAs and the indicated dCas9-based transcriptional activators in HEK293T and HeLa cells, respectively (n=3 independent replicates, error bars indicate +/- SEM, * indicates adjusted *P*-value < 0.05 compared to dCas9 control-treated cells using two-tailed student t-test and correcting for multiple comparisons and FDR using two-stage linear step-up procedure of Benjamini, Krieger, and Yekutieli). **I and J.** The data points from Panels G and H are presented as scatter plots with Pearson correlation coefficients (r) and *P*-values (calculated using two-tailed student t-test) indicated.

Supplemental Figure 14:

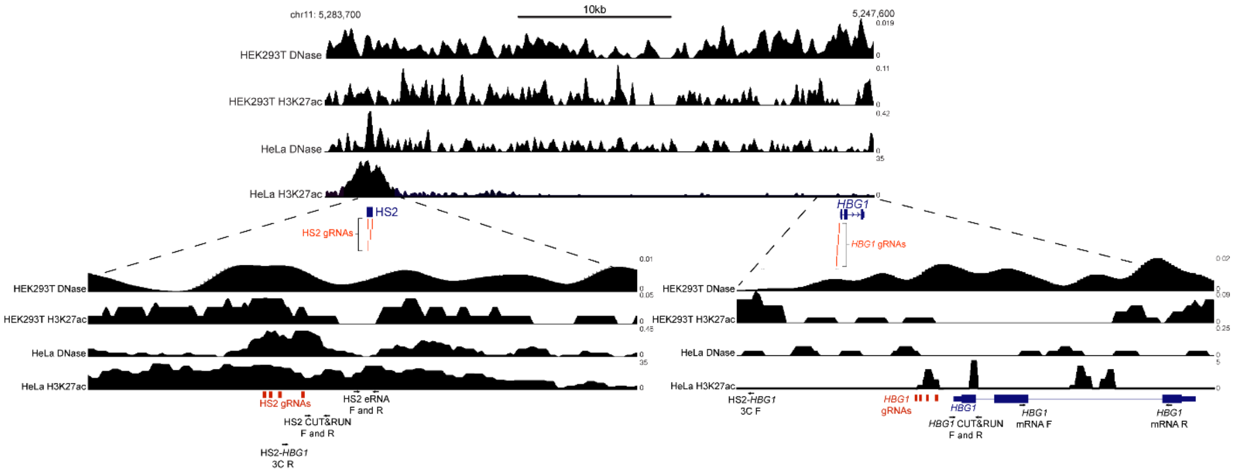

Supplemental Figure 14. Epigenomic context of the human β-globin locus and relevant assay landmarks in HEK293T and HeLa cells. The human chromosomal coordinates (using the GRCh38/hg38 reference genome) from chr11: 5,247,600 to chr11: 5,283,700 are shown along with DNase hypersensitivity (GEO accession GSM1635901, GSM1598310) and H3K27ac ChIP-seq data (GEO accession GSM4876229, GSM4542948) for HEK293T and HeLa cells, respectively. Relative positions of gRNAs and primer sets used for analysis in this study are also shown.

Supplemental Figure 15:

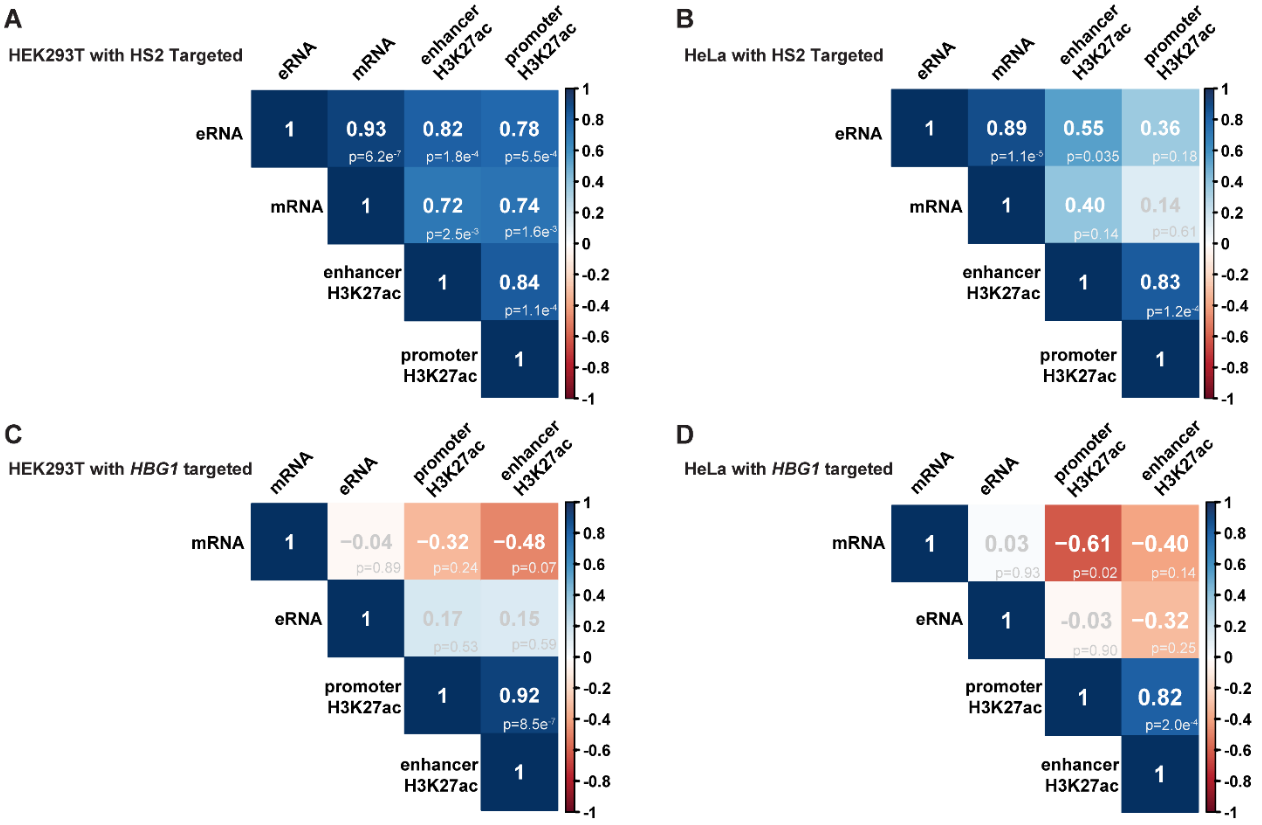

Supplemental Figure 15. Correlations between HS2 eRNA/*HBG1* mRNA levels and HS2/*HBG1* promoter H3K27ac. Correlation matrices showing the Pearson correlation coefficients (r) between data points from main text Figures 2B and 2G (Panel **A**), main text Figures 2C and 2H (Panel **B**), main text Figures 3B and 3G (Panel **C**), and main text Figures 3C and 3H (Panel **D**) respectively, along with corresponding *P*-values (calculated using two-tailed student t-test).

Supplemental Figure 16:

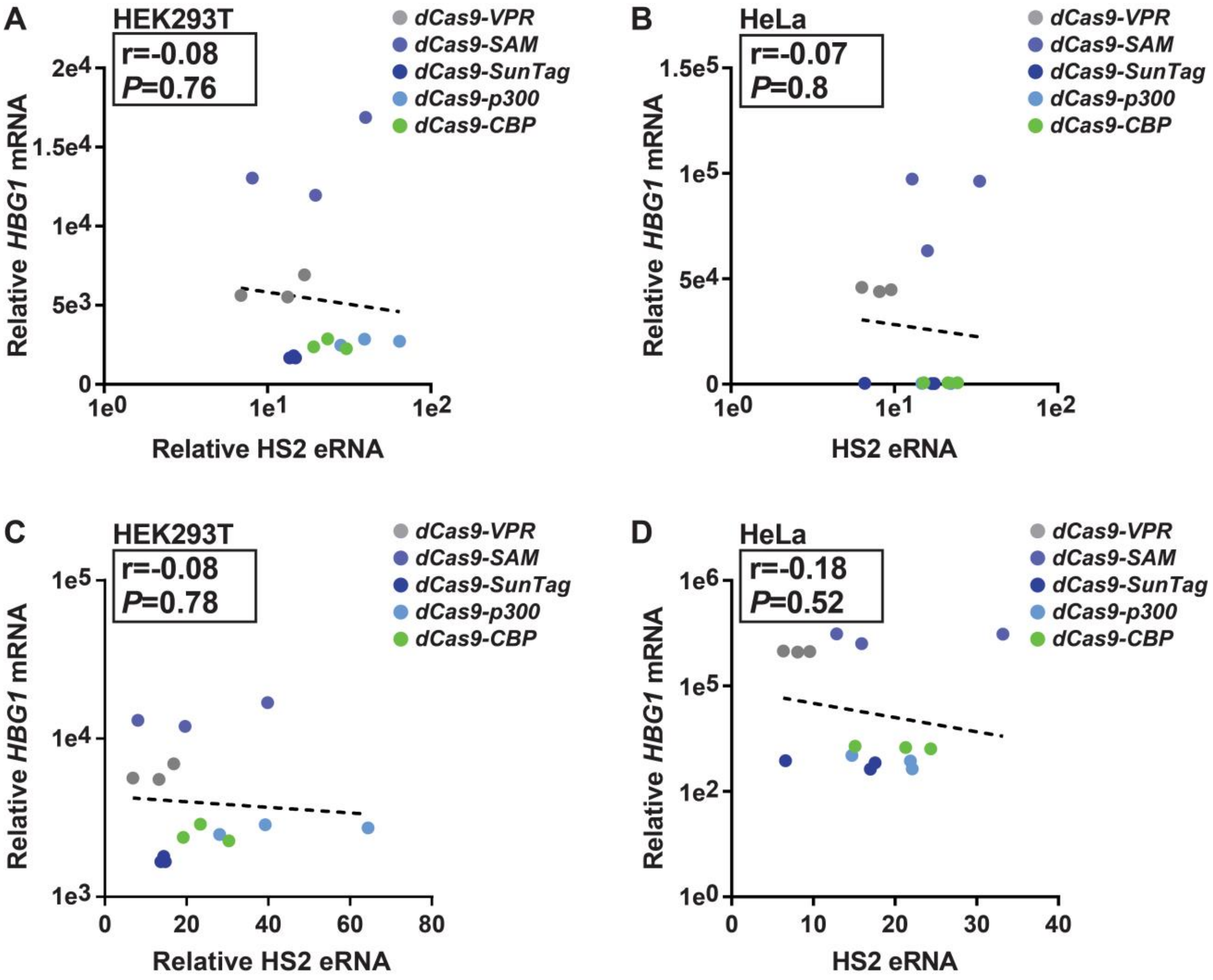

Supplemental Figure 16. *HBG1* mRNA and HS2 eRNA are not significantly positively correlated when the *HBG1* promoter is targeted by dCas9-based transcriptional activators. The data from main text Figures 3D (HEK293T cells) and 3E (HeLa cells), respectively, is shown after being plotted and regressed in linear-log space (Panels **A** and **B**) and after being plotted and regressed in log-linear space (Panels **C** and **D**) to enhance clarity and evaluate variance.

Supplemental Figure 17:

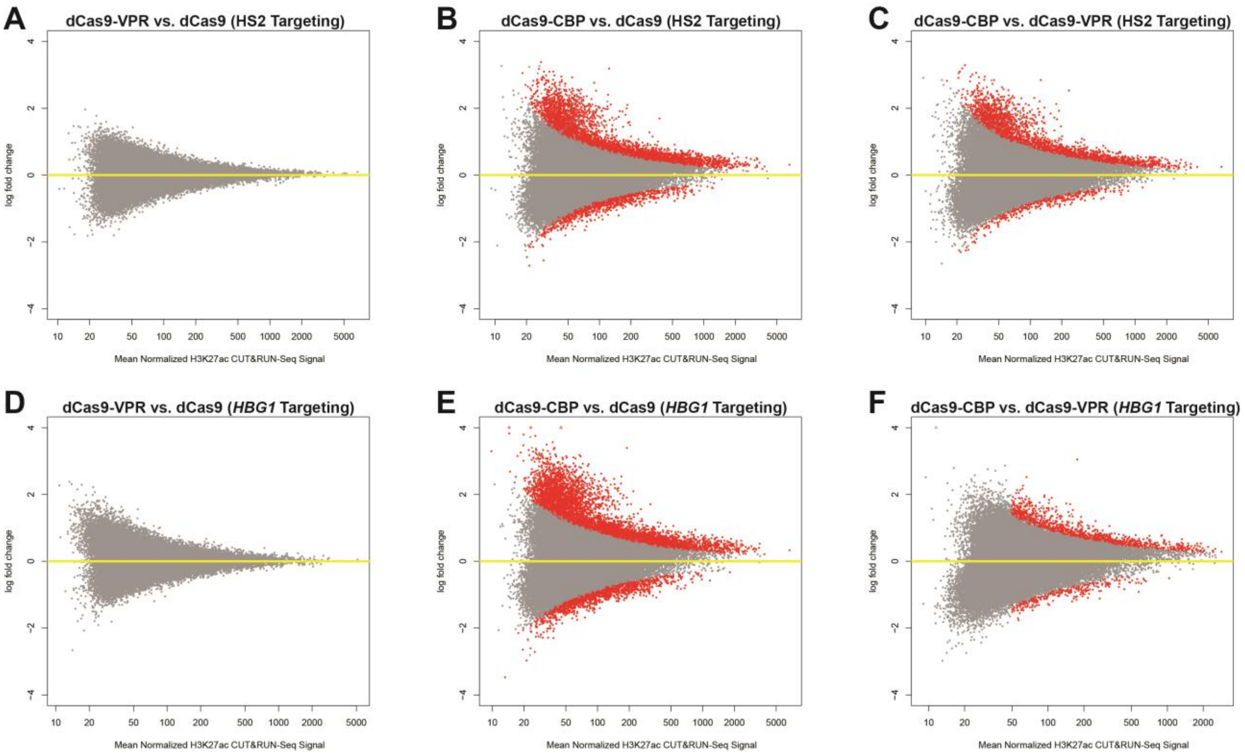

Supplemental Figure 17. Genome-wide analysis of differential H3K27ac enrichment after targeting the HS2 enhancer or *HBG1* promoter with dCas9-VPR or dCas9-CPB. A and B. Differential H3K27ac peaks relative to dCas9 when dCas9-VPR or dCas9-CBP, respectively, were targeted to the HS2 enhancer using 4 gRNAs. **C.** Differential H3K27ac peaks between dCas9-VPR and dCas9-CBP when each was targeted to the HS2 enhancer using 4 gRNAs. **D and E.** Differential H3K27ac peaks relative to dCas9 when dCas9-VPR or dCas9-CBP, respectively were targeted to the *HBG1* promoter using 4 gRNAs. **F.** Differential H3K27ac peaks between dCas9-VPR and dCas9-CBP when each was targeted to the *HBG1* promoter using 4 gRNAs. In all panels differences in H3K27ac enrichment were assessed and plotted using R (v4.1.3) and DESeq2 (v1.34.0) with an FDR cutoff of ≤ 0.05. Red dots indicate peaks with significant differences in H3K27ac enrichment in respective comparisons.

Supplemental Figure 18:

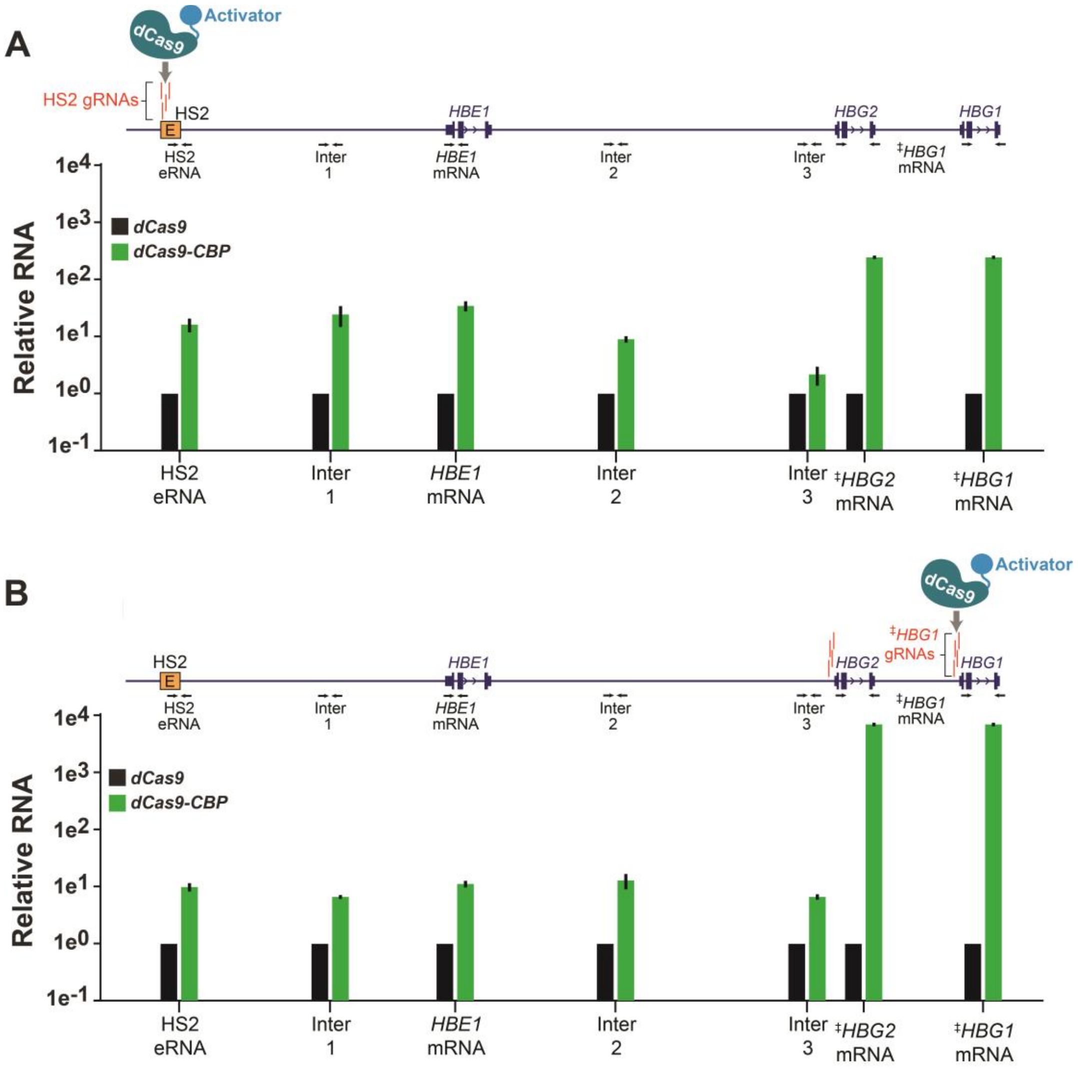

Supplemental Figure 18. Increases in transcription can spread between the HS2 enhancer and *HBG1* promoter when either regulatory element is targeted by dCas9-CBP in HeLa cells. A and B. Genomic coordinates on human chromosome 11 spanning ~5,282,828bp to ~5,247,660bp (GRCh38/hg38) are shown along with the relative expression of enhancer RNAs, intergenic ncRNAs, and mRNAs between the HS2 enhancer and *HBG1* promoter 72 hours post-transfection with the indicated dCas9-based transcriptional activators and 4 HS2-targeting gRNAs (panel **A**) or 4 *HBG1*-targeting gRNAs (panel **A**) in HEK293T cells. Target locations of RT-qPCR primers are indicated in corresponding locations on chromosome 11. n=2 independent replicates. ‡ serves to note that given the highly homologous genomic regions shared by *HBG1* and *HBG2*, gRNAs and RT-qPCR primers targeting *HBG1* also target *HBG2*.

Supplemental Figure 19:

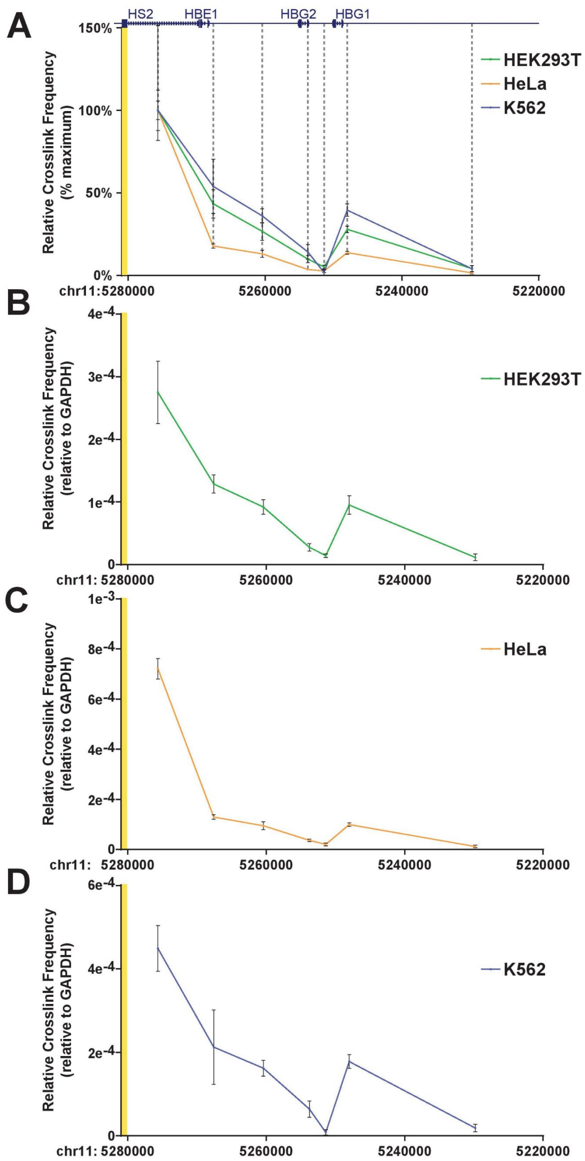

Supplemental Figure 19. Different cell types display different levels of basal contact between the HS2 enhancer and the *HBG1* region. 3C was carried out in untreated HEK293T, HeLa, and K562 cells. The crosslinking frequency between each HindIII fragment and the HS2 anchor fragment (yellow bar) is plotted at the fragment locations (dotted vertical lines). 3C values were normalized to the maximum crosslinking frequency for cell type in panel **A** or relative to *GAPDH* in panels **B-D**. RT-qPCR primer sequences and conditions are listed in **Supplemental Table 3**.

Supplemental Figure 20:

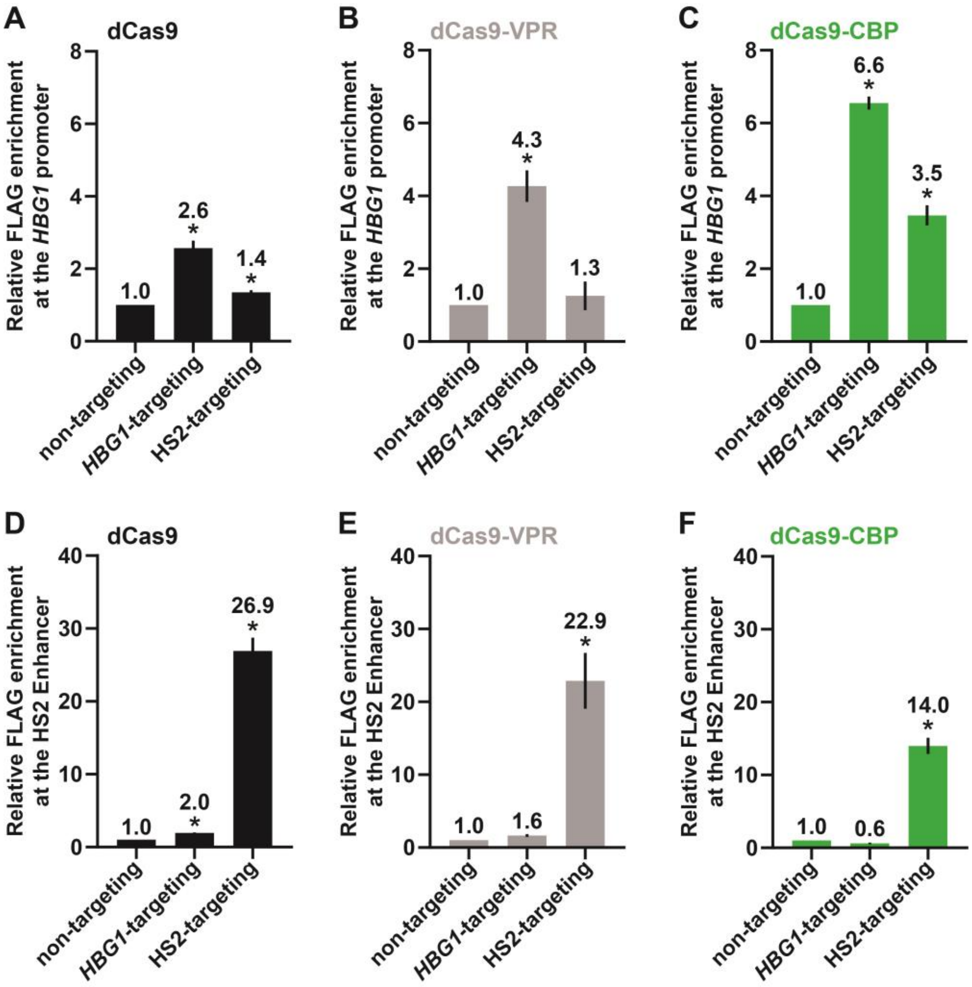

Supplemental Figure 20. Targeting dCas9-CBP to the HS2 enhancer results in the enrichment of dCas9-CBP at the *HBG1* promoter. A-C. Relative FLAG enrichment (all dCas9-based transcriptional activators are FLAG-tagged) at the *HBG1* promoter 72 hours post-transfection with the indicated dCas9-based transcriptional activators and either a non-targeting gRNA control, 4 *HBG1* promoter-targeting gRNAs, or 4 HS2-targeting gRNAs and in HEK293T cells (n=3 independent replicates, error bars indicate +/- SEM, * indicates adjusted *P*-value < 0.05 compared to dCas9 control-treated cells using two-tailed student t-test and correcting for multiple comparisons and false discovery rate (FDR) using two-stage linear step-up procedure of Benjamini, Krieger, and Yekutieli). **D-F.** Relative FLAG enrichment at the HS2 enhancer 72 hours post-transfection with the indicated dCas9-based transcriptional activators and either a non-targeting gRNA control, 4 *HBG1* promoter-targeting gRNAs, or 4 HS2-targeting gRNAs and in HEK293T cells (n=3 independent replicates, error bars indicate +/- SEM, * indicates adjusted *P*-value < 0.05 compared to dCas9 control-treated cells using two-tailed student t-test and correcting for multiple comparisons and FDR using two-stage linear step-up procedure of Benjamini, Krieger, and Yekutieli).

Supplemental Figure 21:

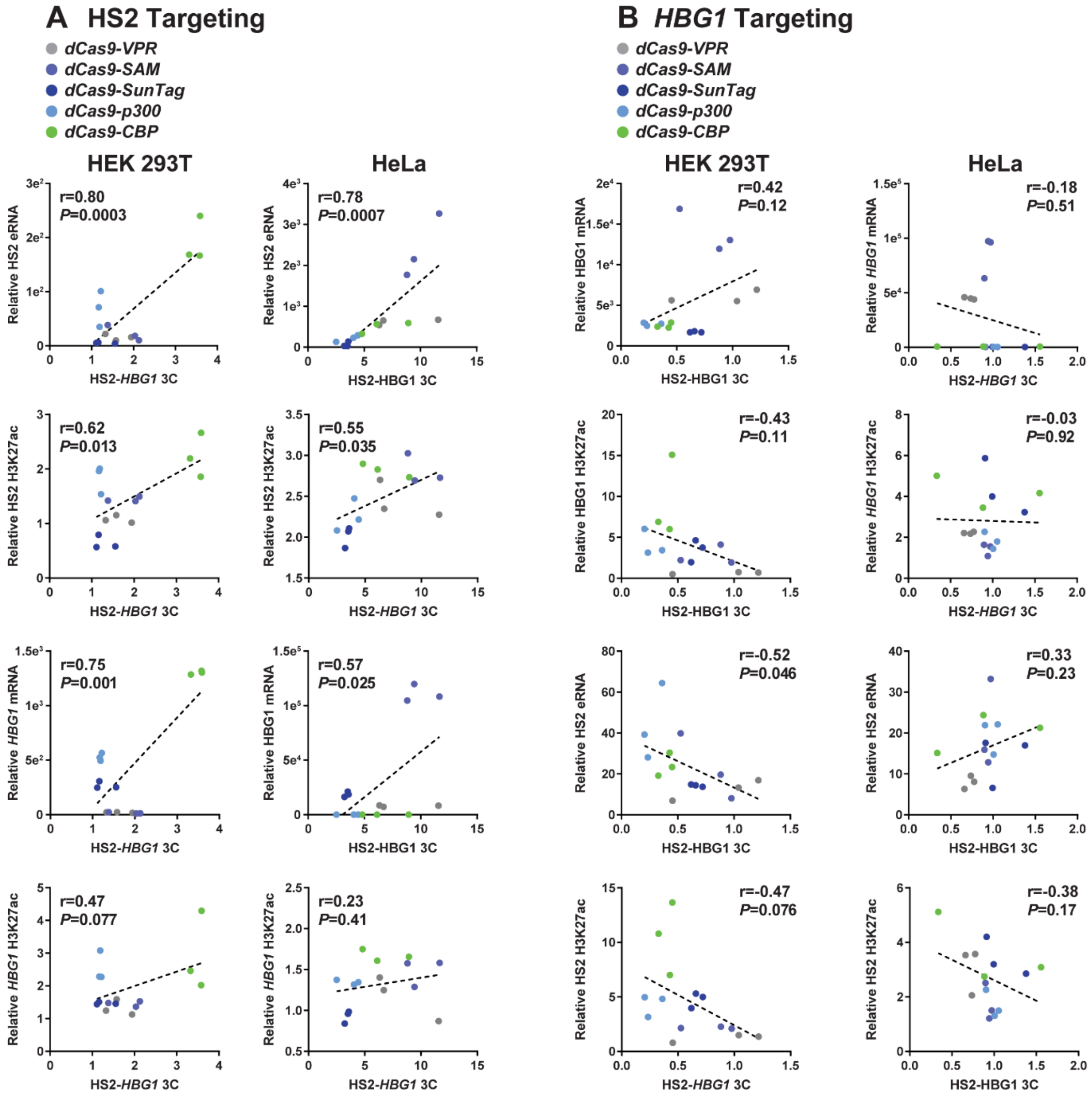

Supplemental Figure 21. Correlations between HS2/*HBG1* relative contact, RNA outputs, and H3K27ac enrichment. A. Pearson correlation coefficients (r) between 3C data from main text Figures 5B/5C and corresponding RNA levels and H3K27ac enrichment data from main text Figure 2, respectively, are shown along with associated *P*-values (calculated using two-tailed student t-test). **B.** The Pearson correlation coefficients (r) between 3C data from main text Figures 5F/5G and corresponding RNA levels and H3K27ac enrichment from main text Figure 3, respectively; are shown along with associated *P*-values (calculated using two-tailed student t-test).

Supplementary Note 1. Amino acid sequences of dCas9 constructs.

dCas9: amino acid sequence; Streptococcus pyogenes Cas9 (D10A, H840A), Nuclear Localization Sequence, 1 X “Flag” Epitope

MDKKYSIGLAIGTNSVGWAVITDEYKVPSKKFKVLGNTDRHSIKKNLIGALLFDSGETAEATRLKRTARRR
YTRRKNRICYLQEIFSNEMAKVDDSFFHRLEESFLVEEDKKHERHPIFGNIVDEVAYHEKYPTIYHLRKKLV
DSTDKADLRLIYLALAHMIKFRGHFLIEGDLNPDNSDVDKLFIQLVQTYNQLFEENPINASGVDAKAILSARL
SKSRRLENLIAQLPGEKKNGLFGNLIALSLGLTPNFKSNFDLAEDAKLQLSKDTYDDDLDNLLAQIGDQYA
DLFLAAKNLSDAILLSDILRVNTEITKAPLSASMIKRYDEHHQDLTLLKALVRQQLPEKYKEIFFDQSKNGYA
GYIDGGASQEEFYKFIKPILEKMDGTEELLVKLNREDLLRKQRTFDNGSIPHQIHLGELHAILRRQEDFYPF
LKDNREKIEKILTFRIPYYVGPLARGNSRFAWMTRKSEETITPWNFEEVVDKGASAQSFIERMTNFDKNLP
NEKVLPKHSLLYEYFTVYNELTKVKYVTEGMRKPAFLSGEQKKAIVDLLFKTNRKVTVKQLKEDYFKKIEC
FDSVEISGVEDRFNASLGTYHDLLKIIKDKDFLDNEENEDILEDIVLTLTLFEDREMIEERLKTYAHLFDDKV
MKQLKRRRYTGWGRLSRKLINGIRDKQSGKTILDFLKSDGFANRNFMQLIHDDSLTFKEDIQKAQVSGQG
DSLHEHIANLAGSPAIKKGILQTVKVVDELVKVMGRHKPENIVIEMARENQTTQKGQKNSRERMKRIEEGI
KELGSQILKEHPVENTQLQNEKLYLYYLQNGRDMYVDQELDINRLSDYDVDAIVPQSFLKDDSIDNKVLTR
SDKNRGKSDNVPSEEVVKKMKNYWRQLLNAKLITQRKFDNLTKAERGGLSELDKAGFIKRQLVETRQITK
HVAQILDSRMNTKYDENDKLIREVKVITLKSKLVSDFRKDFQFYKVREINNYHHAHDAYLNAVVGTALIKKY
PKLESEFVYGDYKVYDVRKMIAKSEQEIGKATAKYFFYSNIMNFFKTEITLANGEIRKRPLIETNGETGEIV
WDKGRDFATVRKVLSMPQVNIVKKTEVQTGGFSKESILPKRNSDKLIARKKDWDPKKYGGFDSPTVAYS
VLVVAKVEKGKSKKLKSVKELLGITIMERSSFEKNPIDFLEAKGYKEVKKDLIIKLPKYSLFELENGRKRMLA
SAGELQKGNELALPSKYVNFLYLASHYEKLKGSPEDNEQKQLFVEQHKHYLDEIIEQISEFSKRVILADANL
DKVLSAYNKHRDKPIREQAENIIHLFTLTNLGAPAAFKYFDTTIDRKRYTSTKEVLDATLIHQSITGLYETRID
LSQLGGDKRPAATKKAGQAKKKKDYKDDDDK

dCas9-VPR: amino acid sequence; Streptococcus pyogenes Cas9 (D10A, H840A), Nuclear Localization Sequence, VPR activation domain, 1 X “Flag” Epitope

MDKKYSIGLAIGTNSVGWAVITDEYKVPSKKFKVLGNTDRHSIKKNLIGALLFDSGETAEATRLKRTARRR
YTRRKNRICYLQEIFSNEMAKVDDSFFHRLEESFLVEEDKKHERHPIFGNIVDEVAYHEKYPTIYHLRKKLV
DSTDKADLRLIYLALAHMIKFRGHFLIEGDLNPDNSDVDKLFIQLVQTYNQLFEENPINASGVDAKAILSARL
SKSRRLENLIAQLPGEKKNGLFGNLIALSLGLTPNFKSNFDLAEDAKLQLSKDTYDDDLDNLLAQIGDQYA
DLFLAAKNLSDAILLSDILRVNTEITKAPLSASMIKRYDEHHQDLTLLKALVRQQLPEKYKEIFFDQSKNGYA
GYIDGGASQEEFYKFIKPILEKMDGTEELLVKLNREDLLRKQRTFDNGSIPHQIHLGELHAILRRQEDFYPF
LKDNREKIEKILTFRIPYYVGPLARGNSRFAWMTRKSEETITPWNFEEVVDKGASAQSFIERMTNFDKNLP
NEKVLPKHSLLYEYFTVYNELTKVKYVTEGMRKPAFLSGEQKKAIVDLLFKTNRKVTVKQLKEDYFKKIEC
FDSVEISGVEDRFNASLGTYHDLLKIIKDKDFLDNEENEDILEDIVLTLTLFEDREMIEERLKTYAHLFDDKV

MKQLKRRRYTGWGRLSRKLINGIRDKQSGKTILDFLKSDGFANRNFMQLIHDDSLTFKEDIQKAQVSGQG
DSLHEHIANLAGSPAIKKGILQTVKVVDELVKVMGRHKPENIVIEMARENQTTQKGQKNSRERMKRIEEGI
KELGSQILKEHPVENTQLQNEKLYLYYLQNGRDMYVDQELDINRLSDYDVDAIVPQSFLKDDSIDNKVLTR
SDKNRGKSDNVPSEEVVKKMKNYWRQLLNAKLITQRKFDNLTKAERGGLSELDKAGFIKRQLVETRQITK
HVAQILDSRMNTKYDENDKLIREVKVITLKSKLVSDFRKDFQFYKVREINNYHHAHDAYLNAVVGTALIKKY
PKLESEFVYGDYKVYDVRKMIAKSEQEIGKATAKYFFYSNIMNFFKTEITLANGEIRKRPLIETNGETGEIV
WDKGRDFATVRKVLSMPQVNIVKKTEVQTGGFSKESILPKRNSDKLIARKKDWDPKKYGGFDSPTVAYS
VLVVAKVEKGKSKKLKSVKELLGITIMERSSFEKNPIDFLEAKGYKEVKKDLIIKLPKYSLFELENGRKRMLA
SAGELQKGNELALPSKYVNFLYLASHYEKLKGSPEDNEQKQLFVEQHKHYLDEIIEQISEFSKRVILADANL
DKVLSAYNKHRDKPIREQAENIIHLFTLTNLGAPAAFKYFDTTIDRKRYTSTKEVLDATLIHQSITGLYETRID
LSQLGGDKRPAATKKAGQAKKKKGSSPGIRRLDALISTSLYKKAGYKEASGSGRADALDDFDLDMLGSD
ALDDFDLDMLGSDALDDFDLDMLGSDALDDFDLDMLINSRSSGSPKKKRKVGSQYLPDTDDRHRIEEKR
KRTYETFKSIMKKSPFSGPTDPRPPPRRIAVPSRSSASVPKPAPQPYPFTSSLSTINYDEFPTMVFPSGQI
SQASALAPAPPQVLPQAPAPAPAPAMVSALAQAPAPVPVLAPGPPQAVAPPAPKPTQAGEGTLSEALLQ
LQFDDEDLGALLGNSTDPAVFTDLASVDNSEFQQLLNQGIPVAPHTTEPMLMEYPEAITRLVTGAQRPPD
PAPAPLGAPGLPNGLLSGDEDFSSIADMDFSALLGSGSGSRDSREGMFLPKPEAGSAISDVFEGREVCQ
PKRIRPFHPPGSPWANRPLPASLAPTPTGPVHEPVGSLTPAPVPQPLDPAPAVTPEASHLLEDPDEETSQ
AVKALREMADTVIPQKEEAAICGQMDLSHPPPRGHLDELTTTLESMTEDLNLDSPLTPELNEILDTFLNDE
CLLHAMHISTGLSIFDTSLFGSDYKDDDDK

dCas9-VP64: amino acid sequence; Nuclear Localization Sequence, Streptococcus pyogenes Cas9 (D10A, H840A)**, Nuclear Localization Sequence**, VP64 activation domain, 1 X “Flag” Epitope

KRPAATKKAGQAKKKKDKKYSIGLAIGTNSVGWAVITDEYKVPSKKFKVLGNTDRHSIKKNLIGALLFDSG
ETAEATRLKRTARRRYTRRKNRICYLQEIFSNEMAKVDDSFFHRLEESFLVEEDKKHERHPIFGNIVDEVA
YHEKYPTIYHLRKKLVDSTDKADLRLIYLALAHMIKFRGHFLIEGDLNPDNSDVDKLFIQLVQTYNQLFEEN
PINASGVDAKAILSARLSKSRRLENLIAQLPGEKKNGLFGNLIALSLGLTPNFKSNFDLAEDAKLQLSKDTY
DDDLDNLLAQIGDQYADLFLAAKNLSDAILLSDILRVNTEITKAPLSASMIKRYDEHHQDLTLLKALVRQQLP
EKYKEIFFDQSKNGYAGYIDGGASQEEFYKFIKPILEKMDGTEELLVKLNREDLLRKQRTFDNGSIPHQIHL
GELHAILRRQEDFYPFLKDNREKIEKILTFRIPYYVGPLARGNSRFAWMTRKSEETITPWNFEEVVDKGAS
AQSFIERMTNFDKNLPNEKVLPKHSLLYEYFTVYNELTKVKYVTEGMRKPAFLSGEQKKAIVDLLFKTNRK
VTVKQLKEDYFKKIECFDSVEISGVEDRFNASLGTYHDLLKIIKDKDFLDNEENEDILEDIVLTLTLFEDREMI
EERLKTYAHLFDDKVMKQLKRRRYTGWGRLSRKLINGIRDKQSGKTILDFLKSDGFANRNFMQLIHDDSL
TFKEDIQKAQVSGQGDSLHEHIANLAGSPAIKKGILQTVKVVDELVKVMGRHKPENIVIEMARENQTTQKG
QKNSRERMKRIEEGIKELGSQILKEHPVENTQLQNEKLYLYYLQNGRDMYVDQELDINRLSDYDVDHIVP
QSFLKDDSIDNKVLTRSDKARGKSDNVPSEEVVKKMKNYWRQLLNAKLITQRKFDNLTKAERGGLSELDK
AGFIKRQLVETRQITKHVAQILDSRMNTKYDENDKLIREVKVITLKSKLVSDFRKDFQFYKVREINNYHHAH

DAYLNAVVGTALIKKYPKLESEFVYGDYKVYDVRKMIAKSEQEIGKATAKYFFYSNIMNFFKTEITLANGEIR
KRPLIETNGETGEIVWDKGRDFATVRKVLSMPQVNIVKKTEVQTGGFSKESILPKRNSDKLIARKKDWDPK
KYGGFDSPTVAYSVLVVAKVEKGKSKKLKSVKELLGITIMERSSFEKNPIDFLEAKGYKEVKKDLIIKLPKYS
LFELENGRKRMLASAGELQKGNELALPSKYVNFLYLASHYEKLKGSPEDNEQKQLFVEQHKHYLDEIIEQI
SEFSKRVILADANLDKVLSAYNKHRDKPIREQAENIIHLFTLTNLGAPAAFKYFDTTIDRKRYTSTKEVLDAT
LIHQSITGLYETRIDLSQLGGDSAGGGGSGGGGSGGGGSGPKKKRKVAAAGSGRADALDDFDLDMLGS
DALDDFDLDMLGSDALDDFDLDMLGSDALDDFDLDMLINCTDYKDDDDK

MS2-P65-HSF1: amino acid sequence; **MS2-N55K**, Nuclear Localization Sequence, P65, HSF1, 1 X "Flag" Epitope

**ASNFTQFVLVDNGGTGDVTVAPSNFANGVAEWISSNSRSQAYKVTCSVRQSSAQKRKYTIKVEVPKVA
TQTVGGVELPVAAWRSYLNMELTIPIFATNSDCELIVKAMQGLLKDGNPIPSAIAANSGIY**SAGGGGSGG
GGSGGGGSGPKKKRKVAAAGSPSGQISNQALALAPSSAPVLAQTMVPSSAMVPLAQPPAPAPVLTPGP
PQSLSAPVPKSTQAGEGTLSEALLHLQFDADEDLGALLGNSTDPGVFTDLASVDNSEFQQLLNQGVSMS
HSTAEPMLMEYPEAITRLVTGSQRPPDPAPTPLGTSGLPNGLSGDEDFSSIADMDFSALLSQISSSGQGG
GGSGFSVDTSALLDLFSPSVTVPDMSLPDLDSSLASIQELLSPQEPPRPPEAENSSPDSGKQLVHYTAQP
LFLLDPGSVDTGSNDLPVLFELGEGSYFSEGDGFAEDPTISLLTGSEPPKAKDPTVSASDYKDDDDK

dCas9-10xGCN4; amino acid sequence; Nuclear Localization Sequence, Streptococcus pyogenes Cas9 (D10A, H840A), "HA" Epitope, 2x Nuclear Localization Sequence, 10xGCN4 peptide, 1 X "Flag" Epitope, Nuclear Localization Sequence

PKKKRKVGRVCRISSLRYRGPGIATMDKKYSIGLAIGTNSVGWAVITDEYKVPSKKFKVLGNTDRHSIKKN
LIGALLFDSGETAEATRLKRTARRRYTRRKNRICYLQEIFSNEMAKVDDSFFHRLEESFLVEEDKKHERHPI
FGNIVDEVAYHEKYPTIYHLRKKLVDSTDKADLRLIYLALAHMIKFRGHFLIEGDLNPDNSDVDKLFIQLVQT
YNQLFEENPINASGVDAKAILSARLSKSRRLENLIAQLPGEKKNGLFGNLIALSLGLTPNFKSNFDLAEDAK
LQLSKDTYDDDLDNLLAQIGDQYADLFLAAKNLSDAILLSDILRVNTEITKAPLSASMIKRYDEHHQDLTLLK
ALVRQQLPEKYKEIFFDQSKNGYAGYIDGGASQEEFYKFIKPILEKMDGTEELLVKLNREDLLRKQRTFDN
GSIPHQIHLGELHAILRRQEDFYPFLKDNREKIEKILTFRIPYYVGPLARGNSRFAWMTRKSEETITPWNFE
EVVDKGASAQSFIERMTNFDKNLPNEKVLPKHSLLYEYFTVYNELTKVKYVTEGMRKPAFLSGEQKKAIV
DLLFKTNRKVTVKQLKEDYFKKIECFDSVEISGVEDRFNASLGTYHDLLKIIKDKDFLDNEENEDILEDIVLTL
TLFEDREMIEERLKTYAHLFDDKVMKQLKRRRYTGWGRLSRKLINGIRDKQSGKTILDFLKSDGFANRNF
MQLIHDDSLTFKEDIQKAQVSGQGDSLHEHIANLAGSPAIKKGILQTVKVVDELVKVMGRHKPENIVIEMAR
ENQTTQKGQKNSRERMKRIEEGIKELGSQILKEHPVENTQLQNEKLYLYYLQNGRDMYVDQELDINRLSD
YDVDAIVPQSFLKDDSIDNKVLTRSDKNRGKSDNVPSEEVVKKMKNYWRQLLNAKLITQRKFDNLTKAER
GGLSELDKAGFIKRQLVETRQITKHVAQILDSRMNTKYDENDKLIREVKVITLKSKLVSDFRKDFQFYKVRE

INNYHHAHDAYLNAVVGTALIKKYPKLESEFVYGDYKVYDVRKMIAKSEQEIGKATAKYFFYSNIMNFFKTE
ITLANGEIRKRPLIETNGETGEIVWDKGRDFATVRKVLSMPQVNIVKKTEVQTGGFSKESILPKRNSDKLIA
RKKDWDPKKYGGFDSPTVAYSVLVVAKVEKGKSKKLKSVKELLGITIMERSSFEKNPIDFLEAKGYKEVKK
DLIIKLPKYSLFELENGRKRMLASAGELQKGNELALPSKYVNFLYLASHYEKLKGSPEDNEQKQLFVEQHK
HYLDEIIEQISEFSKRVILADANLDKVLSAYNKHRDKPIREQAENIIHLFTLTNLGAPAAFKYFDTTIDRKRYT
STKEVLDATLIHQSITGLYETRIDLSQLGGDAYPYDVPDYASLGSGSPKKKRKVEDPKKKRKVDGIGSGSN
GSSGSNGPTDAAEEELLSKNYHLENEVARLKKGSGSGEELLSKNYHLENEVARLKKGSGSGEELLSKNY
HLENEVARLKKGSGSGEELLSKNYHLENEVARLKKGSGSGEELLSKNYHLENEVARLKKGSGSGEELLS
KNYHLENEVARLKKGSGSGEELLSKNYHLENEVARLKKGSGSGEELLSKNYHLENEVARLKKGSGSGEE
LLSKNYHLENEVARLKKGSGSGEELLSKNYHLENEVARLKKGSGSGTAVNIGGGTGPMDLQRPDYKDDD
DKRPQGGGGPKKKRKV

scFv-GCN4-sfGFP-VP64-GB1: amino acid sequence; scFv-GCN4, “HA” Epitope, superfolded GFP, VP64 activation domain, B1 domain of Streptococcal protein G, 1 X “Flag” Epitope, Nuclear Localization Sequence

MGPDIVMTQSPSSLSASVGDRVTITCRSSTGAVTTSNYASWVQEKPGKLFKGLIGGTNNRAPGVPSRFS
GSLIGDKATLTISSLQPEDFATYFCALWYSNHWVFGQGTKVELKRGGGGSGGGGSGGGGSSGGGSEV
KLLESGGGLVQPGGSLKLSCAVSGFSLTDYGVNWVRQAPGRGLEWIGVIWGDGITDYNSALKDRFIISKD
NGKNTVYLQMSKVRSDDTALYYCVTGLFDYWGQGTLVTVSSYPYDVPDYAGGGGGSGGGGSGGGGS
GGGGSLDPGGGGSGSKGEELFTGVVPILVELDGDVNGHKFSVRGEGEGDATNGKLTLKFICTTGKLPVP
WPTLVTTLTYGVQCFSRYPDHMKRHDFFKSAMPEGYVQERTISFKDDGTYKTRAEVKFEGDTLVNRIELK
GIDFKEDGNILGHKLEYNFNSHNVYITADKQKNGIKANFKIRHNVEDGSVQLADHYQQNTPIGDGPVLLPD
NHYLSTQSVLSKDPNEKRDHMVLLEFVTAAGITHGMDELYKGGGRTGGGGGDALDDFDLDMLGSDAL
DDFDLDMLGSDALDDFDLDMLGSDALDDFDLDMLGSGGGSRTEEYKLILNGKTLKGETTTEAVDAATAE
KVFKQYANDNGVDGEWTYDDATKTFTVTEGGGSGGGTSDYKDDDDKTSPKTRRRPRRSQRKRPPT

dCas9-p300: amino acid sequence; Streptococcus pyogenes Cas9 (D10A, H840A), Nuclear Localization Sequence, Human p300 aa 1048-1664, 1 X “Flag” Epitope

MDKKYSIGLAIGTNSVGWAVITDEYKVPSKKFKVLGNTDRHSIKKNLIGALLFDSGETAEATRLKRTARRR
YTRRKNRICYLQEIFSNEMAKVDDSFFHRLEESFLVEEDKKHERHPIFGNIVDEVAYHEKYPTIYHLRKKLV
DSTDKADLRLIYLALAHMIKFRGHFLIEGDLNPDNSDVDKLFIQLVQTYNQLFEENPINASGVDAKAILSARL
SKSRRLENLIAQLPGEKKNGLFGNLIALSLGLTPNFKSNFDLAEDAKLQLSKDTYDDDLDNLLAQIGDQYA
DLFLAAKNLSDAILLSDILRVNTEITKAPLSASMIKRYDEHHQDLTLLKALVRQQLPEKYKEIFFDQSKNGYA
GYIDGGASQEEFYKFIKPILEKMDGTEELLVKLNREDLLRKQRTFDNGSIPHQIHLGELHAILRRQEDFYPF
LKDNREKIEKILTFRIPYYVGPLARGNSRFAWMTRKSEETITPWNFEEVVDKGASAQSFIERMTNFDKNLP
NEKVLPKHSLLYEYFTVYNELTKVKYVTEGMRKPAFLSGEQKKAIVDLLFKTNRKVTVKQLKEDYFKKIEC

FDSVEISGVEDRFNASLGTYHDLLKIIKDKDFLDNEENEDILEDIVLTLTLFEDREMIEERLKTYAHLFDDKV
MKQLKRRRYTGWGRLSRKLINGIRDKQSGKTILDFLKSDGFANRNFMQLIHDDSLTFKEDIQKAQVSGQG
DSLHEHIANLAGSPAIKKGILQTVKVVDELVKVMGRHKPENIVIEMARENQTTQKGQKNSRERMKRIEEGI
KELGSQILKEHPVENTQLQNEKLYLYYLQNGRDMYVDQELDINRLSDYDVDAIVPQSFLKDDSIDNKVLTR
SDKNRGKSDNVPSEEVVKKMKNYWRQLLNAKLITQRKFDNLTKAERGGLSELDKAGFIKRQLVETRQITK
HVAQILDSRMNTKYDENDKLIREVKVITLKSKLVSDFRKDFQFYKVREINNYHHAHDAYLNAVVGTALIKKY
PKLESEFVYGDYKVYDVRKMIAKSEQEIGKATAKYFFYSNIMNFFKTEITLANGEIRKRPLIETNGETGEIV
WDKGRDFATVRKVLSMPQVNIVKKTEVQTGGFSKESILPKRNSDKLIARKKDWDPKKYGGFDSPTVAYS
VLVVAKVEKGKSKKLKSVKELLGITIMERSSFEKNPIDFLEAKGYKEVKKDLIIKLPKYSLFELENGRKRMLA
SAGELQKGNELALPSKYVNFLYLASHYEKLKGSPEDNEQKQLFVEQHKHYLDEIIEQISEFSKRVILADANL
DKVLSAYNKHRDKPIREQAENIIHLFTLTNLGAPAAFKYFDTTIDRKRYTSTKEVLDATLIHQSITGLYETRID
LSQLGGDKRPAATKKAGQAKKKKGSIFKPEELRQALMPTLEALYRQDPESLPFRQPVDPQLLGIPDYFDIV
KSPMDLSTIKRKLDTGQYQEPWQYVDDIWLMFNNAWLYNRKTSRVYKYCSKLSEVFEQEIDPVMQSLGY
CCGRKLEFSPQTLCCYGKQLCTIPRDATYYSYQNRYHFCEKCFNEIQGESVSLGDDPSQPQTTINKEQFS
KRKNDTLDPELFVECTECGRKMHQICVLHHEIIWPAGFVCDGCLKKSARTRKENKFSAKRLPSTRLGTFL
ENRVNDFLRRQNHPESGEVTVRVVHASDKTVEVKPGMKARFVDSGEMAESFPYRTKALFAFEEIDGVDL
CFFGMHVQEYGSDCPPPNQRRVYISYLDSVHFFRPKCLRTAVYHEILIGYLEYVKKLGYTTGHIWACPPS
EGDDYIFHCHPPDQKIPKPKRLQEWYKKMLDKAVSERIVHDYKDIFKQATEDRLTSAKELPYFEGDFWPN
VLEESIKELEQEEEERKREENTSNESTDVTKGDSKNAKKKNNKKTSKNKSSLSRGNKKKPGMPNVSNDL
SQKLYATMEKHKEVFFVIRLIAGPAANSLPPIVDPDPLIPCDLMDGRDAFLTLARDKHLEFSSLRRAQWST
MCMLVELHTQSQDGSDYKDDDDK

dCas9-CBP: amino acid sequence; Streptococcus pyogenes Cas9 (D10A, H840A), Nuclear Localization Sequence, Human CBP aa 1087-1701, 1 X “Flag” Epitope

MDKKYSIGLAIGTNSVGWAVITDEYKVPSKKFKVLGNTDRHSIKKNLIGALLFDSGETAEATRLKRTARRR
YTRRKNRICYLQEIFSNEMAKVDDSFFHRLEESFLVEEDKKHERHPIFGNIVDEVAYHEKYPTIYHLRKKLV
DSTDKADLRLIYLALAHMIKFRGHFLIEGDLNPDNSDVDKLFIQLVQTYNQLFEENPINASGVDAKAILSARL
SKSRRLENLIAQLPGEKKNGLFGNLIALSLGLTPNFKSNFDLAEDAKLQLSKDTYDDDLDNLLAQIGDQYA
DLFLAAKNLSDAILLSDILRVNTEITKAPLSASMIKRYDEHHQDLTLLKALVRQQLPEKYKEIFFDQSKNGYA
GYIDGGASQEEFYKFIKPILEKMDGTEELLVKLNREDLLRKQRTFDNGSIPHQIHLGELHAILRRQEDFYPF
LKDNREKIEKILTFRIPYYVGPLARGNSRFAWMTRKSEETITPWNFEEVVDKGASAQSFIERMTNFDKNLP
NEKVLPKHSLLYEYFTVYNELTKVKYVTEGMRKPAFLSGEQKKAIVDLLFKTNRKVTVKQLKEDYFKKIEC
FDSVEISGVEDRFNASLGTYHDLLKIIKDKDFLDNEENEDILEDIVLTLTLFEDREMIEERLKTYAHLFDDKV
MKQLKRRRYTGWGRLSRKLINGIRDKQSGKTILDFLKSDGFANRNFMQLIHDDSLTFKEDIQKAQVSGQG
DSLHEHIANLAGSPAIKKGILQTVKVVDELVKVMGRHKPENIVIEMARENQTTQKGQKNSRERMKRIEEGI
KELGSQILKEHPVENTQLQNEKLYLYYLQNGRDMYVDQELDINRLSDYDVDAIVPQSFLKDDSIDNKVLTR

SDKNRGKSDNVPSEEVVKKMKNYWRQLLNAKLITQRKFDNLTKAERGGLSELDKAGFIKRQLVETRQITK
HVAQILDSRMNTKYDENDKLIREVKVITLKSKLVSDFRKDFQFYKVREINNYHHAHDAYLNAVVGTALIKKY
PKLESEFVYGDYKVYDVRKMIAKSEQEIGKATAKYFFYSNIMNFFKTEITLANGEIRKRPLIETNGETGEIV
WDKGRDFATVRKVLSMPQVNIVKKTEVQTGGFSKESILPKRNSDKLIARKKDWDPKKYGGFDSPTVAYS
VLVVAKVEKGKSKKLKSVKELLGITIMERSSFEKNPIDFLEAKGYKEVKKDLIIKLPKYSLFELENGRKRMLA
SAGELQKGNELALPSKYVNFLYLASHYEKLKGSPEDNEQKQLFVEQHKHYLDEIIEQISEFSKRVILADANL
DKVLSAYNKHRDKPIREQAENIIHLFTLTNLGAPAAFKYFDTTIDRKRYTSTKEVLDATLIHQSITGLYETRID
LSQLGGDKRPAATKKAGQAKKKKGSIFKPEELRQALMPTLEALYRQDPESLPFRQPVDPQLLGIPDYFDIV
KNPMDLSTIKRKLDTGQYQEPWQYVDDVWLMFNNAWLYNRKTSRVYKFCSKLAEVFEQEIDPVMQSLG
YCCGRKYEFSPQTLCCYGKQLCTIPRDAAYYSYQNRYHFCEKCFTEIQGENVTLGDDPSQPQTTISKDQF
EKKKNDTLDPEPFVDCKECGRKMHQICVLHYDIIWPSGFVCDNCLKKTGRPRKENKFSAKRLQTTRLGN
HLEDRVNKFLRRQNHPEAGEVFVRVVASSDKTVEVKPGMKSRFVDSGEMSESFPYRTKALFAFEEIDGV
DVCFFGMHVQEYGSDCPPPNTRRVYISYLDSIHFFRPRCLRTAVYHEILIGYLEYVKKLGYVTGHIWACPP
SEGDDYIFHCHPPDQKIPKPKRLQEWYKKMLDKAFAERIIHDYKDIFKQATEDRLTSAKELPYFEGDFWPN
VLEESIKELEQEEEERKKEESTAASETTEGSQGDSKNAKKKNNKKTNKNKSSISRANKKKPSMPNVSNDL
SQKLYATMEKHKEVFFVIHLHAGPVINTLPPIVDPDPLLSCDLMDGRDAFLTLARDKHWEFSSLRRSKWS
TLCMLVELHTQGQDGSDYKDDDDK

dCas9-p300 HAT: amino acid sequence; Streptococcus pyogenes Cas9 (D10A, H840A), Nuclear Localization Sequence, Human p300 aa 1287-1664, 1 X “Flag” Epitope

MDKKYSIGLAIGTNSVGWAVITDEYKVPSKKFKVLGNTDRHSIKKNLIGALLFDSGETAEATRLKRTARRR
YTRRKNRICYLQEIFSNEMAKVDDSFFHRLEESFLVEEDKKHERHPIFGNIVDEVAYHEKYPTIYHLRKKLV
DSTDKADLRLIYLALAHMIKFRGHFLIEGDLNPDNSDVDKLFIQLVQTYNQLFEENPINASGVDAKAILSARL
SKSRRLENLIAQLPGEKKNGLFGNLIALSLGLTPNFKSNFDLAEDAKLQLSKDTYDDDLDNLLAQIGDQYA
DLFLAAKNLSDAILLSDILRVNTEITKAPLSASMIKRYDEHHQDLTLLKALVRQQLPEKYKEIFFDQSKNGYA
GYIDGGASQEEFYKFIKPILEKMDGTEELLVKLNREDLLRKQRTFDNGSIPHQIHLGELHAILRRQEDFYPF
LKDNREKIEKILTFRIPYYVGPLARGNSRFAWMTRKSEETITPWNFEEVVDKGASAQSFIERMTNFDKNLP
NEKVLPKHSLLYEYFTVYNELTKVKYVTEGMRKPAFLSGEQKKAIVDLLFKTNRKVTVKQLKEDYFKKIEC
FDSVEISGVEDRFNASLGTYHDLLKIIKDKDFLDNEENEDILEDIVLTLTLFEDREMIEERLKTYAHLFDDKV
MKQLKRRRYTGWGRLSRKLINGIRDKQSGKTILDFLKSDGFANRNFMQLIHDDSLTFKEDIQKAQVSGQG
DSLHEHIANLAGSPAIKKGILQTVKVVDELVKVMGRHKPENIVIEMARENQTTQKGQKNSRERMKRIEEGI
KELGSQILKEHPVENTQLQNEKLYLYYLQNGRDMYVDQELDINRLSDYDVDAIVPQSFLKDDSIDNKVLTR
SDKNRGKSDNVPSEEVVKKMKNYWRQLLNAKLITQRKFDNLTKAERGGLSELDKAGFIKRQLVETRQITK
HVAQILDSRMNTKYDENDKLIREVKVITLKSKLVSDFRKDFQFYKVREINNYHHAHDAYLNAVVGTALIKKY
PKLESEFVYGDYKVYDVRKMIAKSEQEIGKATAKYFFYSNIMNFFKTEITLANGEIRKRPLIETNGETGEIV
WDKGRDFATVRKVLSMPQVNIVKKTEVQTGGFSKESILPKRNSDKLIARKKDWDPKKYGGFDSPTVAYS

VLVVAKVEKGKSKKLKSVKELLGITIMERSSFEKNPIDFLEAKGYKEVKKDLIIKLPKYSLFELENGRKRMLA
SAGELQKGNELALPSKYVNFLYLASHYEKLKGSPEDNEQKQLFVEQHKHYLDEIIEQISEFSKRVILADANL
DKVLSAYNKHRDKPIREQAENIIHLFTLTNLGAPAAFKYFDTTIDRKRYTSTKEVLDATLIHQSITGLYETRID
LSQLGGDKRPAATKKAGQAKKKKGSKFSAKRLPSTRLGTFLENRVNDFLRRQNHPESGEVTVRVVHASD
KTVEVKPGMKARFVDSGEMAESFPYRTKALFAFEEIDGVDLCFFGMHVQEYGSDCPPPNQRRVYISYLD
SVHFFRPKCLRTAVYHEILIGYLEYVKKLGYTTGHIWACPPSEGDDYIFHCHPPDQKIPKPKRLQEWYKKM
LDKAVSERIVHDYKDIFKQATEDRLTSAKELPYFEGDFWPNVLEESIKELEQEEEERKREENTSNESTDVT
KGDSKNAKKKNNKKTSKNKSSLSRGNKKKPGMPNVSNDLSQKLYATMEKHKEVFFVIRLIAGPAANSLPP
IVDPDPLIPCDLMDGRDAFLTLARDKHLEFSSLRRAQWSTMCMLVELHTQSQDGSDYKDDDDK

dCas9-p300 PHD-HAT: amino acid sequence; Streptococcus pyogenes Cas9 (D10A, H840A), Nuclear Localization Sequence, Human p300 aa 1243-1664, 1 X “Flag” Epitope

MDKKYSIGLAIGTNSVGWAVITDEYKVPSKKFKVLGNTDRHSIKKNLIGALLFDSGETAEATRLKRTARRR
YTRRKNRICYLQEIFSNEMAKVDDSFFHRLEESFLVEEDKKHERHPIFGNIVDEVAYHEKYPTIYHLRKKLV
DSTDKADLRLIYLALAHMIKFRGHFLIEGDLNPDNSDVDKLFIQLVQTYNQLFEENPINASGVDAKAILSARL
SKSRRLENLIAQLPGEKKNGLFGNLIALSLGLTPNFKSNFDLAEDAKLQLSKDTYDDDLDNLLAQIGDQYA
DLFLAAKNLSDAILLSDILRVNTEITKAPLSASMIKRYDEHHQDLTLLKALVRQQLPEKYKEIFFDQSKNGYA
GYIDGGASQEEFYKFIKPILEKMDGTEELLVKLNREDLLRKQRTFDNGSIPHQIHLGELHAILRRQEDFYPF
LKDNREKIEKILTFRIPYYVGPLARGNSRFAWMTRKSEETITPWNFEEVVDKGASAQSFIERMTNFDKNLP
NEKVLPKHSLLYEYFTVYNELTKVKYVTEGMRKPAFLSGEQKKAIVDLLFKTNRKVTVKQLKEDYFKKIEC
FDSVEISGVEDRFNASLGTYHDLLKIIKDKDFLDNEENEDILEDIVLTLTLFEDREMIEERLKTYAHLFDDKV
MKQLKRRRYTGWGRLSRKLINGIRDKQSGKTILDFLKSDGFANRNFMQLIHDDSLTFKEDIQKAQVSGQG
DSLHEHIANLAGSPAIKKGILQTVKVVDELVKVMGRHKPENIVIEMARENQTTQKGQKNSRERMKRIEEGI
KELGSQILKEHPVENTQLQNEKLYLYYLQNGRDMYVDQELDINRLSDYDVDAIVPQSFLKDDSIDNKVLTR
SDKNRGKSDNVPSEEVVKKMKNYWRQLLNAKLITQRKFDNLTKAERGGLSELDKAGFIKRQLVETRQITK
HVAQILDSRMNTKYDENDKLIREVKVITLKSKLVSDFRKDFQFYKVREINNYHHAHDAYLNAVVGTALIKKY
PKLESEFVYGDYKVYDVRKMIAKSEQEIGKATAKYFFYSNIMNFFKTEITLANGEIRKRPLIETNGETGEIV
WDKGRDFATVRKVLSMPQVNIVKKTEVQTGGFSKESILPKRNSDKLIARKKDWDPKKYGGFDSPTVAYS
VLVVAKVEKGKSKKLKSVKELLGITIMERSSFEKNPIDFLEAKGYKEVKKDLIIKLPKYSLFELENGRKRMLA
SAGELQKGNELALPSKYVNFLYLASHYEKLKGSPEDNEQKQLFVEQHKHYLDEIIEQISEFSKRVILADANL
DKVLSAYNKHRDKPIREQAENIIHLFTLTNLGAPAAFKYFDTTIDRKRYTSTKEVLDATLIHQSITGLYETRID
LSQLGGDKRPAATKKAGQAKKKKGSLFVECTECGRKMHQICVLHHEIIWPAGFVCDGCLKKSARTRKEN
KFSAKRLPSTRLGTFLENRVNDFLRRQNHPESGEVTVRVVHASDKTVEVKPGMKARFVDSGEMAESFPY
RTKALFAFEEIDGVDLCFFGMHVQEYGSDCPPPNQRRVYISYLDSVHFFRPKCLRTAVYHEILIGYLEYVK
KLGYTTGHIWACPPSEGDDYIFHCHPPDQKIPKPKRLQEWYKKMLDKAVSERIVHDYKDIFKQATEDRLT
SAKELPYFEGDFWPNVLEESIKELEQEEEERKREENTSNESTDVTKGDSKNAKKKNNKKTSKNKSSLSR

GNKKKPGMPNVSNDLSQKLYATMEKHKEVFFVIRLIAGPAANSLPPIVDPDPLIPCDLMDGRDAFLTLARD
KHLEFSSLRRAQWSTMCMLVELHTQSQDGSDYKDDDDK

dCas9-p300 RING-PHD-HAT: amino acid sequence; Streptococcus pyogenes Cas9 (D10A, H840A), Nuclear Localization Sequence, Human p300 aa 1155-1664, 1 X "Flag" Epitope

MDKKYSIGLAIGTNSVGWAVITDEYKVPSKKFKVLGNTDRHSIKKNLIGALLFDSGETAEATRLKRTARRR
YTRRKNRICYLQEIFSNEMAKVDDSFFHRLEESFLVEEDKKHERHPIFGNIVDEVAYHEKYPTIYHLRKKLV
DSTDKADLRLIYLALAHMIKFRGHFLIEGDLNPDNSDVDKLFIQLVQTYNQLFEENPINASGVDAKAILSARL
SKSRRLENLIAQLPGEKKNGLFGNLIALSLGLTPNFKSNFDLAEDAKLQLSKDTYDDDLDNLLAQIGDQYA
DLFLAAKNLSDAILLSDILRVNTEITKAPLSASMIKRYDEHHQDLTLLKALVRQQLPEKYKEIFFDQSKNGYA
GYIDGGASQEEFYKFIKPILEKMDGTEELLVKLNREDLLRKQRTFDNGSIPHQIHLGELHAILRRQEDFYPF
LKDNREKIEKILTFRIPYYVGPLARGNSRFAWMTRKSEETITPWNFEEVVDKGASAQSFIERMTNFDKNLP
NEKVLPKHSLLYEYFTVYNELTKVKYVTEGMRKPAFLSGEQKKAIVDLLFKTNRKVTVKQLKEDYFKKIEC
FDSVEISGVEDRFNASLGTYHDLLKIIKDKDFLDNEENEDILEDIVLTLTLFEDREMIEERLKTYAHLFDDKV
MKQLKRRRYTGWGRLSRKLINGIRDKQSGKTILDFLKSDGFANRNFMQLIHDDSLTFKEDIQKAQVSGQG
DSLHEHIANLAGSPAIKKGILQTVKVVDELVKVMGRHKPENIVIEMARENQTTQKGQKNSRERMKRIEEGI
KELGSQILKEHPVENTQLQNEKLYLYYLQNGRDMYVDQELDINRLSDYDVDAIVPQSFLKDDSIDNKVLTR
SDKNRGKSDNVPSEEVVKKMKNYWRQLLNAKLITQRKFDNLTKAERGGLSELDKAGFIKRQLVETRQITK
HVAQILDSRMNTKYDENDKLIREVKVITLKSKLVSDFRKDFQFYKVREINNYHHAHDAYLNAVVGTALIKKY
PKLESEFVYGDYKVYDVRKMIAKSEQEIGKATAKYFFYSNIMNFFKTEITLANGEIRKRPLIETNGETGEIV
WDKGRDFATVRKVLSMPQVNIVKKTEVQTGGFSKESILPKRNSDKLIARKKDWDPKKYGGFDSPTVAYS
VLVVAKVEKGKSKKLKSVKELLGITIMERSSFEKNPIDFLEAKGYKEVKKDLIIKLPKYSLFELENGRKRMLA
SAGELQKGNELALPSKYVNFLYLASHYEKLKGSPEDNEQKQLFVEQHKHYLDEIIEQISEFSKRVILADANL
DKVLSAYNKHRDKPIREQAENIIHLFTLTNLGAPAAFKYFDTTIDRKRYTSTKEVLDATLIHQSITGLYETRID
LSQLGGDKRPAATKKAGQAKKKKGSPVMQSLGYCCGRKLEFSPQTLCCYGKQLCTIPRDATYYSYQNR
YHFCEKCFNEIQGESVSLGDDPSQPQTTINKEQFSKRKNDTLDPELFVECTECGRKMHQICVLHHEIIWP
AGFVCDGCLKKSARTRKENKFSAKRLPSTRLGTFLENRVNDFLRRQNHPESGEVTVRVVHASDKTVEVK
PGMKARFVDSGEMAESFPYRTKALFAFEEIDGVDLCFFGMHVQEYGSDCPPPNQRRVYISYLDSVHFFR
PKCLRTAVYHEILIGYLEYVKKLGYTTGHIWACPPSEGDDYIFHCHPPDQKIPKPKRLQEWYKKMLDKAVS
ERIVHDYKDIFKQATEDRLTSAKELPYFEGDFWPNVLEESIKELEQEEEERKREENTSNESTDVTKGDSK
NAKKKNNKKTSKNKSSLSRGNKKKPGMPNVSNDLSQKLYATMEKHKEVFFVIRLIAGPAANSLPPIVDPD
PLIPCDLMDGRDAFLTLARDKHLEFSSLRRAQWSTMCMLVELHTQSQDGSDYKDDDDK

dCas9-p300 FL: amino acid sequence; Streptococcus pyogenes Cas9 (D10A, H840A), Nuclear Localization Sequence, Human p300 aa 2-2414, 1 X "Flag" Epitope

MDKKYSIGLAIGTNSVGWAVITDEYKVPSKKFKVLGNTDRHSIKKNLIGALLFDSGETAEATRLKRTARRR
YTRRKNRICYLQEIFSNEMAKVDDSFFHRLEESFLVEEDKKHERHPIFGNIVDEVAYHEKYPTIYHLRKKLV
DSTDKADLRLIYLALAHMIKFRGHFLIEGDLNPDNSDVDKLFIQLVQTYNQLFEENPINASGVDAKAILSARL
SKSRRLENLIAQLPGEKKNGLFGNLIALSLGLTPNFKSNFDLAEDAKLQLSKDTYDDDLDNLLAQIGDQYA
DLFLAAKNLSDAILLSDILRVNTEITKAPLSASMIKRYDEHHQDLTLLKALVRQQLPEKYKEIFFDQSKNGYA
GYIDGGASQEEFYKFIKPILEKMDGTEELLVKLNREDLLRKQRTFDNGSIPHQIHLGELHAILRRQEDFYPF
LKDNREKIEKILTFRIPYYVGPLARGNSRFAWMTRKSEETITPWNFEEVVDKGASAQSFIERMTNFDKNLP
NEKVLPKHSLLYEYFTVYNELTKVKYVTEGMRKPAFLSGEQKKAIVDLLFKTNRKVTVKQLKEDYFKKIEC
FDSVEISGVEDRFNASLGTYHDLLKIIKDKDFLDNEENEDILEDIVLTLTLFEDREMIEERLKTYAHLFDDKV
MKQLKRRRYTGWGRLSRKLINGIRDKQSGKTILDFLKSDGFANRNFMQLIHDDSLTFKEDIQKAQVSGQG
DSLHEHIANLAGSPAIKKGILQTVKVVDELVKVMGRHKPENIVIEMARENQTTQKGQKNSRERMKRIEEGI
KELGSQILKEHPVENTQLQNEKLYLYYLQNGRDMYVDQELDINRLSDYDVDAIVPQSFLKDDSIDNKVLTR
SDKNRGKSDNVPSEEVVKKMKNYWRQLLNAKLITQRKFDNLTKAERGGLSELDKAGFIKRQLVETRQITK
HVAQILDSRMNTKYDENDKLIREVKVITLKSKLVSDFRKDFQFYKVREINNYHHAHDAYLNAVVGTALIKKY
PKLESEFVYGDYKVYDVRKMIAKSEQEIGKATAKYFFYSNIMNFFKTEITLANGEIRKRPLIETNGETGEIV
WDKGRDFATVRKVLSMPQVNIVKKTEVQTGGFSKESILPKRNSDKLIARKKDWDPKKYGGFDSPTVAYS
VLVVAKVEKGKSKKLKSVKELLGITIMERSSFEKNPIDFLEAKGYKEVKKDLIIKLPKYSLFELENGRKRMLA
SAGELQKGNELALPSKYVNFLYLASHYEKLKGSPEDNEQKQLFVEQHKHYLDEIIEQISEFSKRVILADANL
DKVLSAYNKHRDKPIREQAENIIHLFTLTNLGAPAAFKYFDTTIDRKRYTSTKEVLDATLIHQSITGLYETRID
LSQLGGDKRPAATKKAGQAKKKKGSAENVVEPGPPSAKRPKLSSPALSASASDGTDFGSLFDLEHDLPD
ELINSTELGLTNGGDINQLQTSLGMVQDAASKHKQLSELLRSGSSPNLNMGVGGPGQVMASQAQQSSP
GLGLINSMVKSPMTQAGLTSPNMGMGTSGPNQGPTQSTGMMNSPVNQPAMGMNTGMNAGMNPGML
AAGNGQGIMPNQVMNGSIGAGRGRQNMQYPNPGMGSAGNLLTEPLQQGSPQMGGQTGLRGPQPLKM
GMMNNPNPYGSPYTQNPGQQIGASGLGLQIQTKTVLSNNLSPFAMDKKAVPGGGMPNMGQQPAPQVQ
QPGLVTPVAQGMGSGAHTADPEKRKLIQQQLVLLLHAHKCQRREQANGEVRQCNLPHCRTMKNVLNH
MTHCQSGKSCQVAHCASSRQIISHWKNCTRHDCPVCLPLKNAGDKRNQQPILTGAPVGLGNPSSLGVG
QQSAPNLSTVSQIDPSSIERAYAALGLPYQVNQMPTQPQVQAKNQQNQQPGQSPQGMRPMSNMSASP
MGVNGGVGVQTPSLLSDSMLHSAINSQNPMMSENASVPSMGPMPTAAQPSTTGIRKQWHEDITQDLRN
HLVHKLVQAIFPTPDPAALKDRRMENLVAYARKVEGDMYESANNRAEYYHLLAEKIYKIQKELEEKRRTRL
QKQNMLPNAAGMVPVSMNPGPNMGQPQPGMTSNGPLPDPSMIRGSVPNQMMPRITPQSGLNQFGQM
SMAQPPIVPRQTPPLQHHGQLAQPGALNPPMGYGPRMQQPSNQGQFLPQTQFPSQGMNVTNIPLAPS
SGQAPVSQAQMSSSSCPVNSPIMPPGSQGSHIHCPQLPQPALHQNSPSPVPSRTPTPHHTPPSIGAQQP
PATTIPAPVPTPPAMPPGPQSQALHPPPRQTPTPPTTQLPQQVQPSLPAAPSADQPQQQPRSQQSTAAS
VPTPTAPLLPPQPATPLSQPAVSIEGQVSNPPSTSSTEVNSQAIAEKQPSQEVKMEAKMEVDQPEPADTQ
PEDISESKVEDCKMESTETEERSTELKTEIKEEEDQPSTSATQSSPAPGQSKKKIFKPEELRQALMPTLEA
LYRQDPESLPFRQPVDPQLLGIPDYFDIVKSPMDLSTIKRKLDTGQYQEPWQYVDDIWLMFNNAWLYNR
KTSRVYKYCSKLSEVFEQEIDPVMQSLGYCCGRKLEFSPQTLCCYGKQLCTIPRDATYYSYQNRYHFCE

KCFNEIQGESVSLGDDPSQPQTTINKEQFSKRKNDTLDPELFVECTECGRKMHQICVLHHEIIWPAGFVC
DGCLKKSARTRKENKFSAKRLPSTRLGTFLENRVNDFLRRQNHPESGEVTVRVVHASDKTVEVKPGMKA
RFVDSGEMAESFPYRTKALFAFEEIDGVDLCFFGMHVQEYGSDCPPPNQRRVYISYLDSVHFFRPKCLR
TAVYHEILIGYLEYVKKLGYTTGHIWACPPSEGDDYIFHCHPPDQKIPKPKRLQEWYKKMLDKAVSERIVH
DYKDIFKQATEDRLTSAKELPYFEGDFWPNVLEESIKELEQEEEERKREENTSNESTDVTKGDSKNAKKK
NNKKTSKNKSSLSRGNKKKPGMPNVSNDLSQKLYATMEKHKEVFFVIRLIAGPAANSLPPIVDPDPLIPCD
LMDGRDAFLTLARDKHLEFSSLRRAQWSTMCMLVELHTQSQDRFVYTCNECKHHVETRWHCTVCEDY
DLCITCYNTKNHDHKMEKLGLGLDDESNNQQAAATQSPGDSRRLSIQRCIQSLVHACQCRNANCSLPSC
QKMKRVVQHTKGCKRKTNGGCPICKQLIALCCYHAKHCQENKCPVPFCLNIKQKLRQQQLQHRLQQAQ
MLRRRMASMQRTGVVGQQQGLPSPTPATPTTPTGQQPTTPQTPQPTSQPQPTPPNSMPPYLPRTQAA
GPVSQGKAAGQVTPPTPPQTAQPPLPGPPPAAVEMAMQIQRAAETQRQMAHVQIFQRPIQHQMPPMTP
MAPMGMNPPPMTRGPSGHLEPGMGPTGMQQQPPWSQGGLPQPQQLQSGMPRPAMMSVAQHGQPL
NMAPQPGLGQVGISPLKPGTVSQQALQNLLRTLRSPSSPLQQQQVLSILHANPQLLAAFIKQRAAKYANS
NPQPIPGQPGMPQGQPGLQPPTMPGQQGVHSNPAMQNMNPMQAGVQRAGLPQQQPQQQLQPPMG
GMSPQAQQMNMNHNTMPSQFRDILRRQQMMQQQQQQGAGPGIGPGMANHNQFQQPQGVGYPPQQ
QQRMQHHMQQMQQGNMGQIGQLPQALGAEAGASLQAYQQRLLQQQMGSPVQPNPMSPQQHMLPNQ
AQSPHLQGQQIPNSLSNQVRSPQPVPSPRPQSQPPHSSPSPRMQPQPSPHHVSPQTSSPHPGLVAAQ
ANPMEQGHFASPDQNSMLSQLASNPGMANLHGASATDLGLSTDNSDLNSNLSQSTLDIHGSDYKDDDD
K

dCas9-CBP HAT: amino acid sequence; Streptococcus pyogenes Cas9 (D10A, H840A), Nuclear Localization Sequence, Human CBP aa 1323-1701, 1 X “Flag” Epitope

MDKKYSIGLAIGTNSVGWAVITDEYKVPSKKFKVLGNTDRHSIKKNLIGALLFDSGETAEATRLKRTARRR
YTRRKNRICYLQEIFSNEMAKVDDSFFHRLEESFLVEEDKKHERHPIFGNIVDEVAYHEKYPTIYHLRKKLV
DSTDKADLRLIYLALAHMIKFRGHFLIEGDLNPDNSDVDKLFIQLVQTYNQLFEENPINASGVDAKAILSARL
SKSRRLENLIAQLPGEKKNGLFGNLIALSLGLTPNFKSNFDLAEDAKLQLSKDTYDDDLDNLLAQIGDQYA
DLFLAAKNLSDAILLSDILRVNTEITKAPLSASMIKRYDEHHQDLTLLKALVRQQLPEKYKEIFFDQSKNGYA
GYIDGGASQEEFYKFIKPILEKMDGTEELLVKLNREDLLRKQRTFDNGSIPHQIHLGELHAILRRQEDFYPF
LKDNREKIEKILTFRIPYYVGPLARGNSRFAWMTRKSEETITPWNFEEVVDKGASAQSFIERMTNFDKNLP
NEKVLPKHSLLYEYFTVYNELTKVKYVTEGMRKPAFLSGEQKKAIVDLLFKTNRKVTVKQLKEDYFKKIEC
FDSVEISGVEDRFNASLGTYHDLLKIIKDKDFLDNEENEDILEDIVLTLTLFEDREMIEERLKTYAHLFDDKV
MKQLKRRRYTGWGRLSRKLINGIRDKQSGKTILDFLKSDGFANRNFMQLIHDDSLTFKEDIQKAQVSGQG
DSLHEHIANLAGSPAIKKGILQTVKVVDELVKVMGRHKPENIVIEMARENQTTQKGQKNSRERMKRIEEGI
KELGSQILKEHPVENTQLQNEKLYLYYLQNGRDMYVDQELDINRLSDYDVDAIVPQSFLKDDSIDNKVLTR
SDKNRGKSDNVPSEEVVKKMKNYWRQLLNAKLITQRKFDNLTKAERGGLSELDKAGFIKRQLVETRQITK
HVAQILDSRMNTKYDENDKLIREVKVITLKSKLVSDFRKDFQFYKVREINNYHHAHDAYLNAVVGTALIKKY
PKLESEFVYGDYKVYDVRKMIAKSEQEIGKATAKYFFYSNIMNFFKTEITLANGEIRKRPLIETNGETGEIV

WDKGRDFATVRKVLSMPQVNIVKKTEVQTGGFSKESILPKRNSDKLIARKKDWDPKKYGGFDSPTVAYS
VLVVAKVEKGKSKKLKSVKELLGITIMERSSFEKNPIDFLEAKGYKEVKKDLIIKLPKYSLFELENGRKRMLA
SAGELQKGNELALPSKYVNFLYLASHYEKLKGSPEDNEQKQLFVEQHKHYLDEIIEQISEFSKRVILADANL
DKVLSAYNKHRDKPIREQAENIIHLFTLTNLGAPAAFKYFDTTIDRKRYTSTKEVLDATLIHQSITGLYETRID
LSQLGGDKRPAATKKAGQAKKKKGSKFSAKRLQTTRLGNHLEDRVNKFLRRQNHPEAGEVFVRVVASS
DKTVEVKPGMKSRFVDSGEMSESFPYRTKALFAFEEIDGVDVCFFGMHVQEYGSDCPPPNTRRVYISYL
DSIHFFRPRCLRTAVYHEILIGYLEYVKKLGYVTGHIWACPPSEGDDYIFHCHPPDQKIPKPKRLQEWYKK
MLDKAFAERIIHDYKDIFKQATEDRLTSAKELPYFEGDFWPNVLEESIKELEQEEEERKKEESTAASETTE
GSQGDSKNAKKKNNKKTNKNKSSISRANKKKPSMPNVSNDLSQKLYATMEKHKEVFFVIHLHAGPVINTL
PPIVDPDPLLSCDLMDGRDAFLTLARDKHWEFSSLRRSKWSTLCMLVELHTQGQGSDYKDDDDK

dCas9-CBP PHD-HAT: amino acid sequence; Streptococcus pyogenes Cas9 (D10A, H840A)， Nuclear Localization Sequence, Human CBP aa 1279-1701, 1 X “Flag”

MDKKYSIGLAIGTNSVGWAVITDEYKVPSKKFKVLGNTDRHSIKKNLIGALLFDSGETAEATRLKRTARRR
YTRRKNRICYLQEIFSNEMAKVDDSFFHRLEESFLVEEDKKHERHPIFGNIVDEVAYHEKYPTIYHLRKKLV
DSTDKADLRLIYLALAHMIKFRGHFLIEGDLNPDNSDVDKLFIQLVQTYNQLFEENPINASGVDAKAILSARL
SKSRRLENLIAQLPGEKKNGLFGNLIALSLGLTPNFKSNFDLAEDAKLQLSKDTYDDDLDNLLAQIGDQYA
DLFLAAKNLSDAILLSDILRVNTEITKAPLSASMIKRYDEHHQDLTLLKALVRQQLPEKYKEIFFDQSKNGYA
GYIDGGASQEEFYKFIKPILEKMDGTEELLVKLNREDLLRKQRTFDNGSIPHQIHLGELHAILRRQEDFYPF
LKDNREKIEKILTFRIPYYVGPLARGNSRFAWMTRKSEETITPWNFEEVVDKGASAQSFIERMTNFDKNLP
NEKVLPKHSLLYEYFTVYNELTKVKYVTEGMRKPAFLSGEQKKAIVDLLFKTNRKVTVKQLKEDYFKKIEC
FDSVEISGVEDRFNASLGTYHDLLKIIKDKDFLDNEENEDILEDIVLTLTLFEDREMIEERLKTYAHLFDDKV
MKQLKRRRYTGWGRLSRKLINGIRDKQSGKTILDFLKSDGFANRNFMQLIHDDSLTFKEDIQKAQVSGQG
DSLHEHIANLAGSPAIKKGILQTVKVVDELVKVMGRHKPENIVIEMARENQTTQKGQKNSRERMKRIEEGI
KELGSQILKEHPVENTQLQNEKLYLYYLQNGRDMYVDQELDINRLSDYDVDAIVPQSFLKDDSIDNKVLTR
SDKNRGKSDNVPSEEVVKKMKNYWRQLLNAKLITQRKFDNLTKAERGGLSELDKAGFIKRQLVETRQITK
HVAQILDSRMNTKYDENDKLIREVKVITLKSKLVSDFRKDFQFYKVREINNYHHAHDAYLNAVVGTALIKKY
PKLESEFVYGDYKVYDVRKMIAKSEQEIGKATAKYFFYSNIMNFFKTEITLANGEIRKRPLIETNGETGEIV
WDKGRDFATVRKVLSMPQVNIVKKTEVQTGGFSKESILPKRNSDKLIARKKDWDPKKYGGFDSPTVAYS
VLVVAKVEKGKSKKLKSVKELLGITIMERSSFEKNPIDFLEAKGYKEVKKDLIIKLPKYSLFELENGRKRMLA
SAGELQKGNELALPSKYVNFLYLASHYEKLKGSPEDNEQKQLFVEQHKHYLDEIIEQISEFSKRVILADANL
DKVLSAYNKHRDKPIREQAENIIHLFTLTNLGAPAAFKYFDTTIDRKRYTSTKEVLDATLIHQSITGLYETRID
LSQLGGDKRPAATKKAGQAKKKKGSPFVDCKECGRKMHQICVLHYDIIWPSGFVCDNCLKKTGRPRKEN
KFSAKRLQTTRLGNHLEDRVNKFLRRQNHPEAGEVFVRVVASSDKTVEVKPGMKSRFVDSGEMSESFP
YRTKALFAFEEIDGVDVCFFGMHVQEYGSDCPPPNTRRVYISYLDSIHFFRPRCLRTAVYHEILIGYLEYVK
KLGYVTGHIWACPPSEGDDYIFHCHPPDQKIPKPKRLQEWYKKMLDKAFAERIIHDYKDIFKQATEDRLTS

AKELPYFEGDFWPNVLEESIKELEQEEEERKKEESTAASETTEGSQGDSKNAKKKNNKKTNKNKSSISRA
NKKKPSMPNVSNDLSQKLYATMEKHKEVFFVIHLHAGPVINTLPPIVDPDPLLSCDLMDGRDAFLTLARDK
HWEFSSLRRSKWSTLCMLVELHTQGQGSDYKDDDDK

dCas9-CBP RING-PHD-HAT: amino acid sequence; Streptococcus pyogenes Cas9 (D10A, H840A), Nuclear Localization Sequence, Human CBP aa 1191-1701, 1 X “Flag”

MDKKYSIGLAIGTNSVGWAVITDEYKVPSKKFKVLGNTDRHSIKKNLIGALLFDSGETAEATRLKRTARRR
YTRRKNRICYLQEIFSNEMAKVDDSFFHRLEESFLVEEDKKHERHPIFGNIVDEVAYHEKYPTIYHLRKKLV
DSTDKADLRLIYLALAHMIKFRGHFLIEGDLNPDNSDVDKLFIQLVQTYNQLFEENPINASGVDAKAILSARL
SKSRRLENLIAQLPGEKKNGLFGNLIALSLGLTPNFKSNFDLAEDAKLQLSKDTYDDDLDNLLAQIGDQYA
DLFLAAKNLSDAILLSDILRVNTEITKAPLSASMIKRYDEHHQDLTLLKALVRQQLPEKYKEIFFDQSKNGYA
GYIDGGASQEEFYKFIKPILEKMDGTEELLVKLNREDLLRKQRTFDNGSIPHQIHLGELHAILRRQEDFYPF
LKDNREKIEKILTFRIPYYVGPLARGNSRFAWMTRKSEETITPWNFEEVVDKGASAQSFIERMTNFDKNLP
NEKVLPKHSLLYEYFTVYNELTKVKYVTEGMRKPAFLSGEQKKAIVDLLFKTNRKVTVKQLKEDYFKKIEC
FDSVEISGVEDRFNASLGTYHDLLKIIKDKDFLDNEENEDILEDIVLTLTLFEDREMIEERLKTYAHLFDDKV
MKQLKRRRYTGWGRLSRKLINGIRDKQSGKTILDFLKSDGFANRNFMQLIHDDSLTFKEDIQKAQVSGQG
DSLHEHIANLAGSPAIKKGILQTVKVVDELVKVMGRHKPENIVIEMARENQTTQKGQKNSRERMKRIEEGI
KELGSQILKEHPVENTQLQNEKLYLYYLQNGRDMYVDQELDINRLSDYDVDAIVPQSFLKDDSIDNKVLTR
SDKNRGKSDNVPSEEVVKKMKNYWRQLLNAKLITQRKFDNLTKAERGGLSELDKAGFIKRQLVETRQITK
HVAQILDSRMNTKYDENDKLIREVKVITLKSKLVSDFRKDFQFYKVREINNYHHAHDAYLNAVVGTALIKKY
PKLESEFVYGDYKVYDVRKMIAKSEQEIGKATAKYFFYSNIMNFFKTEITLANGEIRKRPLIETNGETGEIV
WDKGRDFATVRKVLSMPQVNIVKKTEVQTGGFSKESILPKRNSDKLIARKKDWDPKKYGGFDSPTVAYS
VLVVAKVEKGKSKKLKSVKELLGITIMERSSFEKNPIDFLEAKGYKEVKKDLIIKLPKYSLFELENGRKRMLA
SAGELQKGNELALPSKYVNFLYLASHYEKLKGSPEDNEQKQLFVEQHKHYLDEIIEQISEFSKRVILADANL
DKVLSAYNKHRDKPIREQAENIIHLFTLTNLGAPAAFKYFDTTIDRKRYTSTKEVLDATLIHQSITGLYETRID
LSQLGGDKRPAATKKAGQAKKKKGSPVMQSLGYCCGRKYEFSPQTLCCYGKQLCTIPRDAAYYSYQNR
YHFCEKCFTEIQGENVTLGDDPSQPQTTISKDQFEKKKNDTLDPEPFVDCKECGRKMHQICVLHYDIIWP
SGFVCDNCLKKTGRPRKENKFSAKRLQTTRLGNHLEDRVNKFLRRQNHPEAGEVFVRVVASSDKTVEVK
PGMKSRFVDSGEMSESFPYRTKALFAFEEIDGVDVCFFGMHVQEYGSDCPPPNTRRVYISYLDSIHFFRP
RCLRTAVYHEILIGYLEYVKKLGYVTGHIWACPPSEGDDYIFHCHPPDQKIPKPKRLQEWYKKMLDKAFAE
RIIHDYKDIFKQATEDRLTSAKELPYFEGDFWPNVLEESIKELEQEEEERKKEESTAASETTEGSQGDSKN
AKKKNNKKTNKNKSSISRANKKKPSMPNVSNDLSQKLYATMEKHKEVFFVIHLHAGPVINTLPPIVDPDPL
LSCDLMDGRDAFLTLARDKHWEFSSLRRSKWSTLCMLVELHTQGQGSDYKDDDDK

dCas9-CBP FL: amino acid sequence; Streptococcus pyogenes Cas9 (D10A, H840A), Nuclear Localization Sequence, Human CBP aa 2-2443, 1 X “Flag”

MDKKYSIGLAIGTNSVGWAVITDEYKVPSKKFKVLGNTDRHSIKKNLIGALLFDSGETAEATRLKRTARRR
YTRRKNRICYLQEIFSNEMAKVDDSFFHRLEESFLVEEDKKHERHPIFGNIVDEVAYHEKYPTIYHLRKKLV
DSTDKADLRLIYLALAHMIKFRGHFLIEGDLNPDNSDVDKLFIQLVQTYNQLFEENPINASGVDAKAILSARL
SKSRRLENLIAQLPGEKKNGLFGNLIALSLGLTPNFKSNFDLAEDAKLQLSKDTYDDDLDNLLAQIGDQYA
DLFLAAKNLSDAILLSDILRVNTEITKAPLSASMIKRYDEHHQDLTLLKALVRQQLPEKYKEIFFDQSKNGYA
GYIDGGASQEEFYKFIKPILEKMDGTEELLVKLNREDLLRKQRTFDNGSIPHQIHLGELHAILRRQEDFYPF
LKDNREKIEKILTFRIPYYVGPLARGNSRFAWMTRKSEETITPWNFEEVVDKGASAQSFIERMTNFDKNLP
NEKVLPKHSLLYEYFTVYNELTKVKYVTEGMRKPAFLSGEQKKAIVDLLFKTNRKVTVKQLKEDYFKKIEC
FDSVEISGVEDRFNASLGTYHDLLKIIKDKDFLDNEENEDILEDIVLTLTLFEDREMIEERLKTYAHLFDDKV
MKQLKRRRYTGWGRLSRKLINGIRDKQSGKTILDFLKSDGFANRNFMQLIHDDSLTFKEDIQKAQVSGQG
DSLHEHIANLAGSPAIKKGILQTVKVVDELVKVMGRHKPENIVIEMARENQTTQKGQKNSRERMKRIEEGI
KELGSQILKEHPVENTQLQNEKLYLYYLQNGRDMYVDQELDINRLSDYDVDAIVPQSFLKDDSIDNKVLTR
SDKNRGKSDNVPSEEVVKKMKNYWRQLLNAKLITQRKFDNLTKAERGGLSELDKAGFIKRQLVETRQITK
HVAQILDSRMNTKYDENDKLIREVKVITLKSKLVSDFRKDFQFYKVREINNYHHAHDAYLNAVVGTALIKKY
PKLESEFVYGDYKVYDVRKMIAKSEQEIGKATAKYFFYSNIMNFFKTEITLANGEIRKRPLIETNGETGEIV
WDKGRDFATVRKVLSMPQVNIVKKTEVQTGGFSKESILPKRNSDKLIARKKDWDPKKYGGFDSPTVAYS
VLVVAKVEKGKSKKLKSVKELLGITIMERSSFEKNPIDFLEAKGYKEVKKDLIIKLPKYSLFELENGRKRMLA
SAGELQKGNELALPSKYVNFLYLASHYEKLKGSPEDNEQKQLFVEQHKHYLDEIIEQISEFSKRVILADANL
DKVLSAYNKHRDKPIREQAENIIHLFTLTNLGAPAAFKYFDTTIDRKRYTSTKEVLDATLIHQSITGLYETRID
LSQLGGDKRPAATKKAGQAKKKKGSAENLLDGPPNPKRAKLSSPGFSANDSTDFGSLFDLENDLPDELIP
NGGELGLLNSGNLVPDAASKHKQLSELLRGGSGSSINPGIGNVSASSPVQQGLGGQAQGQPNSANMAS
LSAMGKSPLSQGDSSAPSLPKQAASTSGPTPAASQALNPQAQKQVGLATSSPATSQTGPGICMNANFN
QTHPGLLNSNSGHSLINQASQGQAQVMNGSLGAAGRGRGAGMPYPTPAMQGASSSVLAETLTQVSPQ
MTGHAGLNTAQAGGMAKMGITGNTSPFGQPFSQAGGQPMGATGVNPQLASKQSMVNSLPTFPTDIKNT
SVTNVPNMSQMQTSVGIVPTQAIATGPTADPEKRKLIQQQLVLLLHAHKCQRREQANGEVRACSLPHCR
TMKNVLNHMTHCQAGKACQVAHCASSRQIISHWKNCTRHDCPVCLPLKNASDKRNQQTILGSPASGIQN
TIGSVGTGQQNATSLSNPNPIDPSSMQRAYAALGLPYMNQPQTQLQPQVPGQQPAQPQTHQQMRTLNP
LGNNPMNIPAGGITTDQQPPNLISESALPTSLGATNPLMNDGSNSGNIGTLSTIPTAAPPSSTGVRKGWHE
HVTQDLRSHLVHKLVQAIFPTPDPAALKDRRMENLVAYAKKVEGDMYESANSRDEYYHLLAEKIYKIQKEL
EEKRRSRLHKQGILGNQPALPAPGAQPPVIPQAQPVRPPNGPLSLPVNRMQVSQGMNSFNPMSLGNVQ
LPQAPMGPRAASPMNHSVQMNSMGSVPGMAISPSRMPQPPNMMGAHTNNMMAQAPAQSQFLPQNQF
PSSSGAMSVGMGQPPAQTGVSQGQVPGAALPNPLNMLGPQASQLPCPPVTQSPLHPTPPPASTAAGM
PSLQHTTPPGMTPPQPAAPTQPSTPVSSSGQTPTPTPGSVPSATQTQSTPTVQAAAQAQVTPQPQTPV
QPPSVATPQSSQQQPTPVHAQPPGTPLSQAAASIDNRVPTPSSVASAETNSQQPGPDVPVLEMKTETQ
AEDTEPDPGESKGEPRSEMMEEDLQGASQVKEETDIAEQKSEPMEVDEKKPEVKVEVKEEEESSSNGT
ASQSTSPSQPRKKIFKPEELRQALMPTLEALYRQDPESLPFRQPVDPQLLGIPDYFDIVKNPMDLSTIKRK
LDTGQYQEPWQYVDDVWLMFNNAWLYNRKTSRVYKFCSKLAEVFEQEIDPVMQSLGYCCGRKYEFSP

QTLCCYGKQLCTIPRDAAYYSYQNRYHFCEKCFTEIQGENVTLGDDPSQPQTTISKDQFEKKKNDTLDPE
PFVDCKECGRKMHQICVLHYDIIWPSGFVCDNCLKKTGRPRKENKFSAKRLQTTRLGNHLEDRVNKFLR
RQNHPEAGEVFVRVVASSDKTVEVKPGMKSRFVDSGEMSESFPYRTKALFAFEEIDGVDVCFFGMHVQ
EYGSDCPPPNTRRVYISYLDSIHFFRPRCLRTAVYHEILIGYLEYVKKLGYVTGHIWACPPSEGDDYIFHCH
PPDQKIPKPKRLQEWYKKMLDKAFAERIIHDYKDIFKQATEDRLTSAKELPYFEGDFWPNVLEESIKELEQ
EEEERKKEESTAASETTEGSQGDSKNAKKKNNKKTNKNKSSISRANKKKPSMPNVSNDLSQKLYATMEK
HKEVFFVIHLHAGPVINTLPPIVDPDPLLSCDLMDGRDAFLTLARDKHWEFSSLRRSKWSTLCMLVELHTQ
GQDRFVYTCNECKHHVETRWHCTVCEDYDLCINCYNTKSHAHKMVKWGLGLDDEGSSQGEPQSKSPQ
ESRRLSIQRCIQSLVHACQCRNANCSLPSCQKMKRVVQHTKGCKRKTNGGCPVCKQLIALCCYHAKHCQ
ENKCPVPFCLNIKHKLRQQQIQHRLQQAQLMRRRMATMNTRNVPQQSLPSPTSAPPGTPTQQPSTPQT
PQPPAQPQPSPVSMSPAGFPSVARTQPPTTVSTGKPTSQVPAPPPPAQPPPAAVEAARQIEREAQQQQ
HLYRVNINNSMPPGRTGMGTPGSQMAPVSLNVPRPNQVSGPVMPSMPPGQWQQAPLPQQQPMPGLP
RPVISMQAQAAVAGPRMPSVQPPRSISPSALQDLLRTLKSPSSPQQQQQVLNILKSNPQLMAAFIKQRTA
KYVANQPGMQPQPGLQSQPGMQPQPGMHQQPSLQNLNAMQAGVPRPGVPPQQQAMGGLNPQGQAL
NIMNPGHNPNMASMNPQYREMLRRQLLQQQQQQQQQQQQQQQQQQGSAGMAGGMAGHGQFQQP
QGPGGYPPAMQQQQRMQQHLPLQGSSMGQMAAQMGQLGQMGQPGLGADSTPNIQQALQQRILQQQ
QMKQQIGSPGQPNPMSPQQHMLSGQPQASHLPGQQIATSLSNQVRSPAPVQSPRPQSQPPHSSPSPRI
QPQPSPHHVSPQTGSPHPGLAVTMASSIDQGHLGNPEQSAMLPQLNTPSRSALSSELSLVGDTTGDTLE
KFVEGLGSDYKDDDDK

dCas9-GCN5 core: amino acid sequence; Streptococcus pyogenes Cas9 (D10A, H840A), Nuclear Localization Sequence, Human GCN5 aa 473-677, 1 X “Flag”

MDKKYSIGLAIGTNSVGWAVITDEYKVPSKKFKVLGNTDRHSIKKNLIGALLFDSGETAEATRLKRTARRR
YTRRKNRICYLQEIFSNEMAKVDDSFFHRLEESFLVEEDKKHERHPIFGNIVDEVAYHEKYPTIYHLRKKLV
DSTDKADLRLIYLALAHMIKFRGHFLIEGDLNPDNSDVDKLFIQLVQTYNQLFEENPINASGVDAKAILSARL
SKSRRLENLIAQLPGEKKNGLFGNLIALSLGLTPNFKSNFDLAEDAKLQLSKDTYDDDLDNLLAQIGDQYA
DLFLAAKNLSDAILLSDILRVNTEITKAPLSASMIKRYDEHHQDLTLLKALVRQQLPEKYKEIFFDQSKNGYA
GYIDGGASQEEFYKFIKPILEKMDGTEELLVKLNREDLLRKQRTFDNGSIPHQIHLGELHAILRRQEDFYPF
LKDNREKIEKILTFRIPYYVGPLARGNSRFAWMTRKSEETITPWNFEEVVDKGASAQSFIERMTNFDKNLP
NEKVLPKHSLLYEYFTVYNELTKVKYVTEGMRKPAFLSGEQKKAIVDLLFKTNRKVTVKQLKEDYFKKIEC
FDSVEISGVEDRFNASLGTYHDLLKIIKDKDFLDNEENEDILEDIVLTLTLFEDREMIEERLKTYAHLFDDKV
MKQLKRRRYTGWGRLSRKLINGIRDKQSGKTILDFLKSDGFANRNFMQLIHDDSLTFKEDIQKAQVSGQG
DSLHEHIANLAGSPAIKKGILQTVKVVDELVKVMGRHKPENIVIEMARENQTTQKGQKNSRERMKRIEEGI
KELGSQILKEHPVENTQLQNEKLYLYYLQNGRDMYVDQELDINRLSDYDVDAIVPQSFLKDDSIDNKVLTR
SDKNRGKSDNVPSEEVVKKMKNYWRQLLNAKLITQRKFDNLTKAERGGLSELDKAGFIKRQLVETRQITK
HVAQILDSRMNTKYDENDKLIREVKVITLKSKLVSDFRKDFQFYKVREINNYHHAHDAYLNAVVGTALIKKY

PKLESEFVYGDYKVYDVRKMIAKSEQEIGKATAKYFFYSNIMNFFKTEITLANGEIRKRPLIETNGETGEIV
WDKGRDFATVRKVLSMPQVNIVKKTEVQTGGFSKESILPKRNSDKLIARKKDWDPKKYGGFDSPTVAYS
VLVVAKVEKGKSKKLKSVKELLGITIMERSSFEKNPIDFLEAKGYKEVKKDLIIKLPKYSLFELENGRKRMLA
SAGELQKGNELALPSKYVNFLYLASHYEKLKGSPEDNEQKQLFVEQHKHYLDEIIEQISEFSKRVILADANL
DKVLSAYNKHRDKPIREQAENIIHLFTLTNLGAPAAFKYFDTTIDRKRYTSTKEVLDATLIHQSITGLYETRID
LSQLGGDKRPAATKKAGQAKKKKGSLGPETSLLSANAARDETARLEERRGIIEFHVIGNSLTPKANRRVLL
WLVGLQNVFSHQLPRMPKEYIARLVFDPKHKTLALIKDGRVIGGICFRMFPTQGFTEIVFCAVTSNEQVKG
YGTHLMNHLKEYHIKHNILYFLTYADEYAIGYFKKQGFSKDIKVPKSRYLGYIKDYEGATLMECELNPRIPY
TELSHIIKKQKEIIKGSDYKDDDDK

dCas9-GCN5 FL: amino acid sequence; Streptococcus pyogenes Cas9 (D10A, H840A), Nuclear Localization Sequence, Human GCN5 aa 1-837, 1 X “Flag”

MDKKYSIGLAIGTNSVGWAVITDEYKVPSKKFKVLGNTDRHSIKKNLIGALLFDSGETAEATRLKRTARRR
YTRRKNRICYLQEIFSNEMAKVDDSFFHRLEESFLVEEDKKHERHPIFGNIVDEVAYHEKYPTIYHLRKKLV
DSTDKADLRLIYLALAHMIKFRGHFLIEGDLNPDNSDVDKLFIQLVQTYNQLFEENPINASGVDAKAILSARL
SKSRRLENLIAQLPGEKKNGLFGNLIALSLGLTPNFKSNFDLAEDAKLQLSKDTYDDDLDNLLAQIGDQYA
DLFLAAKNLSDAILLSDILRVNTEITKAPLSASMIKRYDEHHQDLTLLKALVRQQLPEKYKEIFFDQSKNGYA
GYIDGGASQEEFYKFIKPILEKMDGTEELLVKLNREDLLRKQRTFDNGSIPHQIHLGELHAILRRQEDFYPF
LKDNREKIEKILTFRIPYYVGPLARGNSRFAWMTRKSEETITPWNFEEVVDKGASAQSFIERMTNFDKNLP
NEKVLPKHSLLYEYFTVYNELTKVKYVTEGMRKPAFLSGEQKKAIVDLLFKTNRKVTVKQLKEDYFKKIEC
FDSVEISGVEDRFNASLGTYHDLLKIIKDKDFLDNEENEDILEDIVLTLTLFEDREMIEERLKTYAHLFDDKV
MKQLKRRRYTGWGRLSRKLINGIRDKQSGKTILDFLKSDGFANRNFMQLIHDDSLTFKEDIQKAQVSGQG
DSLHEHIANLAGSPAIKKGILQTVKVVDELVKVMGRHKPENIVIEMARENQTTQKGQKNSRERMKRIEEGI
KELGSQILKEHPVENTQLQNEKLYLYYLQNGRDMYVDQELDINRLSDYDVDAIVPQSFLKDDSIDNKVLTR
SDKNRGKSDNVPSEEVVKKMKNYWRQLLNAKLITQRKFDNLTKAERGGLSELDKAGFIKRQLVETRQITK
HVAQILDSRMNTKYDENDKLIREVKVITLKSKLVSDFRKDFQFYKVREINNYHHAHDAYLNAVVGTALIKKY
PKLESEFVYGDYKVYDVRKMIAKSEQEIGKATAKYFFYSNIMNFFKTEITLANGEIRKRPLIETNGETGEIV
WDKGRDFATVRKVLSMPQVNIVKKTEVQTGGFSKESILPKRNSDKLIARKKDWDPKKYGGFDSPTVAYS
VLVVAKVEKGKSKKLKSVKELLGITIMERSSFEKNPIDFLEAKGYKEVKKDLIIKLPKYSLFELENGRKRMLA
SAGELQKGNELALPSKYVNFLYLASHYEKLKGSPEDNEQKQLFVEQHKHYLDEIIEQISEFSKRVILADANL
DKVLSAYNKHRDKPIREQAENIIHLFTLTNLGAPAAFKYFDTTIDRKRYTSTKEVLDATLIHQSITGLYETRID
LSQLGGDKRPAATKKAGQAKKKKGSMAEPSQAPTPAPAAQPRPLQSPAPAPTPTPAPSPASAPIPTPTP
APAPAPAAAPAGSTGTGGPGVGSGGAGSGGDPARPGLSQQQRASQRKAQVRGLPRAKKLEKLGVFSA
CKANETCKCNGWKNPKPPTAPRMDLQQPAANLSELCRSCEHPLADHVSHLENVSEDEINRLLGMVVDV
ENLFMSVHKEEDTDTKQVYFYLFKLLRKCILQMTRPVVEGSLGSPPFEKPNIEQGVLNFVQYKFSHLAPR
ERQTMFELSKMFLLCLNYWKLETPAQFRQRSQAEDVATYKVNYTRWLCYCHVPQSCDSLPRYETTHVF
GRSLLRSIFTVTRRQLLEKFRVEKDKLVPEKRTLILTHFPKFLSMLEEEIYGANSPIWESGFTMPPSEGTQL

VPRPASVSAAVVPSTPIFSPSMGGGSNSSLSLDSAGAEPMPGEKRTLPENLTLEDAKRLRVMGDIPMELV
NEVMLTITDPAAMLGPETSLLSANAARDETARLEERRGIIEFHVIGNSLTPKANRRVLLWLVGLQNVFSHQ
LPRMPKEYIARLVFDPKHKTLALIKDGRVIGGICFRMFPTQGFTEIVFCAVTSNEQVKGYGTHLMNHLKEY
HIKHNILYFLTYADEYAIGYFKKQGFSKDIKVPKSRYLGYIKDYEGATLMECELNPRIPYTELSHIIKKQKEIIK
KLIERKQAQIRKVYPGLSCFKEGVRQIPVESVPGIRETGWKPLGKEKGKELKDPDQLYTTLKNLLAQIKSH
PSAWPFMEPVKKSEAPDYYEVIRFPIDLKTMTERLRSRYYVTRKLFVADLQRVIANCREYNPPDSEYCRC
ASALEKFFYFKLKEGGLIDKGSDYKDDDDK

GCN5 core-dCas9-p300 core: amino acid sequence; Human GCN5 aa 473-677, SV-40, Streptococcus pyogenes Cas9 (D10A, H840A), Nuclear Localization Sequence, Human p300 aa 1048-1664, 1 X “Flag”

MLGPETSLLSANAARDETARLEERRGIIEFHVIGNSLTPKANRRVLLWLVGLQNVFSHQLPRMPKEYIARL
VFDPKHKTLALIKDGRVIGGICFRMFPTQGFTEIVFCAVTSNEQVKGYGTHLMNHLKEYHIKHNILYFLTYA
DEYAIGYFKKQGFSKDIKVPKSRYLGYIKDYEGATLMECELNPRIPYTELSHIIKKQKEIIKGSPKKKRKVGS
SAATMDKKYSIGLAIGTNSVGWAVITDEYKVPSKKFKVLGNTDRHSIKKNLIGALLFDSGETAEATRLKRTA
RRRYTRRKNRICYLQEIFSNEMAKVDDSFFHRLEESFLVEEDKKHERHPIFGNIVDEVAYHEKYPTIYHLR
KKLVDSTDKADLRLIYLALAHMIKFRGHFLIEGDLNPDNSDVDKLFIQLVQTYNQLFEENPINASGVDAKAIL
SARLSKSRRLENLIAQLPGEKKNGLFGNLIALSLGLTPNFKSNFDLAEDAKLQLSKDTYDDDLDNLLAQIGD
QYADLFLAAKNLSDAILLSDILRVNTEITKAPLSASMIKRYDEHHQDLTLLKALVRQQLPEKYKEIFFDQSKN
GYAGYIDGGASQEEFYKFIKPILEKMDGTEELLVKLNREDLLRKQRTFDNGSIPHQIHLGELHAILRRQEDF
YPFLKDNREKIEKILTFRIPYYVGPLARGNSRFAWMTRKSEETITPWNFEEVVDKGASAQSFIERMTNFDK
NLPNEKVLPKHSLLYEYFTVYNELTKVKYVTEGMRKPAFLSGEQKKAIVDLLFKTNRKVTVKQLKEDYFKK
IECFDSVEISGVEDRFNASLGTYHDLLKIIKDKDFLDNEENEDILEDIVLTLTLFEDREMIEERLKTYAHLFDD
KVMKQLKRRRYTGWGRLSRKLINGIRDKQSGKTILDFLKSDGFANRNFMQLIHDDSLTFKEDIQKAQVSG
QGDSLHEHIANLAGSPAIKKGILQTVKVVDELVKVMGRHKPENIVIEMARENQTTQKGQKNSRERMKRIEE
GIKELGSQILKEHPVENTQLQNEKLYLYYLQNGRDMYVDQELDINRLSDYDVDAIVPQSFLKDDSIDNKVL
TRSDKNRGKSDNVPSEEVVKKMKNYWRQLLNAKLITQRKFDNLTKAERGGLSELDKAGFIKRQLVETRQI
TKHVAQILDSRMNTKYDENDKLIREVKVITLKSKLVSDFRKDFQFYKVREINNYHHAHDAYLNAVVGTALIK
KYPKLESEFVYGDYKVYDVRKMIAKSEQEIGKATAKYFFYSNIMNFFKTEITLANGEIRKRPLIETNGETGEI
VWDKGRDFATVRKVLSMPQVNIVKKTEVQTGGFSKESILPKRNSDKLIARKKDWDPKKYGGFDSPTVAY
SVLVVAKVEKGKSKKLKSVKELLGITIMERSSFEKNPIDFLEAKGYKEVKKDLIIKLPKYSLFELENGRKRML
ASAGELQKGNELALPSKYVNFLYLASHYEKLKGSPEDNEQKQLFVEQHKHYLDEIIEQISEFSKRVILADAN
LDKVLSAYNKHRDKPIREQAENIIHLFTLTNLGAPAAFKYFDTTIDRKRYTSTKEVLDATLIHQSITGLYETRI
DLSQLGGDKRPAATKKAGQAKKKKGSIFKPEELRQALMPTLEALYRQDPESLPFRQPVDPQLLGIPDYFD
IVKSPMDLSTIKRKLDTGQYQEPWQYVDDIWLMFNNAWLYNRKTSRVYKYCSKLSEVFEQEIDPVMQSL
GYCCGRKLEFSPQTLCCYGKQLCTIPRDATYYSYQNRYHFCEKCFNEIQGESVSLGDDPSQPQTTINKE
QFSKRKNDTLDPELFVECTECGRKMHQICVLHHEIIWPAGFVCDGCLKKSARTRKENKFSAKRLPSTRLG

TFLENRVNDFLRRQNHPESGEVTVRVVHASDKTVEVKPGMKARFVDSGEMAESFPYRTKALFAFEEIDG
VDLCFFGMHVQEYGSDCPPPNQRRVYISYLDSVHFFRPKCLRTAVYHEILIGYLEYVKKLGYTTGHIWAC
PPSEGDDYIFHCHPPDQKIPKPKRLQEWYKKMLDKAVSERIVHDYKDIFKQATEDRLTSAKELPYFEGDF
WPNVLEESIKELEQEEEERKREENTSNESTDVTKGDSKNAKKKNNKKTSKNKSSLSRGNKKKPGMPNVS
NDLSQKLYATMEKHKEVFFVIRLIAGPAANSLPPIVDPDPLIPCDLMDGRDAFLTLARDKHLEFSSLRRAQ
WSTMCMLVELHTQSQDGSDYKDDDDK

GCN5 FL-dCas9-p300 core: amino acid sequence; Human GCN5 aa 1-837, SV-40, Streptococcus pyogenes Cas9 (D10A, H840A), Nuclear Localization Sequence, Human p300 aa 1048-1664, 1 X “Flag”

MAEPSQAPTPAPAAQPRPLQSPAPAPTPTPAPSPASAPIPTPTPAPAPAPAAAPAGSTGTGGPGVGSGG
AGSGGDPARPGLSQQQRASQRKAQVRGLPRAKKLEKLGVFSACKANETCKCNGWKNPKPPTAPRMDL
QQPAANLSELCRSCEHPLADHVSHLENVSEDEINRLLGMVVDVENLFMSVHKEEDTDTKQVYFYLFKLLR
KCILQMTRPVVEGSLGSPPFEKPNIEQGVLNFVQYKFSHLAPRERQTMFELSKMFLLCLNYWKLETPAQF
RQRSQAEDVATYKVNYTRWLCYCHVPQSCDSLPRYETTHVFGRSLLRSIFTVTRRQLLEKFRVEKDKLV
PEKRTLILTHFPKFLSMLEEEIYGANSPIWESGFTMPPSEGTQLVPRPASVSAAVVPSTPIFSPSMGGGSN
SSLSLDSAGAEPMPGEKRTLPENLTLEDAKRLRVMGDIPMELVNEVMLTITDPAAMLGPETSLLSANAAR
DETARLEERRGIIEFHVIGNSLTPKANRRVLLWLVGLQNVFSHQLPRMPKEYIARLVFDPKHKTLALIKDGR
VIGGICFRMFPTQGFTEIVFCAVTSNEQVKGYGTHLMNHLKEYHIKHNILYFLTYADEYAIGYFKKQGFSKD
IKVPKSRYLGYIKDYEGATLMECELNPRIPYTELSHIIKKQKEIIKKLIERKQAQIRKVYPGLSCFKEGVRQIP
VESVPGIRETGWKPLGKEKGKELKDPDQLYTTLKNLLAQIKSHPSAWPFMEPVKKSEAPDYYEVIRFPIDL
KTMTERLRSRYYVTRKLFVADLQRVIANCREYNPPDSEYCRCASALEKFFYFKLKEGGLIDKGSPKKKRK
VGSSAATMDKKYSIGLAIGTNSVGWAVITDEYKVPSKKFKVLGNTDRHSIKKNLIGALLFDSGETAEATRLK
RTARRRYTRRKNRICYLQEIFSNEMAKVDDSFFHRLEESFLVEEDKKHERHPIFGNIVDEVAYHEKYPTIY
HLRKKLVDSTDKADLRLIYLALAHMIKFRGHFLIEGDLNPDNSDVDKLFIQLVQTYNQLFEENPINASGVDA
KAILSARLSKSRRLENLIAQLPGEKKNGLFGNLIALSLGLTPNFKSNFDLAEDAKLQLSKDTYDDDLDNLLA
QIGDQYADLFLAAKNLSDAILLSDILRVNTEITKAPLSASMIKRYDEHHQDLTLLKALVRQQLPEKYKEIFFD
QSKNGYAGYIDGGASQEEFYKFIKPILEKMDGTEELLVKLNREDLLRKQRTFDNGSIPHQIHLGELHAILRR
QEDFYPFLKDNREKIEKILTFRIPYYVGPLARGNSRFAWMTRKSEETITPWNFEEVVDKGASAQSFIERMT
NFDKNLPNEKVLPKHSLLYEYFTVYNELTKVKYVTEGMRKPAFLSGEQKKAIVDLLFKTNRKVTVKQLKED
YFKKIECFDSVEISGVEDRFNASLGTYHDLLKIIKDKDFLDNEENEDILEDIVLTLTLFEDREMIEERLKTYAH
LFDDKVMKQLKRRRYTGWGRLSRKLINGIRDKQSGKTILDFLKSDGFANRNFMQLIHDDSLTFKEDIQKA
QVSGQGDSLHEHIANLAGSPAIKKGILQTVKVVDELVKVMGRHKPENIVIEMARENQTTQKGQKNSRERM
KRIEEGIKELGSQILKEHPVENTQLQNEKLYLYYLQNGRDMYVDQELDINRLSDYDVDAIVPQSFLKDDSID
NKVLTRSDKNRGKSDNVPSEEVVKKMKNYWRQLLNAKLITQRKFDNLTKAERGGLSELDKAGFIKRQLV
ETRQITKHVAQILDSRMNTKYDENDKLIREVKVITLKSKLVSDFRKDFQFYKVREINNYHHAHDAYLNAVV
GTALIKKYPKLESEFVYGDYKVYDVRKMIAKSEQEIGKATAKYFFYSNIMNFFKTEITLANGEIRKRPLIETN

GETGEIVWDKGRDFATVRKVLSMPQVNIVKKTEVQTGGFSKESILPKRNSDKLIARKKDWDPKKYGGFDS
PTVAYSVLVVAKVEKGKSKKLKSVKELLGITIMERSSFEKNPIDFLEAKGYKEVKKDLIIKLPKYSLFELENG
RKRMLASAGELQKGNELALPSKYVNFLYLASHYEKLKGSPEDNEQKQLFVEQHKHYLDEIIEQISEFSKRV
ILADANLDKVLSAYNKHRDKPIREQAENIIHLFTLTNLGAPAAFKYFDTTIDRKRYTSTKEVLDATLIHQSITG
LYETRIDLSQLGGDKRPAATKKAGQAKKKKGSIFKPEELRQALMPTLEALYRQDPESLPFRQPVDPQLLGI
PDYFDIVKSPMDLSTIKRKLDTGQYQEPWQYVDDIWLMFNNAWLYNRKTSRVYKYCSKLSEVFEQEIDPV
MQSLGYCCGRKLEFSPQTLCCYGKQLCTIPRDATYYSYQNRYHFCEKCFNEIQGESVSLGDDPSQPQTT
INKEQFSKRKNDTLDPELFVECTECGRKMHQICVLHHEIIWPAGFVCDGCLKKSARTRKENKFSAKRLPS
TRLGTFLENRVNDFLRRQNHPESGEVTVRVVHASDKTVEVKPGMKARFVDSGEMAESFPYRTKALFAFE
EIDGVDLCFFGMHVQEYGSDCPPPNQRRVYISYLDSVHFFRPKCLRTAVYHEILIGYLEYVKKLGYTTGHI
WACPPSEGDDYIFHCHPPDQKIPKPKRLQEWYKKMLDKAVSERIVHDYKDIFKQATEDRLTSAKELPYFE
GDFWPNVLEESIKELEQEEEERKREENTSNESTDVTKGDSKNAKKKNNKKTSKNKSSLSRGNKKKPGMP
NVSNDLSQKLYATMEKHKEVFFVIRLIAGPAANSLPPIVDPDPLIPCDLMDGRDAFLTLARDKHLEFSSLRR
AQWSTMCMLVELHTQSQDGSDYKDDDDK

GCN5 core-dCas9-CBP core: amino acid sequence; Human GCN5 aa 473-677, SV-40, Streptococcus pyogenes Cas9 (D10A, H840A), Nuclear Localization Sequence, Human CBP aa 1087-1701, 1 X “Flag”

MLGPETSLLSANAARDETARLEERRGIIEFHVIGNSLTPKANRRVLLWLVGLQNVFSHQLPRMPKEYIARL
VFDPKHKTLALIKDGRVIGGICFRMFPTQGFTEIVFCAVTSNEQVKGYGTHLMNHLKEYHIKHNILYFLTYA
DEYAIGYFKKQGFSKDIKVPKSRYLGYIKDYEGATLMECELNPRIPYTELSHIIKKQKEIIKGSPKKKRKVGS
SAATMDKKYSIGLAIGTNSVGWAVITDEYKVPSKKFKVLGNTDRHSIKKNLIGALLFDSGETAEATRLKRTA
RRRYTRRKNRICYLQEIFSNEMAKVDDSFFHRLEESFLVEEDKKHERHPIFGNIVDEVAYHEKYPTIYHLR
KKLVDSTDKADLRLIYLALAHMIKFRGHFLIEGDLNPDNSDVDKLFIQLVQTYNQLFEENPINASGVDAKAIL
SARLSKSRRLENLIAQLPGEKKNGLFGNLIALSLGLTPNFKSNFDLAEDAKLQLSKDTYDDDLDNLLAQIGD
QYADLFLAAKNLSDAILLSDILRVNTEITKAPLSASMIKRYDEHHQDLTLLKALVRQQLPEKYKEIFFDQSKN
GYAGYIDGGASQEEFYKFIKPILEKMDGTEELLVKLNREDLLRKQRTFDNGSIPHQIHLGELHAILRRQEDF
YPFLKDNREKIEKILTFRIPYYVGPLARGNSRFAWMTRKSEETITPWNFEEVVDKGASAQSFIERMTNFDK
NLPNEKVLPKHSLLYEYFTVYNELTKVKYVTEGMRKPAFLSGEQKKAIVDLLFKTNRKVTVKQLKEDYFKK
IECFDSVEISGVEDRFNASLGTYHDLLKIIKDKDFLDNEENEDILEDIVLTLTLFEDREMIEERLKTYAHLFDD
KVMKQLKRRRYTGWGRLSRKLINGIRDKQSGKTILDFLKSDGFANRNFMQLIHDDSLTFKEDIQKAQVSG
QGDSLHEHIANLAGSPAIKKGILQTVKVVDELVKVMGRHKPENIVIEMARENQTTQKGQKNSRERMKRIEE
GIKELGSQILKEHPVENTQLQNEKLYLYYLQNGRDMYVDQELDINRLSDYDVDAIVPQSFLKDDSIDNKVL
TRSDKNRGKSDNVPSEEVVKKMKNYWRQLLNAKLITQRKFDNLTKAERGGLSELDKAGFIKRQLVETRQI
TKHVAQILDSRMNTKYDENDKLIREVKVITLKSKLVSDFRKDFQFYKVREINNYHHAHDAYLNAVVGTALIK
KYPKLESEFVYGDYKVYDVRKMIAKSEQEIGKATAKYFFYSNIMNFFKTEITLANGEIRKRPLIETNGETGEI
VWDKGRDFATVRKVLSMPQVNIVKKTEVQTGGFSKESILPKRNSDKLIARKKDWDPKKYGGFDSPTVAY

SVLVVAKVEKGKSKKLKSVKELLGITIMERSSFEKNPIDFLEAKGYKEVKKDLIIKLPKYSLFELENGRKRML ASAGELQKGNELALPSKYVNFLYLASHYEKLKGSPEDNEQKQLFVEQHKHYLDEIIEQISEFSKRVILADAN LDKVLSAYNKHRDKPIREQAENIIHLFTLTNLGAPAAFKYFDTTIDRKRYTSTKEVLDATLIHQSITGLYETRI DLSQLGGDKRPAATKKAGQAKKKKGSIFKPEELRQALMPTLEALYRQDPESLPFRQPVDPQLLGIPDYFD IVKNPMDLSTIKRKLDTGQYQEPWQYVDDVWLMFNNAWLYNRKTSRVYKFCSKLAEVFEQEIDPVMQSL GYCCGRKYEFSPQTLCCYGKQLCTIPRDAAYYSYQNRYHFCEKCFTEIQGENVTLGDDPSQPQTTISKD QFEKKKNDTLDPEPFVDCKECGRKMHQICVLHYDIIWPSGFVCDNCLKKTGRPRKENKFSAKRLQTTRL GNHLEDRVNKFLRRQNHPEAGEVFVRVVASSDKTVEVKPGMKSRFVDSGEMSESFPYRTKALFAFEEID GVDVCFFGMHVQEYGSDCPPPNTRRVYISYLDSIHFFRPRCLRTAVYHEILIGYLEYVKKLGYVTGHIWAC PPSEGDDYIFHCHPPDQKIPKPKRLQEWYKKMLDKAFAERIIHDYKDIFKQATEDRLTSAKELPYFEGDFW PNVLEESIKELEQEEEERKKEESTAASETTEGSQGDSKNAKKKNNKKTNKNKSSISRANKKKPSMPNVSN DLSQKLYATMEKHKEVFFVIHLHAGPVINTLPPIVDPDPLLSCDLMDGRDAFLTLARDKHWEFSSLRRSK WSTLCMLVELHTQGQDGSDYKDDDDK

GCN5 FL-dCas9-CBP core: amino acid sequence; Human GCN5 aa 1-837, SV-40, Streptococcus pyogenes Cas9 (D10A, H840A), Nuclear Localization Sequence, Human CBP aa 1087-1701, 1 X “Flag”

MAEPSQAPTPAPAAQPRPLQSPAPAPTPTPAPSPASAPIPTPTPAPAPAPAAAPAGSTGTGGPGVGSGG AGSGGDPARPGLSQQQRASQRKAQVRGLPRAKKLEKLGVFSACKANETCKCNGWKNPKPPTAPRMDL QQPAANLSELCRSCEHPLADHVSHLENVSEDEINRLLGMVVDVENLFMSVHKEEDTDTKQVYFYLFKLLR KCILQMTRPVVEGSLGSPPFEKPNIEQGVLNFVQYKFSHLAPRERQTMFELSKMFLLCLNYWKLETPAQF RQRSQAEDVATYKVNYTRWLCYCHVPQSCDSLPRYETTHVFGRSLLRSIFTVTRRQLLEKFRVEKDKLV PEKRTLILTHFPKFLSMLEEEIYGANSPIWESGFTMPPSEGTQLVPRPASVSAAVVPSTPIFSPSMGGGSN SSLSLDSAGAEPMPGEKRTLPENLTLEDAKRLRVMGDIPMELVNEVMLTITDPAAMLGPETSLLSANAAR DETARLEERRGIIEFHVIGNSLTPKANRRVLLWLVGLQNVFSHQLPRMPKEYIARLVFDPKHKTLALIKDGR VIGGICFRMFPTQGFTEIVFCAVTSNEQVKGYGTHLMNHLKEYHIKHNILYFLTYADEYAIGYFKKQGFSKD IKVPKSRYLGYIKDYEGATLMECELNPRIPYTELSHIIKKQKEIIKKLIERKQAQIRKVYPGLSCFKEGVRQIP VESVPGIRETGWKPLGKEKGKELKDPDQLYTTLKNLLAQIKSHPSAWPFMEPVKKSEAPDYYEVIRFPIDL KTMTERLRSRYYVTRKLFVADLQRVIANCREYNPPDSEYCRCASALEKFFYFKLKEGGLIDKGSPKKKRK VGSSAATMDKKYSIGLAIGTNSVGWAVITDEYKVPSKKFKVLGNTDRHSIKKNLIGALLFDSGETAEATRLK RTARRRYTRRKNRICYLQEIFSNEMAKVDDSFFHRLEESFLVEEDKKHERHPIFGNIVDEVAYHEKYPTIY HLRKKLVDSTDKADLRLIYLALAHMIKFRGHFLIEGDLNPDNSDVDKLFIQLVQTYNQLFEENPINASGVDA KAILSARLSKSRRLENLIAQLPGEKKNGLFGNLIALSLGLTPNFKSNFDLAEDAKLQLSKDTYDDDLDNLLA QIGDQYADLFLAAKNLSDAILLSDILRVNTEITKAPLSASMIKRYDEHHQDLTLLKALVRQQLPEKYKEIFFD QSKNGYAGYIDGGASQEEFYKFIKPILEKMDGTEELLVKLNREDLLRKQRTFDNGSIPHQIHLGELHAILRR QEDFYPFLKDNREKIEKILTFRIPYYVGPLARGNSRFAWMTRKSEETITPWNFEEVVDKGASAQSFIERMT NFDKNLPNEKVLPKHSLLYEYFTVYNELTKVKYVTEGMRKPAFLSGEQKKAIVDLLFKTNRKVTVKQLKED

YFKKIECFDSVEISGVEDRFNASLGTYHDLLKIIKDKDFLDNEENEDILEDIVLTLTLFEDREMIEERLKTYAH
LFDDKVMKQLKRRRYTGWGRLSRKLINGIRDKQSGKTILDFLKSDGFANRNFMQLIHDDSLTFKEDIQKA
QVSGQGDSLHEHIANLAGSPAIKKGILQTVKVVDELVKVMGRHKPENIVIEMARENQTTQKGQKNSRERM
KRIEEGIKELGSQILKEHPVENTQLQNEKLYLYYLQNGRDMYVDQELDINRLSDYDVDAIVPQSFLKDDSID
NKVLTRSDKNRGKSDNVPSEEVVKKMKNYWRQLLNAKLITQRKFDNLTKAERGGLSELDKAGFIKRQLV
ETRQITKHVAQILDSRMNTKYDENDKLIREVKVITLKSKLVSDFRKDFQFYKVREINNYHHAHDAYLNAVV
GTALIKKYPKLESEFVYGDYKVYDVRKMIAKSEQEIGKATAKYFFYSNIMNFFKTEITLANGEIRKRPLIETN
GETGEIVWDKGRDFATVRKVLSMPQVNIVKKTEVQTGGFSKESILPKRNSDKLIARKKDWDPKKYGGFDS
PTVAYSVLVVAKVEKGKSKKLKSVKELLGITIMERSSFEKNPIDFLEAKGYKEVKKDLIIKLPKYSLFELENG
RKRMLASAGELQKGNELALPSKYVNFLYLASHYEKLKGSPEDNEQKQLFVEQHKHYLDEIIEQISEFSKRV
ILADANLDKVLSAYNKHRDKPIREQAENIIHLFTLTNLGAPAAFKYFDTTIDRKRYTSTKEVLDATLIHQSITG
LYETRIDLSQLGGDKRPAATKKAGQAKKKKGSIFKPEELRQALMPTLEALYRQDPESLPFRQPVDPQLLGI
PDYFDIVKNPMDLSTIKRKLDTGQYQEPWQYVDDVWLMFNNAWLYNRKTSRVYKFCSKLAEVFEQEIDP
VMQSLGYCCGRKYEFSPQTLCCYGKQLCTIPRDAAYYSYQNRYHFCEKCFTEIQGENVTLGDDPSQPQT
TISKDQFEKKKNDTLDPEPFVDCKECGRKMHQICVLHYDIIWPSGFVCDNCLKKTGRPRKENKFSAKRLQ
TTRLGNHLEDRVNKFLRRQNHPEAGEVFVRVVASSDKTVEVKPGMKSRFVDSGEMSESFPYRTKALFAF
EEIDGVDVCFFGMHVQEYGSDCPPPNTRRVYISYLDSIHFFRPRCLRTAVYHEILIGYLEYVKKLGYVTGHI
WACPPSEGDDYIFHCHPPDQKIPKPKRLQEWYKKMLDKAFAERIIHDYKDIFKQATEDRLTSAKELPYFEG
DFWPNVLEESIKELEQEEEERKKEESTAASETTEGSQGDSKNAKKKNNKKTNKNKSSISRANKKKPSMP
NVSNDLSQKLYATMEKHKEVFFVIHLHAGPVINTLPPIVDPDPLLSCDLMDGRDAFLTLARDKHWEFSSLR
RSKWSTLCMLVELHTQGQDGSDYKDDDDK

p300 core-dCas9-p300 core: amino acid sequence; Human p300 aa 1048-1664, SV-40, Streptococcus pyogenes Cas9 (D10A, H840A), Nuclear Localization Sequence, Human p300 aa 1048-1664, 1 X “Flag”

MIFKPEELRQALMPTLEALYRQDPESLPFRQPVDPQLLGIPDYFDIVKSPMDLSTIKRKLDTGQYQEPWQY
VDDIWLMFNNAWLYNRKTSRVYKYCSKLSEVFEQEIDPVMQSLGYCCGRKLEFSPQTLCCYGKQLCTIP
RDATYYSYQNRYHFCEKCFNEIQGESVSLGDDPSQPQTTINKEQFSKRKNDTLDPELFVECTECGRKMH
QICVLHHEIIWPAGFVCDGCLKKSARTRKENKFSAKRLPSTRLGTFLENRVNDFLRRQNHPESGEVTVRV
VHASDKTVEVKPGMKARFVDSGEMAESFPYRTKALFAFEEIDGVDLCFFGMHVQEYGSDCPPPNQRRV
YISYLDSVHFFRPKCLRTAVYHEILIGYLEYVKKLGYTTGHIWACPPSEGDDYIFHCHPPDQKIPKPKRLQE
WYKKMLDKAVSERIVHDYKDIFKQATEDRLTSAKELPYFEGDFWPNVLEESIKELEQEEEERKREENTSN
ESTDVTKGDSKNAKKKNNKKTSKNKSSLSRGNKKKPGMPNVSNDLSQKLYATMEKHKEVFFVIRLIAGPA
ANSLPPIVDPDPLIPCDLMDGRDAFLTLARDKHLEFSSLRRAQWSTMCMLVELHTQSQDGSPKKKRKVG
SSAATMDKKYSIGLAIGTNSVGWAVITDEYKVPSKKFKVLGNTDRHSIKKNLIGALLFDSGETAEATRLKRT
ARRRYTRRKNRICYLQEIFSNEMAKVDDSFFHRLEESFLVEEDKKHERHPIFGNIVDEVAYHEKYPTIYHL
RKKLVDSTDKADLRLIYLALAHMIKFRGHFLIEGDLNPDNSDVDKLFIQLVQTYNQLFEENPINASGVDAKAI

LSARLSKSRRLENLIAQLPGEKKNGLFGNLIALSLGLTPNFKSNFDLAEDAKLQLSKDTYDDDLDNLLAQIG
DQYADLFLAAKNLSDAILLSDILRVNTEITKAPLSASMIKRYDEHHQDLTLLKALVRQQLPEKYKEIFFDQSK
NGYAGYIDGGASQEEFYKFIKPILEKMDGTEELLVKLNREDLLRKQRTFDNGSIPHQIHLGELHAILRRQED
FYPFLKDNREKIEKILTFRIPYYVGPLARGNSRFAWMTRKSEETITPWNFEEVVDKGASAQSFIERMTNFD
KNLPNEKVLPKHSLLYEYFTVYNELTKVKYVTEGMRKPAFLSGEQKKAIVDLLFKTNRKVTVKQLKEDYFK
KIECFDSVEISGVEDRFNASLGTYHDLLKIIKDKDFLDNEENEDILEDIVLTLTLFEDREMIEERLKTYAHLFD
DKVMKQLKRRRYTGWGRLSRKLINGIRDKQSGKTILDFLKSDGFANRNFMQLIHDDSLTFKEDIQKAQVS
GQGDSLHEHIANLAGSPAIKKGILQTVKVVDELVKVMGRHKPENIVIEMARENQTTQKGQKNSRERMKRI
EEGIKELGSQILKEHPVENTQLQNEKLYLYYLQNGRDMYVDQELDINRLSDYDVDAIVPQSFLKDDSIDNK
VLTRSDKNRGKSDNVPSEEVVKKMKNYWRQLLNAKLITQRKFDNLTKAERGGLSELDKAGFIKRQLVETR
QITKHVAQILDSRMNTKYDENDKLIREVKVITLKSKLVSDFRKDFQFYKVREINNYHHAHDAYLNAVVGTAL
IKKYPKLESEFVYGDYKVYDVRKMIAKSEQEIGKATAKYFFYSNIMNFFKTEITLANGEIRKRPLIETNGETG
EIVWDKGRDFATVRKVLSMPQVNIVKKTEVQTGGFSKESILPKRNSDKLIARKKDWDPKKYGGFDSPTVA
YSVLVVAKVEKGKSKKLKSVKELLGITIMERSSFEKNPIDFLEAKGYKEVKKDLIIKLPKYSLFELENGRKRM
LASAGELQKGNELALPSKYVNFLYLASHYEKLKGSPEDNEQKQLFVEQHKHYLDEIIEQISEFSKRVILADA
NLDKVLSAYNKHRDKPIREQAENIIHLFTLTNLGAPAAFKYFDTTIDRKRYTSTKEVLDATLIHQSITGLYET
RIDLSQLGGDKRPAATKKAGQAKKKKGSIFKPEELRQALMPTLEALYRQDPESLPFRQPVDPQLLGIPDY
FDIVKSPMDLSTIKRKLDTGQYQEPWQYVDDIWLMFNNAWLYNRKTSRVYKYCSKLSEVFEQEIDPVMQ
SLGYCCGRKLEFSPQTLCCYGKQLCTIPRDATYYSYQNRYHFCEKCFNEIQGESVSLGDDPSQPQTTINK
EQFSKRKNDTLDPELFVECTECGRKMHQICVLHHEIIWPAGFVCDGCLKKSARTRKENKFSAKRLPSTRL
GTFLENRVNDFLRRQNHPESGEVTVRVVHASDKTVEVKPGMKARFVDSGEMAESFPYRTKALFAFEEID
GVDLCFFGMHVQEYGSDCPPPNQRRVYISYLDSVHFFRPKCLRTAVYHEILIGYLEYVKKLGYTTGHIWA
CPPSEGDDYIFHCHPPDQKIPKPKRLQEWYKKMLDKAVSERIVHDYKDIFKQATEDRLTSAKELPYFEGD
FWPNVLEESIKELEQEEEERKREENTSNESTDVTKGDSKNAKKKNNKKTSKNKSSLSRGNKKKPGMPNV
SNDLSQKLYATMEKHKEVFFVIRLIAGPAANSLPPIVDPDPLIPCDLMDGRDAFLTLARDKHLEFSSLRRAQ
WSTMCMLVELHTQSQDGSDYKDDDDK

CBP core-dCas9-p300 core: amino acid sequence; Human CBP aa 1087-1701, SV-40, Streptococcus pyogenes Cas9 (D10A, H840A), Nuclear Localization Sequence, Human p300 aa 1048-1664, 1 X “Flag”

MIFKPEELRQALMPTLEALYRQDPESLPFRQPVDPQLLGIPDYFDIVKNPMDLSTIKRKLDTGQYQEPWQ
YVDDVWLMFNNAWLYNRKTSRVYKFCSKLAEVFEQEIDPVMQSLGYCCGRKYEFSPQTLCCYGKQLCTI
PRDAAYYSYQNRYHFCEKCFTEIQGENVTLGDDPSQPQTTISKDQFEKKKNDTLDPEPFVDCKECGRKM
HQICVLHYDIIWPSGFVCDNCLKKTGRPRKENKFSAKRLQTTRLGNHLEDRVNKFLRRQNHPEAGEVFVR
VVASSDKTVEVKPGMKSRFVDSGEMSESFPYRTKALFAFEEIDGVDVCFFGMHVQEYGSDCPPPNTRR
VYISYLDSIHFFRPRCLRTAVYHEILIGYLEYVKKLGYVTGHIWACPPSEGDDYIFHCHPPDQKIPKPKRLQ
EWYKKMLDKAFAERIIHDYKDIFKQATEDRLTSAKELPYFEGDFWPNVLEESIKELEQEEEERKKEESTAA

SETTEGSQGDSKNAKKKNNKKTNKNKSSISRANKKKPSMPNVSNDLSQKLYATMEKHKEVFFVIHLHAG
PVINTLPPIVDPDPLLSCDLMDGRDAFLTLARDKHWEFSSLRRSKWSTLCMLVELHTQGQDGSPKKKRK
VGSSAATMDKKYSIGLAIGTNSVGWAVITDEYKVPSKKFKVLGNTDRHSIKKNLIGALLFDSGETAEATRLK
RTARRRYTRRKNRICYLQEIFSNEMAKVDDSFFHRLEESFLVEEDKKHERHPIFGNIVDEVAYHEKYPTIY
HLRKKLVDSTDKADLRLIYLALAHMIKFRGHFLIEGDLNPDNSDVDKLFIQLVQTYNQLFEENPINASGVDA
KAILSARLSKSRRLENLIAQLPGEKKNGLFGNLIALSLGLTPNFKSNFDLAEDAKLQLSKDTYDDDLDNLLA
QIGDQYADLFLAAKNLSDAILLSDILRVNTEITKAPLSASMIKRYDEHHQDLTLLKALVRQQLPEKYKEIFFD
QSKNGYAGYIDGGASQEEFYKFIKPILEKMDGTEELLVKLNREDLLRKQRTFDNGSIPHQIHLGELHAILRR
QEDFYPFLKDNREKIEKILTFRIPYYVGPLARGNSRFAWMTRKSEETITPWNFEEVVDKGASAQSFIERMT
NFDKNLPNEKVLPKHSLLYEYFTVYNELTKVKYVTEGMRKPAFLSGEQKKAIVDLLFKTNRKVTVKQLKED
YFKKIECFDSVEISGVEDRFNASLGTYHDLLKIIKDKDFLDNEENEDILEDIVLTLTLFEDREMIEERLKTYAH
LFDDKVMKQLKRRRYTGWGRLSRKLINGIRDKQSGKTILDFLKSDGFANRNFMQLIHDDSLTFKEDIQKA
QVSGQGDSLHEHIANLAGSPAIKKGILQTVKVVDELVKVMGRHKPENIVIEMARENQTTQKGQKNSRERM
KRIEEGIKELGSQILKEHPVENTQLQNEKLYLYYLQNGRDMYVDQELDINRLSDYDVDAIVPQSFLKDDSID
NKVLTRSDKNRGKSDNVPSEEVVKKMKNYWRQLLNAKLITQRKFDNLTKAERGGLSELDKAGFIKRQLV
ETRQITKHVAQILDSRMNTKYDENDKLIREVKVITLKSKLVSDFRKDFQFYKVREINNYHHAHDAYLNAVV
GTALIKKYPKLESEFVYGDYKVYDVRKMIAKSEQEIGKATAKYFFYSNIMNFFKTEITLANGEIRKRPLIETN
GETGEIVWDKGRDFATVRKVLSMPQVNIVKKTEVQTGGFSKESILPKRNSDKLIARKKDWDPKKYGGFDS
PTVAYSVLVVAKVEKGKSKKLKSVKELLGITIMERSSFEKNPIDFLEAKGYKEVKKDLIIKLPKYSLFELENG
RKRMLASAGELQKGNELALPSKYVNFLYLASHYEKLKGSPEDNEQKQLFVEQHKHYLDEIIEQISEFSKRV
ILADANLDKVLSAYNKHRDKPIREQAENIIHLFTLTNLGAPAAFKYFDTTIDRKRYTSTKEVLDATLIHQSITG
LYETRIDLSQLGGDKRPAATKKAGQAKKKKGSIFKPEELRQALMPTLEALYRQDPESLPFRQPVDPQLLGI
PDYFDIVKSPMDLSTIKRKLDTGQYQEPWQYVDDIWLMFNNAWLYNRKTSRVYKYCSKLSEVFEQEIDPV
MQSLGYCCGRKLEFSPQTLCCYGKQLCTIPRDATYYSYQNRYHFCEKCFNEIQGESVSLGDDPSQPQTT
INKEQFSKRKNDTLDPELFVECTECGRKMHQICVLHHEIIWPAGFVCDGCLKKSARTRKENKFSAKRLPS
TRLGTFLENRVNDFLRRQNHPESGEVTVRVVHASDKTVEVKPGMKARFVDSGEMAESFPYRTKALFAFE
EIDGVDLCFFGMHVQEYGSDCPPPNQRRVYISYLDSVHFFRPKCLRTAVYHEILIGYLEYVKKLGYTTGHI
WACPPSEGDDYIFHCHPPDQKIPKPKRLQEWYKKMLDKAVSERIVHDYKDIFKQATEDRLTSAKELPYFE
GDFWPNVLEESIKELEQEEEERKREENTSNESTDVTKGDSKNAKKKNNKKTSKNKSSLSRGNKKKPGMP
NVSNDLSQKLYATMEKHKEVFFVIRLIAGPAANSLPPIVDPDPLIPCDLMDGRDAFLTLARDKHLEFSSLRR
AQWSTMCMLVELHTQSQDGSDYKDDDDK

p300 core-dCas9-CBP core: amino acid sequence; Human p300 aa 1048-1664, SV-40, Streptococcus pyogenes Cas9 (D10A, H840A), Nuclear Localization Sequence, Human CBP aa 1087-1701, 1 X “Flag”

MIFKPEELRQALMPTLEALYRQDPESLPFRQPVDPQLLGIPDYFDIVKSPMDLSTIKRKLDTGQYQEPWQY
VDDIWLMFNNAWLYNRKTSRVYKYCSKLSEVFEQEIDPVMQSLGYCCGRKLEFSPQTLCCYGKQLCTIP

RDATYYSYQNRYHFCEKCFNEIQGESVSLGDDPSQPQTTINKEQFSKRKNDTLDPELFVECTECGRKMH
QICVLHHEIIWPAGFVCDGCLKKSARTRKENKFSAKRLPSTRLGTFLENRVNDFLRRQNHPESGEVTVRV
VHASDKTVEVKPGMKARFVDSGEMAESFPYRTKALFAFEEIDGVDLCFFGMHVQEYGSDCPPPNQRRV
YISYLDSVHFFRPKCLRTAVYHEILIGYLEYVKKLGYTTGHIWACPPSEGDDYIFHCHPPDQKIPKPKRLQE
WYKKMLDKAVSERIVHDYKDIFKQATEDRLTSAKELPYFEGDFWPNVLEESIKELEQEEEERKREENTSN
ESTDVTKGDSKNAKKKNNKKTSKNKSSLSRGNKKKPGMPNVSNDLSQKLYATMEKHKEVFFVIRLIAGPA
ANSLPPIVDPDPLIPCDLMDGRDAFLTLARDKHLEFSSLRRAQWSTMCMLVELHTQSQDGSPKKKRKVG
SSAATMDKKYSIGLAIGTNSVGWAVITDEYKVPSKKFKVLGNTDRHSIKKNLIGALLFDSGETAEATRLKRT
ARRRYTRRKNRICYLQEIFSNEMAKVDDSFFHRLEESFLVEEDKKHERHPIFGNIVDEVAYHEKYPTIYHL
RKKLVDSTDKADLRLIYLALAHMIKFRGHFLIEGDLNPDNSDVDKLFIQLVQTYNQLFEENPINASGVDAKAI
LSARLSKSRRLENLIAQLPGEKKNGLFGNLIALSLGLTPNFKSNFDLAEDAKLQLSKDTYDDDLDNLLAQIG
DQYADLFLAAKNLSDAILLSDILRVNTEITKAPLSASMIKRYDEHHQDLTLLKALVRQQLPEKYKEIFFDQSK
NGYAGYIDGGASQEEFYKFIKPILEKMDGTEELLVKLNREDLLRKQRTFDNGSIPHQIHLGELHAILRRQED
FYPFLKDNREKIEKILTFRIPYYVGPLARGNSRFAWMTRKSEETITPWNFEEVVDKGASAQSFIERMTNFD
KNLPNEKVLPKHSLLYEYFTVYNELTKVKYVTEGMRKPAFLSGEQKKAIVDLLFKTNRKVTVKQLKEDYFK
KIECFDSVEISGVEDRFNASLGTYHDLLKIIKDKDFLDNEENEDILEDIVLTLTLFEDREMIEERLKTYAHLFD
DKVMKQLKRRRYTGWGRLSRKLINGIRDKQSGKTILDFLKSDGFANRNFMQLIHDDSLTFKEDIQKAQVS
GQGDSLHEHIANLAGSPAIKKGILQTVKVVDELVKVMGRHKPENIVIEMARENQTTQKGQKNSRERMKRI
EEGIKELGSQILKEHPVENTQLQNEKLYLYYLQNGRDMYVDQELDINRLSDYDVDAIVPQSFLKDDSIDNK
VLTRSDKNRGKSDNVPSEEVVKKMKNYWRQLLNAKLITQRKFDNLTKAERGGLSELDKAGFIKRQLVETR
QITKHVAQILDSRMNTKYDENDKLIREVKVITLKSKLVSDFRKDFQFYKVREINNYHHAHDAYLNAVVGTAL
IKKYPKLESEFVYGDYKVYDVRKMIAKSEQEIGKATAKYFFYSNIMNFFKTEITLANGEIRKRPLIETNGETG
EIVWDKGRDFATVRKVLSMPQVNIVKKTEVQTGGFSKESILPKRNSDKLIARKKDWDPKKYGGFDSPTVA
YSVLVVAKVEKGKSKKLKSVKELLGITIMERSSFEKNPIDFLEAKGYKEVKKDLIIKLPKYSLFELENGRKRM
LASAGELQKGNELALPSKYVNFLYLASHYEKLKGSPEDNEQKQLFVEQHKHYLDEIIEQISEFSKRVILADA
NLDKVLSAYNKHRDKPIREQAENIIHLFTLTNLGAPAAFKYFDTTIDRKRYTSTKEVLDATLIHQSITGLYET
RIDLSQLGGDKRPAATKKAGQAKKKKGSIFKPEELRQALMPTLEALYRQDPESLPFRQPVDPQLLGIPDY
FDIVKNPMDLSTIKRKLDTGQYQEPWQYVDDVWLMFNNAWLYNRKTSRVYKFCSKLAEVFEQEIDPVMQ
SLGYCCGRKYEFSPQTLCCYGKQLCTIPRDAAYYSYQNRYHFCEKCFTEIQGENVTLGDDPSQPQTTISK
DQFEKKKNDTLDPEPFVDCKECGRKMHQICVLHYDIIWPSGFVCDNCLKKTGRPRKENKFSAKRLQTTRL
GNHLEDRVNKFLRRQNHPEAGEVFVRVVASSDKTVEVKPGMKSRFVDSGEMSESFPYRTKALFAFEEID
GVDVCFFGMHVQEYGSDCPPPNTRRVYISYLDSIHFFRPRCLRTAVYHEILIGYLEYVKKLGYVTGHIWAC
PPSEGDDYIFHCHPPDQKIPKPKRLQEWYKKMLDKAFAERIIHDYKDIFKQATEDRLTSAKELPYFEGDFW
PNVLEESIKELEQEEEERKKEESTAASETTEGSQGDSKNAKKKNNKKTNKNKSSISRANKKKPSMPNVSN
DLSQKLYATMEKHKEVFFVIHLHAGPVINTLPPIVDPDPLLSCDLMDGRDAFLTLARDKHWEFSSLRRSK
WSTLCMLVELHTQGQDGSDYKDDDDK

CBP core-dCas9-CBP core: amino acid sequence; Human CBP aa 1087-1701, SV-40, Streptococcus pyogenes Cas9 (D10A, H840A), Nuclear Localization Sequence, Human CBP aa 1087-1701, 1 X "Flag"

MIFKPEELRQALMPTLEALYRQDPESLPFRQPVDPQLLGIPDYFDIVKNPMDLSTIKRKLDTGQYQEPWQ
YVDDVWLMFNNAWLYNRKTSRVYKFCSKLAEVFEQEIDPVMQSLGYCCGRKYEFSPQTLCCYGKQLCTI
PRDAAYYSYQNRYHFCEKCFTEIQGENVTLGDDPSQPQTTISKDQFEKKKNDTLDPEPFVDCKECGRKM
HQICVLHYDIIWPSGFVCDNCLKKTGRPRKENKFSAKRLQTTRLGNHLEDRVNKFLRRQNHPEAGEVFVR
VVASSDKTVEVKPGMKSRFVDSGEMSESFPYRTKALFAFEEIDGVDVCFFGMHVQEYGSDCPPPNTRR
VYISYLDSIHFFRPRCLRTAVYHEILIGYLEYVKKLGYVTGHIWACPPSEGDDYIFHCHPPDQKIPKPKRLQ
EWYKKMLDKAFAERIIHDYKDIFKQATEDRLTSAKELPYFEGDFWPNVLEESIKELEQEEEERKKEESTAA
SETTEGSQGDSKNAKKKNNKKTNKNKSSISRANKKKPSMPNVSNDLSQKLYATMEKHKEVFFVIHLHAG
PVINTLPPIVDPDPLLSCDLMDGRDAFLTLARDKHWEFSSLRRSKWSTLCMLVELHTQGQDGSPKKKRK
VGSSAATMDKKYSIGLAIGTNSVGWAVITDEYKVPSKKFKVLGNTDRHSIKKNLIGALLFDSGETAEATRLK
RTARRRYTRRKNRICYLQEIFSNEMAKVDDSFFHRLEESFLVEEDKKHERHPIFGNIVDEVAYHEKYPTIY
HLRKKLVDSTDKADLRLIYLALAHMIKFRGHFLIEGDLNPDNSDVDKLFIQLVQTYNQLFEENPINASGVDA
KAILSARLSKSRRLENLIAQLPGEKKNGLFGNLIALSLGLTPNFKSNFDLAEDAKLQLSKDTYDDDLDNLLA
QIGDQYADLFLAAKNLSDAILLSDILRVNTEITKAPLSASMIKRYDEHHQDLTLLKALVRQQLPEKYKEIFFD
QSKNGYAGYIDGGASQEEFYKFIKPILEKMDGTEELLVKLNREDLLRKQRTFDNGSIPHQIHLGELHAILRR
QEDFYPFLKDNREKIEKILTFRIPYYVGPLARGNSRFAWMTRKSEETITPWNFEEVVDKGASAQSFIERMT
NFDKNLPNEKVLPKHSLLYEYFTVYNELTKVKYVTEGMRKPAFLSGEQKKAIVDLLFKTNRKVTVKQLKED
YFKKIECFDSVEISGVEDRFNASLGTYHDLLKIIKDKDFLDNEENEDILEDIVLTLTLFEDREMIEERLKTYAH
LFDDKVMKQLKRRRYTGWGRLSRKLINGIRDKQSGKTILDFLKSDGFANRNFMQLIHDDSLTFKEDIQKA
QVSGQGDSLHEHIANLAGSPAIKKGILQTVKVVDELVKVMGRHKPENIVIEMARENQTTQKGQKNSRERM
KRIEEGIKELGSQILKEHPVENTQLQNEKLYLYYLQNGRDMYVDQELDINRLSDYDVDAIVPQSFLKDDSID
NKVLTRSDKNRGKSDNVPSEEVVKKMKNYWRQLLNAKLITQRKFDNLTKAERGGLSELDKAGFIKRQLV
ETRQITKHVAQILDSRMNTKYDENDKLIREVKVITLKSKLVSDFRKDFQFYKVREINNYHHAHDAYLNAVV
GTALIKKYPKLESEFVYGDYKVYDVRKMIAKSEQEIGKATAKYFFYSNIMNFFKTEITLANGEIRKRPLIETN
GETGEIVWDKGRDFATVRKVLSMPQVNIVKKTEVQTGGFSKESILPKRNSDKLIARKKDWDPKKYGGFDS
PTVAYSVLVVAKVEKGKSKKLKSVKELLGITIMERSSFEKNPIDFLEAKGYKEVKKDLIIKLPKYSLFELENG
RKRMLASAGELQKGNELALPSKYVNFLYLASHYEKLKGSPEDNEQKQLFVEQHKHYLDEIIEQISEFSKRV
ILADANLDKVLSAYNKHRDKPIREQAENIIHLFTLTNLGAPAAFKYFDTTIDRKRYTSTKEVLDATLIHQSITG
LYETRIDLSQLGGDKRPAATKKAGQAKKKKGSIFKPEELRQALMPTLEALYRQDPESLPFRQPVDPQLLGI
PDYFDIVKNPMDLSTIKRKLDTGQYQEPWQYVDDVWLMFNNAWLYNRKTSRVYKFCSKLAEVFEQEIDP
VMQSLGYCCGRKYEFSPQTLCCYGKQLCTIPRDAAYYSYQNRYHFCEKCFTEIQGENVTLGDDPSQPQT
TISKDQFEKKKNDTLDPEPFVDCKECGRKMHQICVLHYDIIWPSGFVCDNCLKKTGRPRKENKFSAKRLQ
TTRLGNHLEDRVNKFLRRQNHPEAGEVFVRVVASSDKTVEVKPGMKSRFVDSGEMSESFPYRTKALFAF
EEIDGVDVCFFGMHVQEYGSDCPPPNTRRVYISYLDSIHFFRPRCLRTAVYHEILIGYLEYVKKLGYVTGHI
WACPPSEGDDYIFHCHPPDQKIPKPKRLQEWYKKMLDKAFAERIIHDYKDIFKQATEDRLTSAKELPYFEG

DFWPNVLEESIKELEQEEEERKKEESTAASETTEGSQGDSKNAKKKNNKKTNKNKSSISRANKKKPSMP
NVSNDLSQKLYATMEKHKEVFFVIHLHAGPVINTLPPIVDPDPLLSCDLMDGRDAFLTLARDKHWEFSSLR
RSKWSTLCMLVELHTQGQDGSDYKDDDDK
